# Correction: Identifying essential factors for energy-efficient walking control across a wide range of velocities in reflex-based musculoskeletal systems

**DOI:** 10.1371/journal.pcbi.1014482

**Published:** 2026-07-06

**Authors:** The PLOS Computational Biology Staff

The caption for Fig 9 is incorrect. The caption has been provided here.

The publisher apologizes for the error.

**Fig 9:**
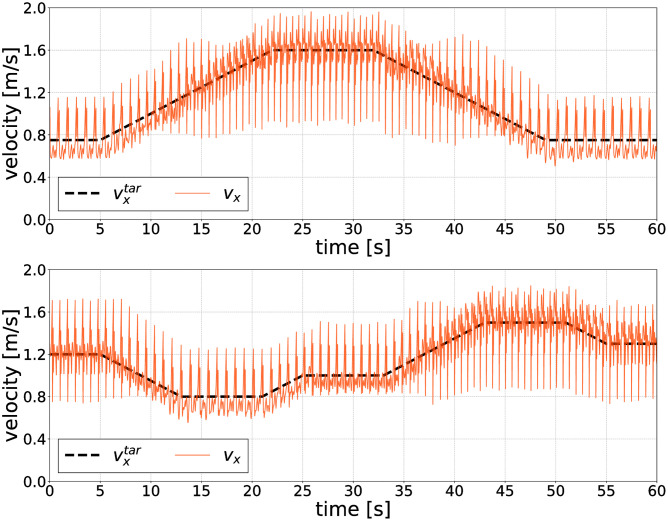
Time evolution of the generated walking speed (orange) in response to the target velocity, $\overset{tar}{\underset{x}{v}}$ (dotted line). $\overset{tar}{\underset{x}{v}}$ was adjusted with a change rate of 0.05 m/s per second. See S1 Video.
