# Supplementary material for: Identifying essential factors for energy-efficient walking control across a wide range of velocities in reflex-based musculoskeletal systems
Source: PLoS Comput Biol. 2024 Jan 19;20(1):e1011771. doi: 10.1371/journal.pcbi.1011771 (PMC10798509; doi:10.1371/journal.pcbi.1011771)
Supplement: S1 Appendix — (ZIP) [file pcbi.1011771.s001.zip › S1_Appendix.pdf]

Identifying essential factors for energy-efficient walking control across a wide range of velocities in reflex-based musculoskeletal systems

Supplemental Material

Shunsuke Koseki, Mitsuhiro Hayashibe, and Dai Owaki

1: Reflex-based control

The reflex-based controller employed in this study is identical in principle to that of Wang et al [1]. The controller computes the muscle stimulation, u_i, for each muscle, i, by utilizing sensory feedback with a time delay of Δt as input. Humans use the signals from the mechanoreceptors in the foot for the control of phase transitions [2]. Similarly, the control law switches depending on whether the leg is in the stance or swing phase, and additional stimulation is added during the late stance phase and late swing phase.

Stance Phase

During the stance phase, the stimulation, u_i, to each muscle actuator is calculated as follows:

$$
\begin{aligned}
u_{SOL} &= p_{SOL} + G_{SOL}\tilde{F}_{SOL}(t-\Delta t_l), &\text{(S1)}\\
u_{TA} &= p_{TA} + \max\{0, G_{TA\_st}(l_{TA}(t-\Delta t_l) - l^{tar}_{TA\_st}))\} - G_{SOL\_TA}\tilde{F}_{SOL}(t-\Delta t_l), &\text{(S2)}\\
u_{GAS} &= p_{GAS} + G_{GAS}\tilde{F}_{GAS}(t-\Delta t_l), &\text{(S3)}\\
u_{VAS} &= p_{VAS} + G_{VAS}\tilde{F}_{VAS}(t-\Delta t_m) + f_{VAS}, &\text{(S4)}\\
f_{VAS} &= \begin{cases} 0 & (\theta_k < \theta_k^{off}) \\ k_{\theta_k}\{\theta_k(t-\Delta t_{VAS}) - \theta_k^{off}\} & (\theta_k \geq \theta_k^{off}) \end{cases}, &\text{(S5)}\\
u_{HAM} &= p_{HAM} + \max\{0, K_{HAM}(\theta_t(t-\Delta t_s) - \theta_t^{tar}) + D_{HAM}\dot{\theta}_t\}, &\text{(S6)}\\
u_{RF} &= p_{RF}, &\text{(S7)}\\
u_{GLU} &= p_{GLU} + \max\{0, K_{GLU}(\theta_t(t-\Delta t_s) - \theta_t^{tar}) + D_{GLU}\dot{\theta}_t\}, &\text{(S8)}\\
u_{HFL} &= p_{HFL} + \max\{0, K_{HFL}(\theta_t^{tar} - \theta_t(t-\Delta t_s)) + D_{HFL}\dot{\theta}_t\}, &\text{(S9)}
\end{aligned}
$$

where p_i are positive constant control parameters that are approximate to zero. The time delays, Δt_l, Δt_m, and Δt_s, are set to 20, 10, and 5 ms, respectively. The shorter transmission pathway to the spinal cord results in smaller time delays; therefore, the muscles near the hip joint have minimal time delays. Positive force feedback at GLU, VAS, and SOL contributes to compliant leg behavior. When the gains, denoted as G, are set higher, the stance leg emulates an inverted pendulum model. Conversely, when the gains are set at smaller values, it behaves as a spring-inverted pendulum model [3]. Positive length feedback at TA prevents overextension of the ankle joint. This action is suppressed by negative force feedback from the SOL, which serves to extend the ankle joint during a push-off. Positive force feedback at GAS contributes to push-off and prevents overextension of the knee joint. Muscles around the hip joint, namely HFL, GLU, and HAM, function together to maintain the reference lean angle θ_t^{tar} through muscle-driven PD controls, thus stabilizing the torso. The term, f_{VAS}, prevents knee hyperextensions during the mid-stance phase. Stimulation of the VAS is suppressed when the knee joint angle, θ_k, exceeds the threshold angle, θ_k^{off}. During the swing initiation phase, when the hip extension angle, θ_h, exceeds θ_{SI}, as depicted in Fig AI, constant stimulation values s_i that are greater than zero are added to the VAS, RF, GLU, and HFL to encourage swing initiation [4].

$$
\begin{aligned}
u_{VAS} &\leftarrow u_{VAS} - s_{VAS}, && \text{(S10)}\\
u_{RF} &\leftarrow u_{RF} + s_{RF}, && \text{(S11)}\\
u_{GLU} &\leftarrow u_{GLU} - s_{GLU}, && \text{(S12)}\\
u_{HFL} &\leftarrow u_{HFL} + s_{HFL}. && \text{(S13)}
\end{aligned}
$$

Swing Phase

During the swing phase, the stimulation, u_i, to each muscle actuator is calculated as follows:

$$
\begin{aligned}
u_{SOL} &= q_{SOL}, && \text{(S14)}\\
u_{TA} &= q_{TA} + \max\{0, G_{TA\_sw}(l_{TA}(t - \Delta t_l) - l^{tar}_{TA\_sw})\}, && \text{(S15)}\\
u_{GAS} &= q_{GAS}, && \text{(S16)}\\
u_{VAS} &= q_{VAS}, && \text{(S17)}\\
u_{HAM} &= q_{HAM} + G_{HAM}\tilde{F}_{HAM}(t - \Delta t_s), && \text{(S18)}\\
u_{RF} &= q_{RF}, && \text{(S19)}\\
u_{GLU} &= q_{GLU} + G_{GLU}\tilde{F}_{GLU}(t - \Delta t_s), && \text{(S20)}\\
u_{HFL} &= q_{HFL} + \max\{0, G_{HFL}(l_{HFL}(t - \Delta t_s) - l^{tar}_{HFL})\} \\
&\quad - \max\{0, G_{HAM\_HFL}(l_{HAM}(t - \Delta t_s) - l^{tar}_{HAM})\} + f_{HFL}, && \text{(S21)}\\
f_{HFL} &= k_{lean}\{\theta^{tar}_t - \theta_t(t - \Delta t_{HFL})\}, && \text{(S22)}
\end{aligned}
$$

where q_i are positive constant control parameters similar to p_i and also approximate zero. Positive length feedback at HFL facilitates leg swing, which is suppressed by negative length feedback from HAM in the mid-swing phase. The term f_{HFL} stimulates the HFL according to the current torso angle, θ_t, and the torso target angle, θ^{tar}_t. The required protraction speed relies on the torso lean. Positive force feedback at GLU and HAM applies braking force to the swinging leg, transferring some of the angular momentum into leg lowering and retraction. Positive length feedback at TA raises the toes, ensuring sufficient clearance between the feet and the ground. During the stance preparation phase, when the hip flexion angle, θ_h, exceeds θ_{SP}, as depicted in Fig AI, the swinging leg transitions into stance preparation. Muscle-driven PD controls are integrated into the VAS, GLU, and HFL to produce the desired pose for touchdown in this phase.

$$
\begin{aligned}
u_{VAS} &\leftarrow u_{VAS} \\
&\quad + \max\{0, K_{SP\_VAS}(\theta_k(t - \Delta t_m) - \theta^{tar}_k) + D_{SP\_HFL}\dot{\theta}_k(t - \Delta t_m)\}, && \text{(S23)}\\
u_{GLU} &\leftarrow u_{GLU} \\
&\quad + \max\{0, K_{SP\_GLU}(\theta^{tar}_h - \theta_h(t - \Delta t_m)) + D_{SP\_GLU}\dot{\theta}_h(t - \Delta t_m)\}, && \text{(S24)}\\
u_{HFL} &\leftarrow u_{HFL} \\
&\quad + \max\{0, K_{SP\_HFL}(\theta_h(t - \Delta t_m) - \theta^{tar}_h) + D_{SP\_HFL}\dot{\theta}_h(t - \Delta t_m)\}. && \text{(S25)}
\end{aligned}
$$

To reproduce a stable gait, the target hip angle, θ^{tar}_h, is adjusted according to the balance feedback law (SIMBICON) [5]:

$$\theta^{tar}_h = \theta^{tar}_h - c_d d + c_v v_x, \qquad \text{(S26)}$$

where d represents the horizontal distance from the stance ankle to the hip segment, as depicted in Fig AII. v_x represents the horizontal velocity of the model, and c_d and c_v are positive constant parameters. This balance feedback law can be interpreted as the supraspinal control that uses sensory information from the vestibular organs.

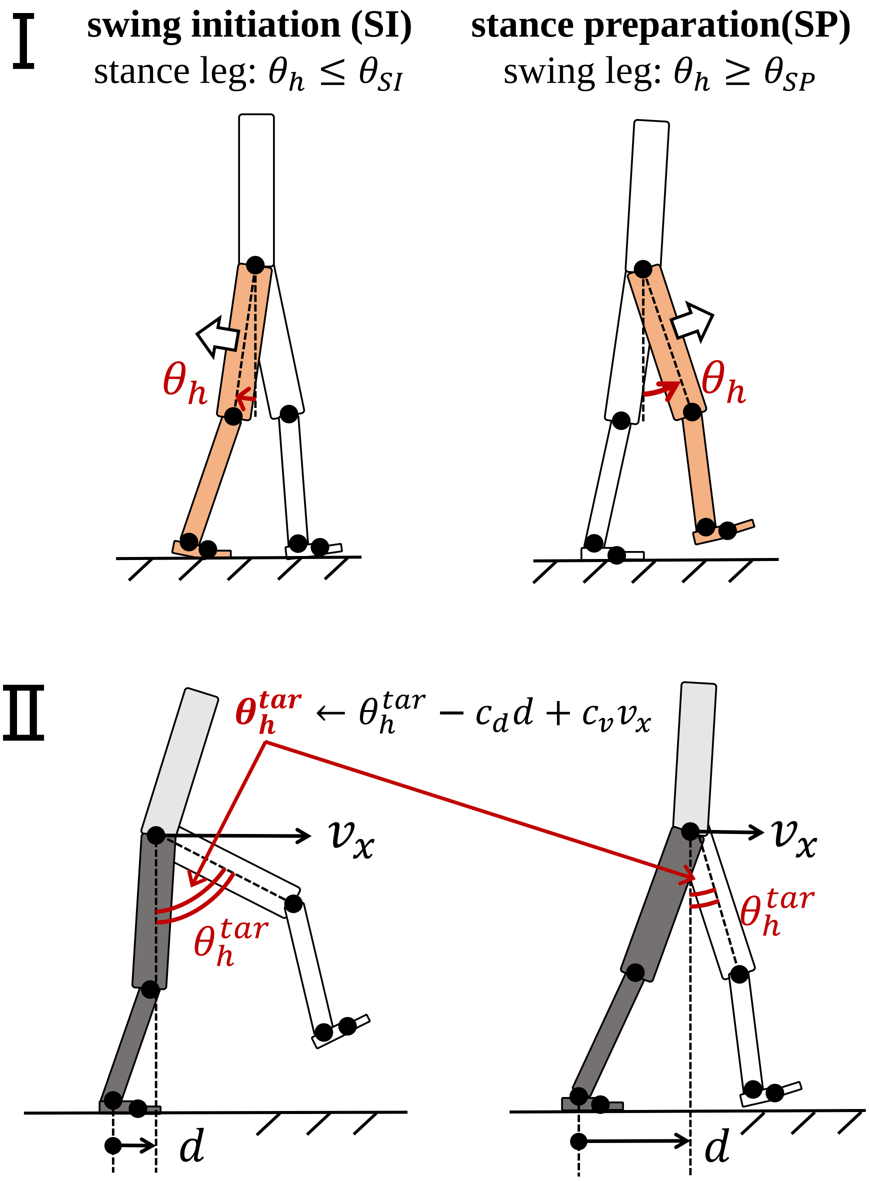

Fig A: **Description of the reflex-based control. I. Transition conditions of swing initiation (SI) and stance preparation (SP) phases.** The SI phase begins when the hip extension angle, θ_h, of the stance leg exceeds a predetermined threshold, θ_{SI}. The SP phase begins when the hip flexion angle, θ_h, of the swing leg exceeds the threshold, θ_{SP}. **II. The diagram of the balance feedback law.** The feedback control adjusts the target hip angle, θ_h^{tar}, depending on the horizontal distance from the stance ankle to the hip segment, d, and the horizontal model velocity, v_x. For instance, when d is small or v_x is large, θ_h^{tar} is increased, resulting in the foot being placed more forward to prevent falling, as depicted on the left. Conversely, when d is large or v_x is small, θ_h^{tar} is decreased.

2: computation of F_l, F_v, and F_p

MuJoCo provides a set of tools for modeling biological muscles [6]. In the provided tool set, F_l, F_v, and F_p (Eq.(3)) are defined. The force-length relationship, F_l, is represented as:

$$F_l(\tilde{l}) = F_{l1}(\tilde{l}) + 0.15F_{l2}(\tilde{l}), \tag{S27}$$

where

$$F_{l1}(\tilde{l}) = \begin{cases} 0 & (\tilde{l} \leq l_{min}) \\ 2(\frac{\tilde{l}-l_{min}}{1-l_{min}})^2 & (l_{min}\tilde{l} \leq 0.5l_{min} + 0.5) \\ 1 - 2(\frac{1-\tilde{l}}{1-l_{min}})^2 & (0.5l_{min} + 0.5 \leq \tilde{l} \leq 1) \\ 1 - 2(\frac{1-\tilde{l}}{1-l_{min}})^2 & (1 \leq \tilde{l} \leq 0.5l_{max} + 0.5) \\ 2(\frac{l_{max}-\tilde{l}}{l_{max}-1})^2 & (0.5l_{max} + 0.5 \leq \tilde{l} \leq l_{max}) \\ 0 & (\tilde{l} \geq l_{max}) \end{cases}, \tag{S28}$$

$$F_{l2}(\tilde{l}) = \begin{cases} 0 & (\tilde{l} \leq l_{min}) \\ 8(\frac{\tilde{l}-l_{min}}{0.95-l_{min}})^2 & (l_{min} \leq \tilde{l} \leq 0.75l_{min} + 0.2375) \\ 1 - 8(\frac{0.5l_{min}+0.475-\tilde{l}}{0.95-l_{min}})^2 & (0.75l_{min} + 0.2375 \leq \tilde{l} \leq 0.5l_{min} + 0.475) \\ 1 - 8(\frac{\tilde{l}-0.5l_{min}-0.475}{1-l_{min}})^2 & (0.5l_{min} + 0.475 \leq \tilde{l} \leq 0.25l_{min} + 0.7125) \\ 8(\frac{0.95-\tilde{l}}{0.95-l_{min}})^2 & (0.25l_{min} + 0.7125 \leq \tilde{l} \leq 0.95) \\ 0 & (\tilde{l} \geq 0.95) \end{cases}, \tag{S29}$$

with l_{min} and l_{max} being constants set to 0.5 and 1.6, respectively. The passive force, Fp, is always present regardless of activation and is defined as:

$$F_p(\tilde{l}) = \begin{cases} 0 & (\tilde{l} \leq 1) \\ 2f_{pmax}(\frac{\tilde{l}-1}{l_{max}-1})^2 & (1 \leq \tilde{l} \leq 0.5l_{max} + 0.5) \\ 0.5f_{pmax}(\frac{3\tilde{l}-l_{max}-2}{l_{max}-1})^2 & (\tilde{l} \geq 0.5l_{max} + 0.5) \end{cases}, \tag{S30}$$

l_{max} and f_{pmax} are constants. $l_{max} = 1.6$ is equal to in F_l and f_{pmax} is set to 1.3. The force-velocity relationship, F_v, is defined as:

$$F_v(\tilde{v}) = \begin{cases} 0 & (\tilde{v} \leq -v_{max}) \\ (\frac{\tilde{v}}{v_{max}} + 1)^2 & (-v_{max} \leq \tilde{v} \leq 0) \\ f_{vmax} - \frac{(f_{vmax}-1-\frac{\tilde{v}}{v_{max}})^2}{f_{vmax}-1} & (0 \leq \tilde{v} \leq v_{max}(f_{vmax} - 1)) \\ f_{vmax} & (\tilde{v} \geq v_{max}(f_{vmax} - 1)) \end{cases}, \tag{S31}$$

where v_{max} and f_{fmax} are constants set to 1.5 and 1.2, respectively. The shapes of these functions are illustrated in Fig B.

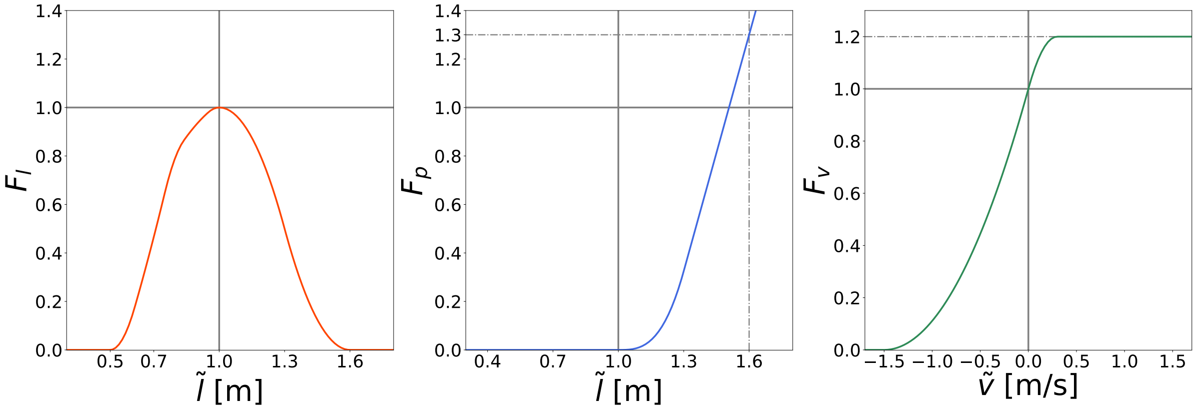

Fig B: **The shapes of $F_l(\tilde{l})$, $F_p(\tilde{l})$, and $F_v(\tilde{v})$**

3: Details of the Control Parameters

In total, there are 54 control parameters for these reflex controls. Table A lists them and the ranges set during the optimization.

Table A: Control Parameters and the Ranges Set During the Optimization

parameter	min	max	parameter	min	max	parameter	min	max
p_{SOL}	0	0.05	G_{SOL_TA}	0	5.0	K_{HFL}	0	8.0
p_{TA}	0	0.05	G_{GAS}	0	5.0	D_{HAM}	0	1.0
p_{GAS}	0	0.15	G_{VAS}	0	5.0	D_{GLU}	0	1.0
p_{VAS}	0	0.15	G_{HAM}	0	5.0	D_{HFL}	0	1.0
p_{HAM}	0	0.05	G_{GLU}	0	5.0	s_{GLU}	0	1.0
p_{RF}	0	0.05	G_{HFL}	0	10.0	s_{HFL}	0	1.0
p_{GLU}	0	0.05	G_{HAM_HFL}	0	10.0	s_{RF}	0	1.0
p_{HFL}	0	0.05	$l^{tar}_{TA\_st}$	0	0.18	s_{VAS}	0	1.0
q_{SOL}	0	0.05	$l^{tar}_{TA\_sw}$	0	0.18	θ_{DS}	-0.45	0.2
q_{TA}	0	0.05	l^{tar}_{HFL}	0	0.22	θ_{SP}	-0.1	1.0
q_{GAS}	0	0.05	l^{tar}_{HAM}	0	0.55	K_{SP_VAS}	0	3.5
q_{VAS}	0	0.05	θ^{off}_k	2.7	3.15	K_{SP_GLU}	0	3.5
q_{HAM}	0	0.05	θ^{tar}_t	0	0.6	K_{SP_HFL}	0	3.5
q_{RF}	0	0.05	θ^{tar}_h	0.5	1.5	D_{SP_VAS}	0	3.0
q_{GLU}	0	0.05	θ^{tar}_k	2.7	3.15	D_{SP_GLU}	0	1.0
q_{HFL}	0	0.05	k_{θ_k}	0	5.0	D_{SP_HFL}	0	1.0
G_{SOL}	0	5.0	k_{lean}	0	5.0	c_d	0	0.4
G_{TA_st}	0	8.0	K_{HAM}	0	8.0	c_v	0	0.2
G_{TA_sw}	0	8.0	K_{GLU}	0	8.0			

4: Computation of the metabolic energy

The metabolic energy, J, was calculated as the metabolic energies expended by all muscles, designed based on previous studies [7, 1]. J is defined as the integral of the energy expenditure rate from the initial state to the termination of the trial:

$$J = \int_0^{T'} (\dot{B} + \dot{A} + \dot{M} + \dot{S} + \dot{W})dt \tag{S32}$$

where $\dot{B}$ represents the basal metabolic energy rate,

$$\dot{B} = 1.51 * mass \tag{S33}$$

where $mass$ denotes the model mass. $\dot{A}$ represents the muscle activation heat rate, which represents heat loss caused by large stimulation to the muscles. $\dot{A}$ is determined as follows:

$$\dot{A} = \sum_i^{muscles} muscle\_mass_i \cdot f_A(u_i) \tag{S34}$$

$muscle\_mass_i$ represents the mass of each muscle i. However, Muscle actuators in MuJoCo possess no mass. Thus, we set the virtual mass to muscles. $muscle\_mass_i$ is computed as the product of the square of the muscle radius r_i, and their rest length l_i^0:

$$muscle\_mass_i = \rho r_i^2 l_i^0 \tag{S35}$$

ρ denotes the muscle density 1016kg/m^3. The values of r_i and l_i^0 for each muscle i are shown in Table B. $f_A(u_i)$ is computed as:

$$f_A(u_i) = 40\lambda_i sin(\frac{\pi}{2}u_i) + 133(1-\lambda_i)(1-cos(\frac{\pi}{2}u_i)), \tag{S36}$$

where u_i represents the stimulation, λ_i is a constant for each muscle. λ_i is presented in Table B. $\dot{M}$ represents the muscle maintenance heat rate, which models the heat loss incurred to maintain muscle contraction.

$$\dot{M} = \sum_i muscle\_mass_i \cdot g(\tilde{l}_i) f_M(a_i) \tag{S37}$$

where

$$g(\tilde{l}_i) = \begin{cases} 0.5 & (\tilde{l}_i \leq 0.5) \\ \tilde{l}_i & (0.5 < \tilde{l}_i \leq 1.0) \\ -2\tilde{l}_i + 3 & (1.0 < \tilde{l}_i \leq 1.5) \\ 0 & (\tilde{l}_i \geq 1.5) \end{cases}, \tag{S38}$$

$$f_M(a_i) = 74\lambda_i sin(\frac{\pi}{2}a_i) + 111(1-\lambda_i)(1-cos(\frac{\pi}{2}a_i)), \tag{S39}$$

where a_i represents the current activation level for each muscle. $\dot{S}$ represents the muscle shortening heat rate, which models the heat loss incurred during muscle fiber shortening.

$$\dot{S} = 0.25 \sum_i F_i max(0, -v_i) \tag{S40}$$

W denotes the positive mechanical work rate and is defined as the same as $\dot{S}$

$$\dot{W} = \sum_i F_i max(0, -v_i) \tag{S41}$$

Table B: Muscle Actuator Parameters

	SOL	TA	GAS	VAS	HAM	RF	GLU	HFL
l^0 [cm]	27.2	17.3	48.1	30.3	52.2	65.5	23.7	20.8
r [cm]	2.58	1.42	1.46	4.44	3.03	1.63	2.95	3.64
λ	0.810	0.700	0.540	0.500	0.440	0.423	0.500	0.500

5: Hadamard product

For instance, given matrices $\boldsymbol{A} = (a_{ij}) \in \mathbb{R}^{m\times n}$ and $\boldsymbol{B} = (b_{ij}) \in \mathbb{R}^{m\times n}$, the Hadamard product $\boldsymbol{A} \otimes \boldsymbol{B}$ is calculated as follows:

$$\boldsymbol{A} \otimes \boldsymbol{B} = \begin{bmatrix} a_{11} & a_{12} & \dots & a_{1n} \\ a_{21} & a_{22} & \dots & a_{2n} \\ \vdots & \vdots & \ddots & \vdots \\ a_{m1} & a_{m2} & \dots & a_{mn} \end{bmatrix} \otimes \begin{bmatrix} b_{11} & b_{12} & \dots & b_{1n} \\ b_{21} & b_{22} & \dots & b_{2n} \\ \vdots & \vdots & \ddots & \vdots \\ b_{m1} & b_{m2} & \dots & b_{mn} \end{bmatrix}, \tag{S42}$$

$$= \begin{bmatrix} a_{11}b_{11} & a_{12}b_{12} & \dots & a_{1n}b_{1n} \\ a_{21}b_{21} & a_{22}b_{22} & \dots & a_{2n}b_{2n} \\ \vdots & \vdots & \ddots & \vdots \\ a_{m1}b_{m1} & a_{m2}b_{m2} & \dots & a_{mn}b_{mn} \end{bmatrix}, \tag{S43}$$

6: Detailed reformulation of eq.(32)

$\boldsymbol{\beta} \otimes \boldsymbol{V}\boldsymbol{\omega_i}$ can be reformulated as follows:

$$\boldsymbol{\beta} \otimes \boldsymbol{V}\boldsymbol{\omega_i} = \begin{bmatrix} \beta_1(\omega_{i0}v_{x_1}^{0} + \omega_{i1}v_{x_1}^{1} + ... + \omega_{im}v_{x_1}^{m}) \\ \beta_2(\omega_{i0}v_{x_2}^{0} + \omega_{i1}v_{x_2}^{1} + ... + \omega_{im}v_{x_2}^{m}) \\ \vdots \\ \beta_n(\omega_{i0}v_{x_n}^{0} + \omega_{i1}v_{x_n}^{1} + ... + \omega_{im}v_{x_n}^{m}) \end{bmatrix}, \tag{S44}$$

$$= \begin{bmatrix} \beta_1 v_{x_1}^{0}\cdot\omega_{i0} + \beta_1 v_{x_1}^{1}\cdot\omega_{i1} + ... + \beta_1 v_{x_1}^{m}\cdot\omega_{im} \\ \beta_2 v_{x_2}^{0}\cdot\omega_{i0} + \beta_2 v_{x_2}^{1}\cdot\omega_{i1} + ... + \beta_2 v_{x_2}^{m}\cdot\omega_{im} \\ \vdots \\ \beta_n v_{x_n}^{0}\cdot\omega_{i0} + \beta_n v_{x_n}^{1}\cdot\omega_{i1} + ... + \beta_n v_{x_n}^{m}\cdot\omega_{im} \end{bmatrix}, \tag{S45}$$

$$= \begin{bmatrix} \beta_1 v_{x_1}^{0} & \beta_1 v_{x_1}^{1} & \dots & \beta_1 v_{x_1}^{m} \\ \beta_2 v_{x_2}^{0} & \beta_2 v_{x_2}^{1} & \dots & \beta_2 v_{x_2}^{m} \\ \vdots & \vdots & \ddots & \vdots \\ \beta_n v_{x_n}^{0} & \beta_n v_{x_n}^{1} & \dots & \beta_n v_{x_n}^{m} \end{bmatrix} \begin{bmatrix} \omega_{i0} \\ \omega_{i1} \\ \vdots \\ \omega_{im} \end{bmatrix}, \tag{S46}$$

$$= (\boldsymbol{B} \otimes \boldsymbol{V})\boldsymbol{\omega_i}, \tag{S47}$$

thus

$$\boldsymbol{\beta} \otimes \boldsymbol{V}\boldsymbol{\omega_i} = (\boldsymbol{B} \otimes \boldsymbol{V})\boldsymbol{\omega_i}, \tag{S48}$$

6: Detailed description of the dataset collection for polynomial regression

We run two programs in parallel to collect the data efficiently. Within one thread, initially, the target velocity, v_x^{tar}, in cost function, f (Eq. (7)), is set to 1.3 m/s. Then, v_x^{tar} is slightly increased to $v_x^{tar} + \Delta v_x$, and the corresponding values around the updated target velocity are collected. This process is repeated until v_x^{tar} reaches the upper limit of the target velocity, $v_{x\,max}^{tar}$. More specifically, in this study, Δv_x and $v_{x\,max}^{tar}$ were set to 0.1 m/s and 2.0 m/s. Therefore, the target velocity, v_x^{tar}, is incrementally changed to 1.3, 1.4, 1.5, 1.6, 1.7, 1.8, 1.9, 2.0 m/s with the interval $G = 300$, which is a generation number in CMA-ES.

Within the other thread, the target velocity, v_x^{tar} is initially set to 1.2 m/s. Then, v_x^{tar} is slightly decreased to $v_x^{tar} - \Delta v_x$ and this process is also repeated until v_x^{tar} reaches the lower limit of the target velocity, $v_{x\,min}^{tar}$. Δv_x and $v_{x\,max}^{tar}$ were set to 0.1 m/s and 0.4 m/s. Hence, v_x^{tar} is incrementally changed to 1.2, 1.1, 1.0, 0.9, 0.8, 0.7, 0.6, 0.5, 0.4 m/s.

Then, the collected dataset in both programs are combined and used for polynomial regression. Fig C shows the flow used to collect the dataset.

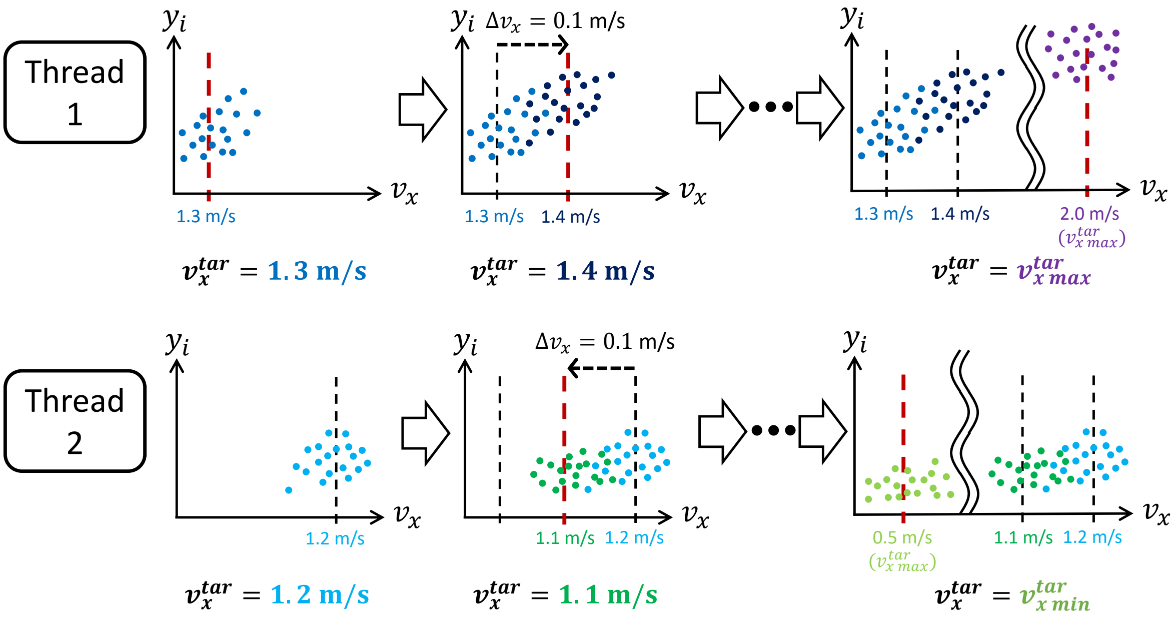

Fig C: Flow for collecting data for polynomial regression.

7: Convergence curve

Fig D illustrates the convergence curve for the different target velocities, v_x^{tar}, in the optimization process.

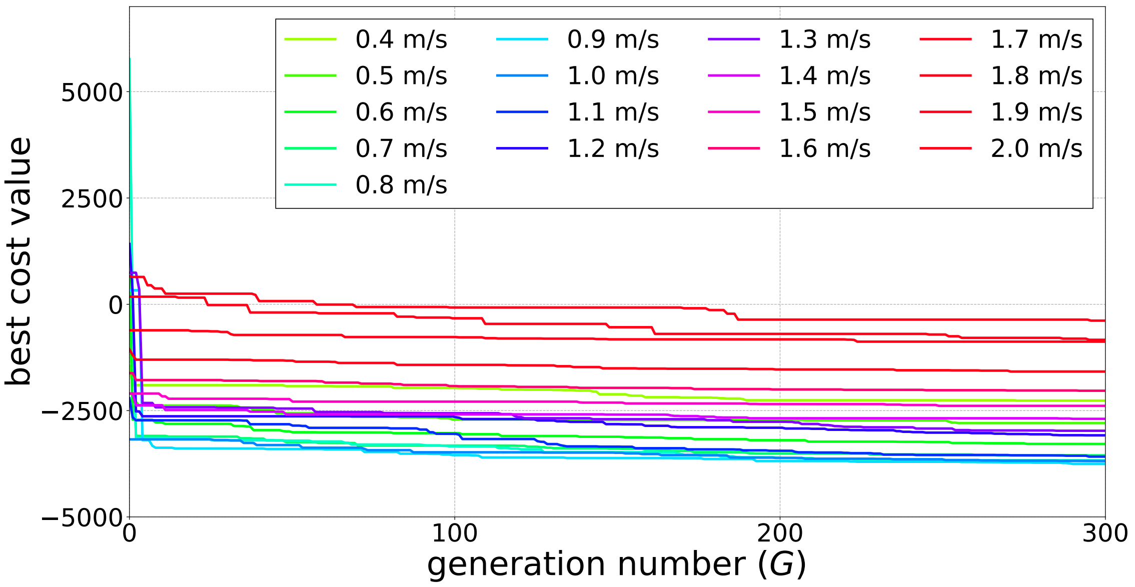

Fig D: **Convergence curve** The vertical axis indicates the calculated minimum value of the cost function, f (Eq.(7)).

8: Generated walking velocities for input target velocities

Fig E illustrates the target velocities v_x^{tar} and the actual walking speeds v_x. The gait was generated through the optimized functions derived with $A = 10^6$. Red stars indicate the measured velocity for the input target velocity. The points close to the dotted line ($v_x = v_x^{tar}$) indicate that it follows the target velocity well.

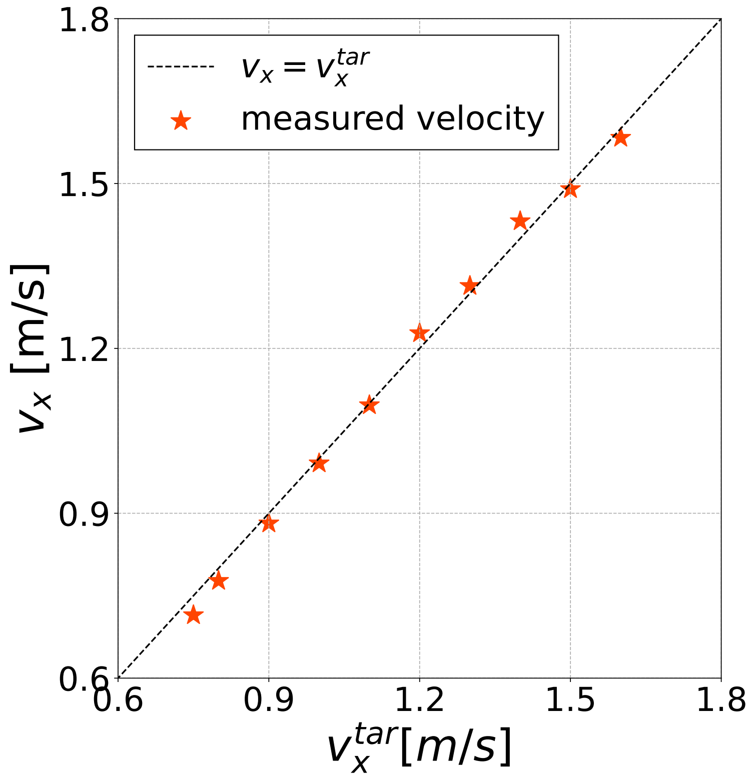

Fig E: The actual generated walking velocities v_x for input target velocities v_x^{tar}

9: Generated gaits under different setting parameters

To ensure the robustness of this conclusion, we have generated gaits in the various settings as described below:

- **shorten segment length by 20% (different body structure)**
  All segment was shortened and reduced their mass by 20%. We have conducted two different optimizations and found that steady walking was generated for $v_x^{tar} = 0.6 - 1.0\,\mathrm{m/s}$ by using the optimized functions derived with $A = 1 - 10^6$.

- **double the time delay of sensory information transmission to the controller (different neural system)**
  Sensory information transmissions to the controller include a time delay, Δt, with shorter transmission pathways to the spinal cord resulting in smaller values. Originally, the time delays, Δt_l, Δt_m, and Δt_s, are set to 20, 10, and 5 ms, respectively. In this setting, Δt_l, Δt_m, and Δt_s, are set to 40, 20, and 10 ms, respectively. We have conducted two optimizations and found that steady walking was generated for $v_x^{tar} = 0.8 - 1.7\,\mathrm{m/s}$ and $v_x^{tar} = 1.0 - 1.6\,\mathrm{m/s}$ by using the optimized functions derived with $A = 1 - 10^2$. It should be noted that the generated gaits in this setting were unstable and stable gaits were not generated at specific target velocities.

- **change the weight coefficients in the objective cost function (Eq.(31)), $\alpha_E = 2500$, $\alpha_v = 5$, and $\alpha_t = 0$ (different cost function)**
  Originally, the dataset was collected with $\alpha_E = 5000$, $\alpha_v = 5$, and $\alpha_t = 1.0$. Decreased α_E and α_t led to optimization to collect the dataset with an emphasis on tracking the target speed. We have conducted two optimizations and found that steady walking was generated for $v_x^{tar} = 0.8 - 1.7\,\mathrm{m/s}$ and $v_x^{tar} = 0.7 - 1.6\,\mathrm{m/s}$ by using the optimized functions derived with $A = 1 - 10^6$.

10: Foot penetrating the ground on the low-impedance ground

The musculoskeletal model's foot penetrated when the floor impedance was set to low as shown in Fig F. Therefore, we set the floor impedance set to high. While the high-impedance floor setting brought extreme peaks in the GRF profile, given the extreme peaks in GRF do not appear to affect kinematics significantly and the difficulty of modeling contact in the simulation environment, we consider the influence of the extreme peaks in GRF on the musculoskeletal model's movement to be small and acceptable.

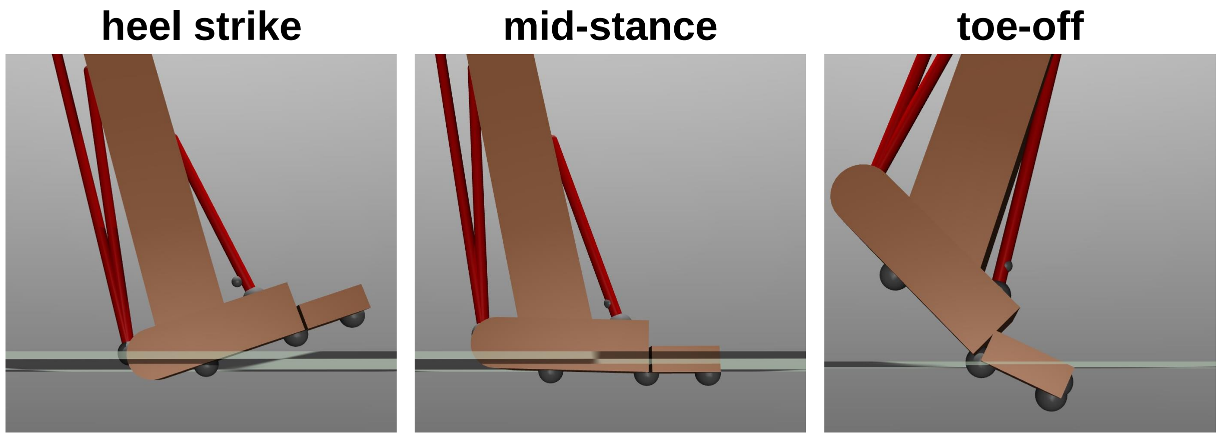

Fig F: Foot penetrating the ground on the low-impedance ground.

11: Trajectory of the hip segment height from the ground

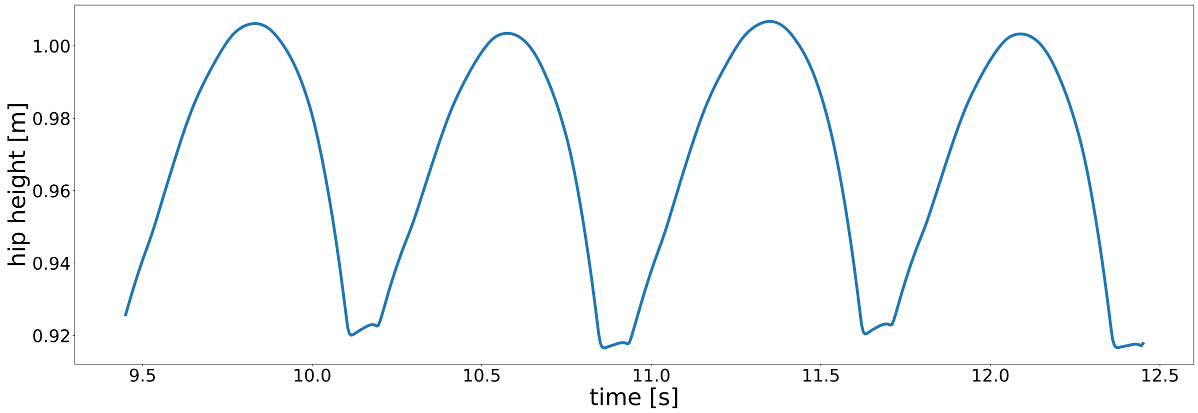

Fig G: Trajectory of the hip segment height from the ground. v_x^{tar} was set to 1.25 m/s. The curve was sinusoidal, corresponding to humans [8].

References

[1] Jack M Wang, Samuel R Hamner, Scott L Delp, and Vladlen Koltun. Optimizing locomotion controllers using biologically-based actuators and objectives. ACM Transactions on Graphics (TOG), Vol. 31, No. 4, pp. 1–11, 2012.

[2] Hartmut Geyer and Hugh Herr. A muscle-reflex model that encodes principles of legged mechanics produces human walking dynamics and muscle activities. IEEE Transactions on neural systems and rehabilitation engineering, Vol. 18, No. 3, pp. 263–273, 2010.

[3] Hartmut Geyer, Andre Seyfarth, and Reinhard Blickhan. Compliant leg behaviour explains basic dynamics of walking and running. Proceedings of the Royal Society B: Biological Sciences, Vol. 273, No. 1603, pp. 2861–2867, 2006.

[4] Seungmoon Song and Hartmut Geyer. Regulating speed and generating large speed transitions in a neuromuscular human walking model. 2012 IEEE International Conference on Robotics and Automation, pp. 511–516, 2012.

[5] KangKang Yin, Kevin Loken, and Michiel Van de Panne. Simbicon: Simple biped locomotion control. ACM Transactions on Graphics (TOG), Vol. 26, No. 3, pp. 105–es, 2007.

[6] MuJoCo Modeling. Muscle actuators, 2023. `https://mujoco.readthedocs.io/en/latest/modeling.html#cmuscle`.

[7] Frank Clayton Anderson III. A dynamic optimization solution for a complete cycle of normal gait. The University of Texas at Austin, 1999.

[8] Thys H Cavagna GA and Zamboni A. The sources of external work in level walking and running. J Physiol, Vol. 262, No. 3, pp. 639–657, 1976.
