# Supplementary figures and images for: Identifying essential factors for energy-efficient walking control across a wide range of velocities in reflex-based musculoskeletal systems
Source: PLoS Comput Biol. 2024 Jan 19;20(1):e1011771. doi: 10.1371/journal.pcbi.1011771 (PMC10798509; doi:10.1371/journal.pcbi.1011771)

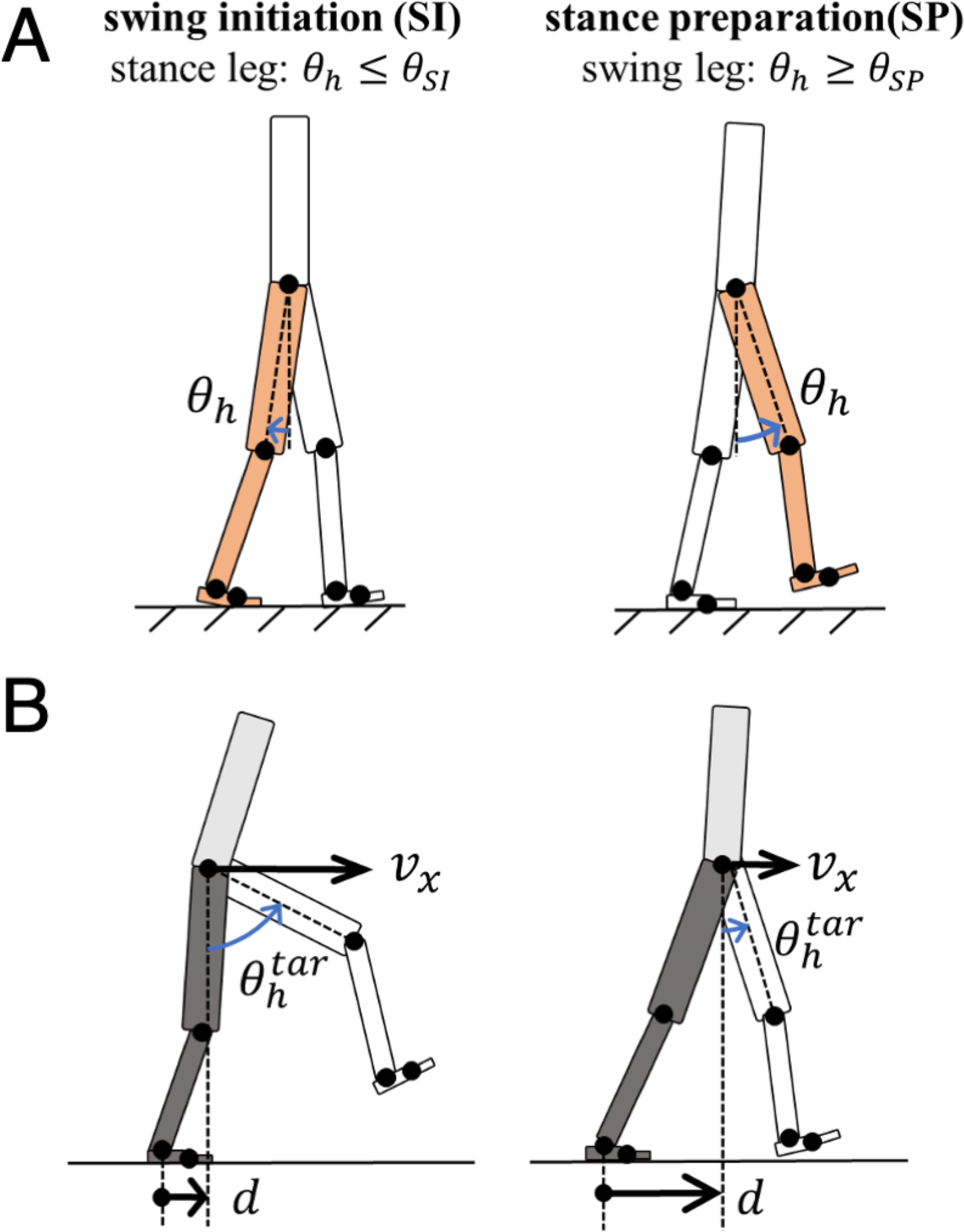

Supplement: S1 Appendix — (ZIP) [file pcbi.1011771.s001.zip › S1_Fig.tif]

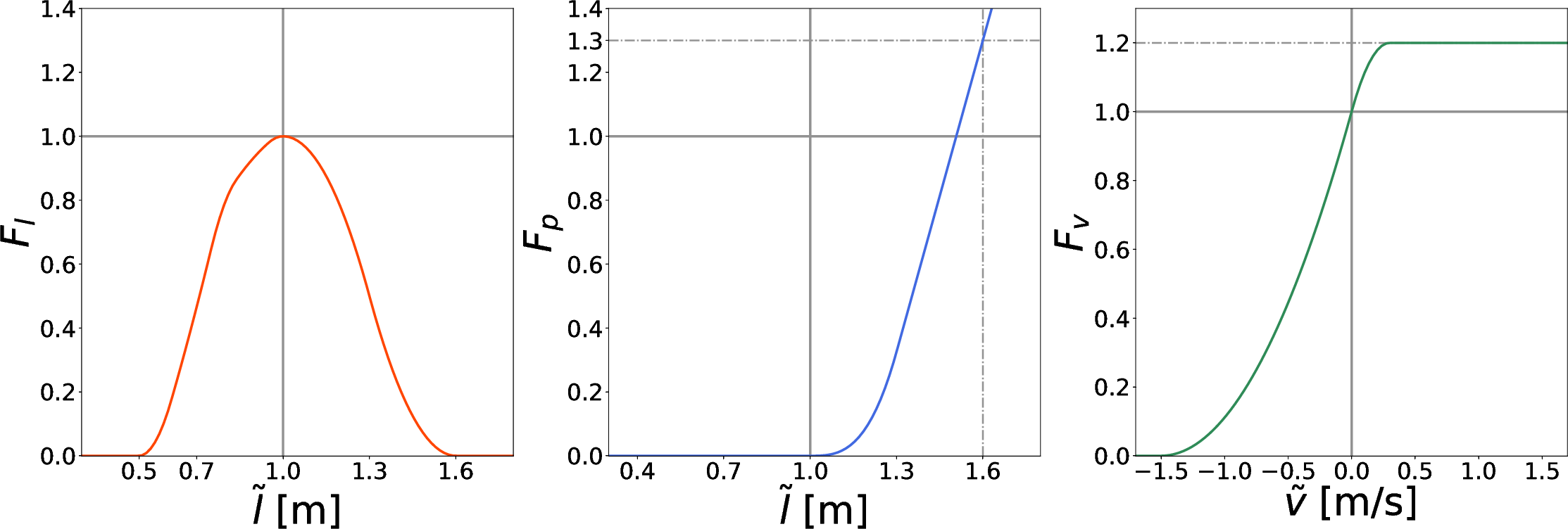

Supplement: S1 Appendix — (ZIP) [file pcbi.1011771.s001.zip › S2_Fig.tif]

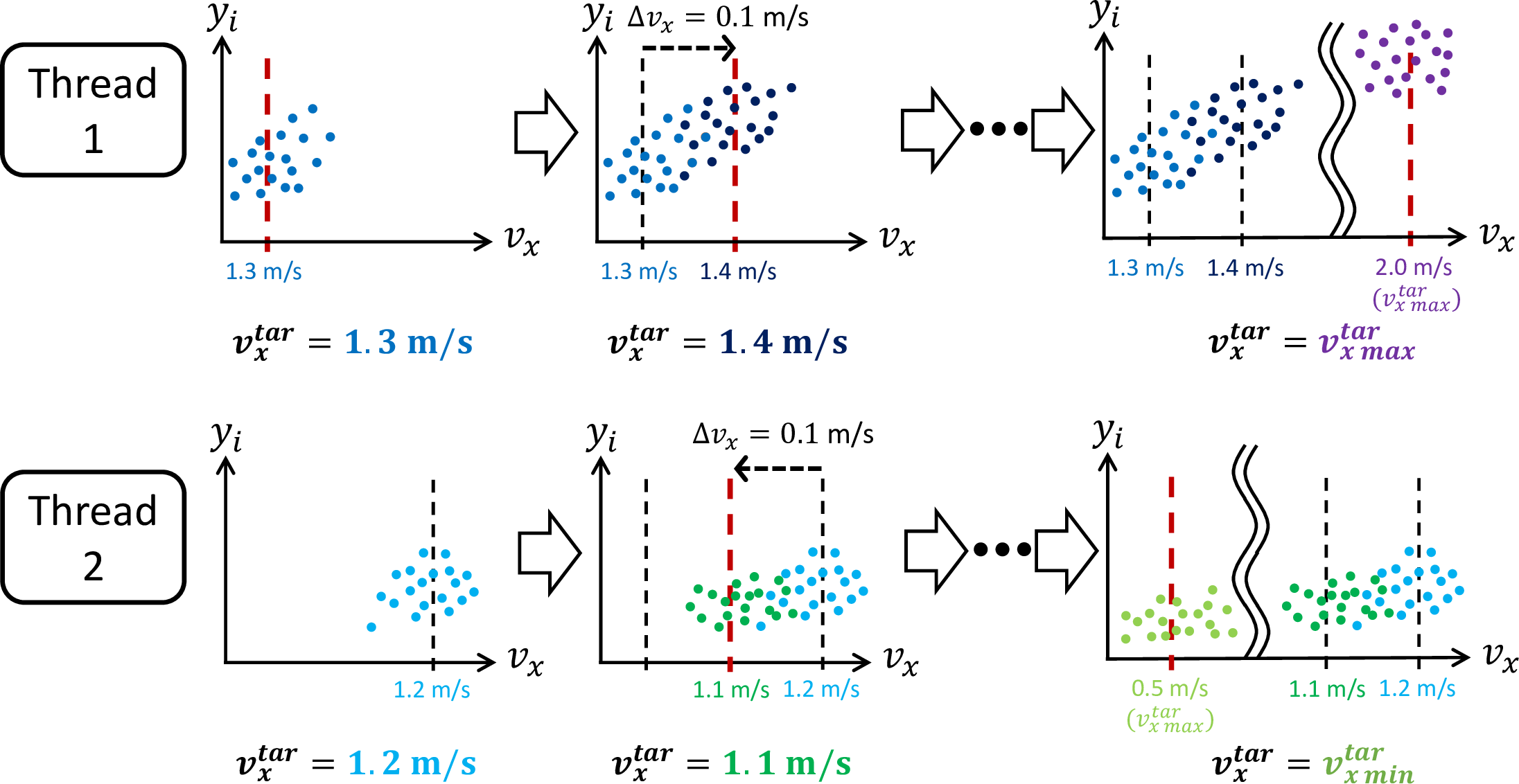

Supplement: S1 Appendix — (ZIP) [file pcbi.1011771.s001.zip › S3_Fig.tif]

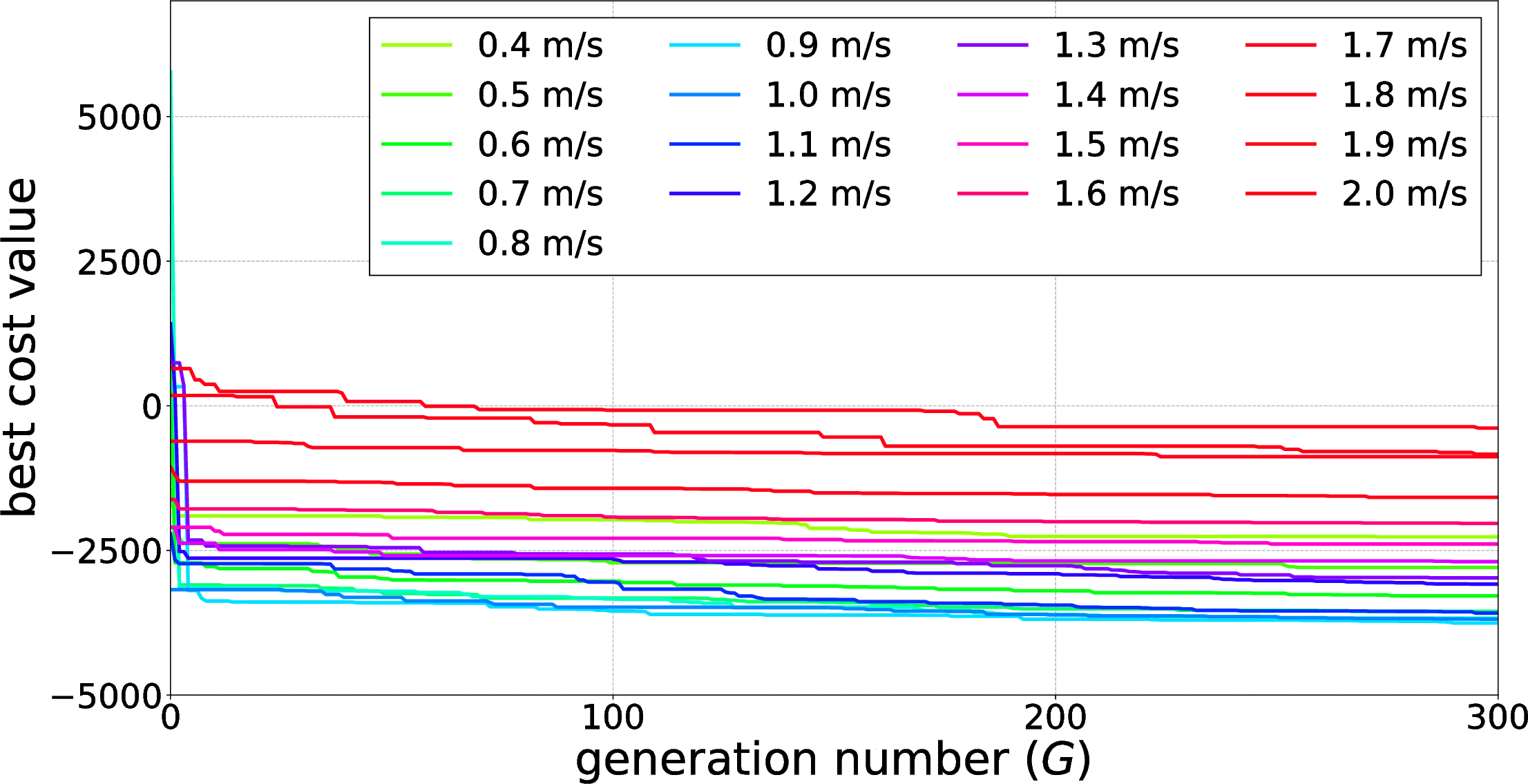

Supplement: S1 Appendix — (ZIP) [file pcbi.1011771.s001.zip › S4_Fig.tif]

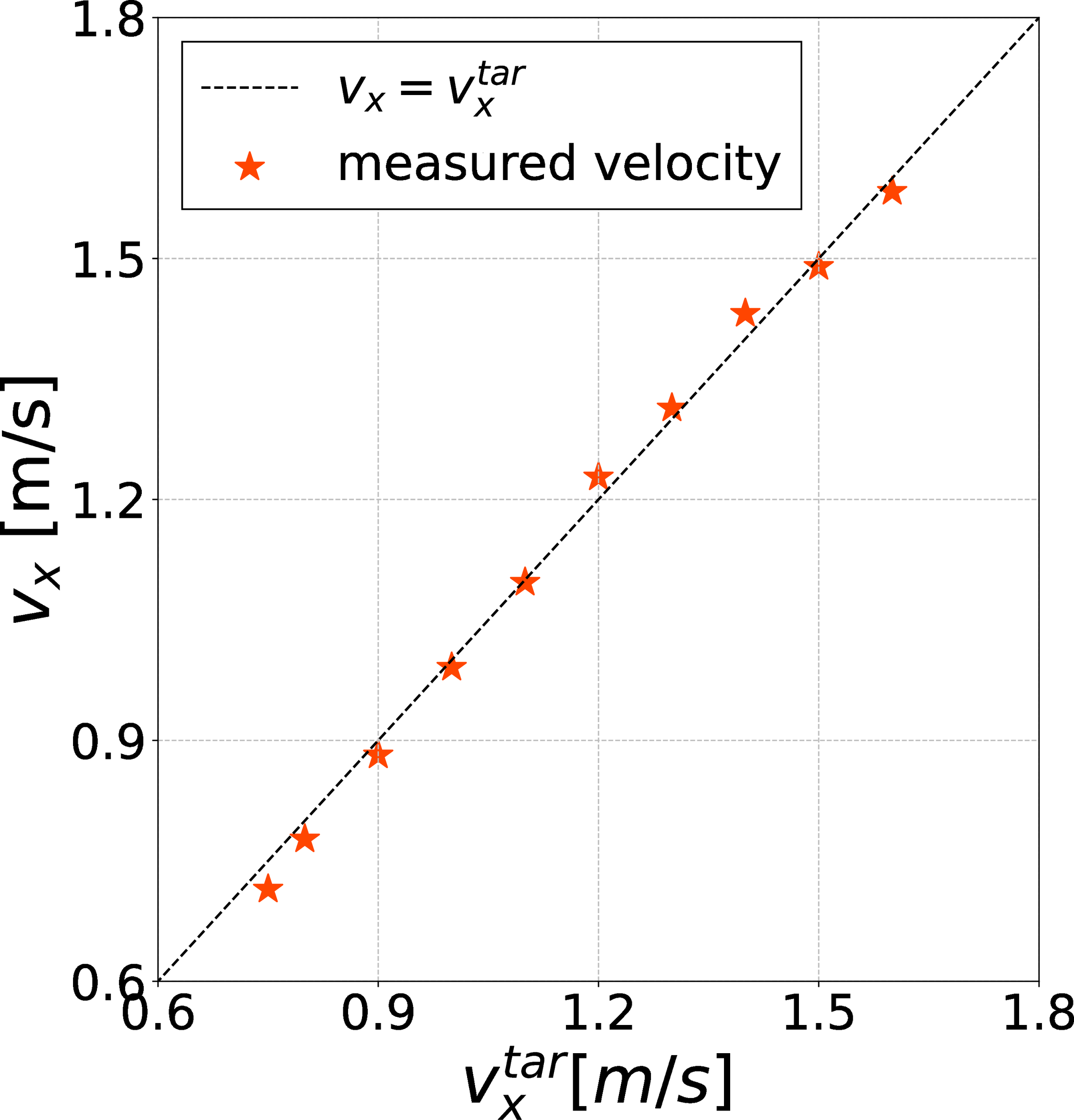

Supplement: S1 Appendix — (ZIP) [file pcbi.1011771.s001.zip › S5_Fig.tif]

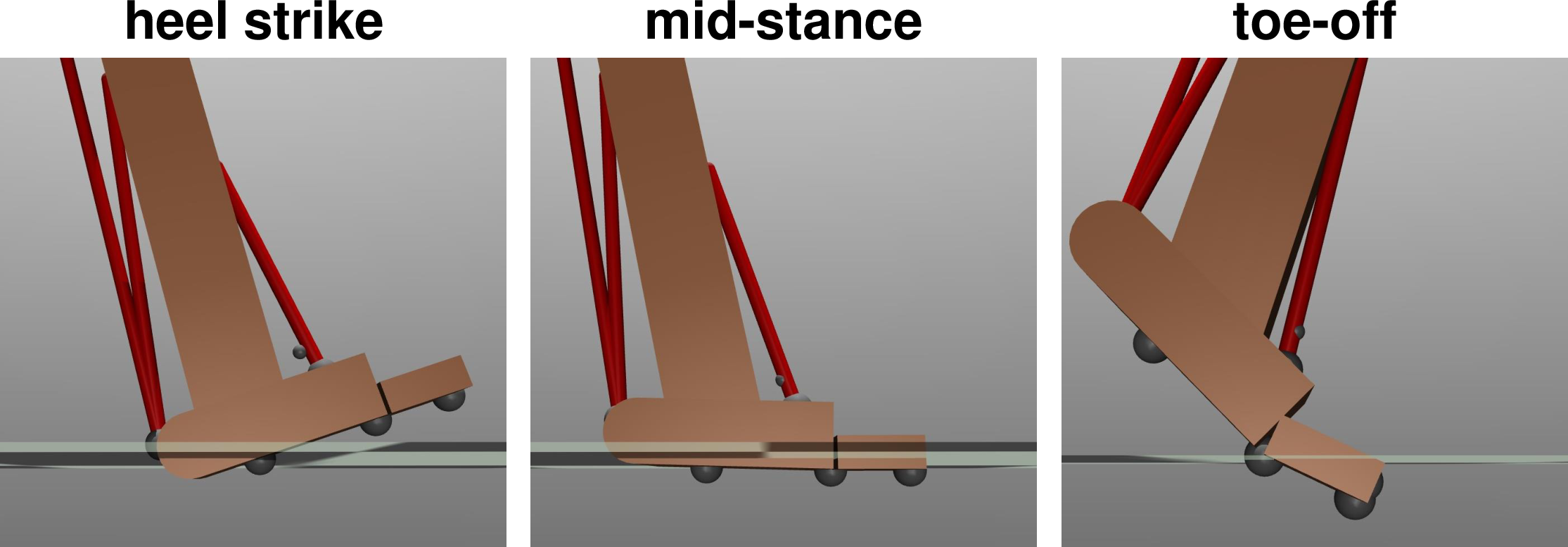

Supplement: S1 Appendix — (ZIP) [file pcbi.1011771.s001.zip › S6_Fig.tif]

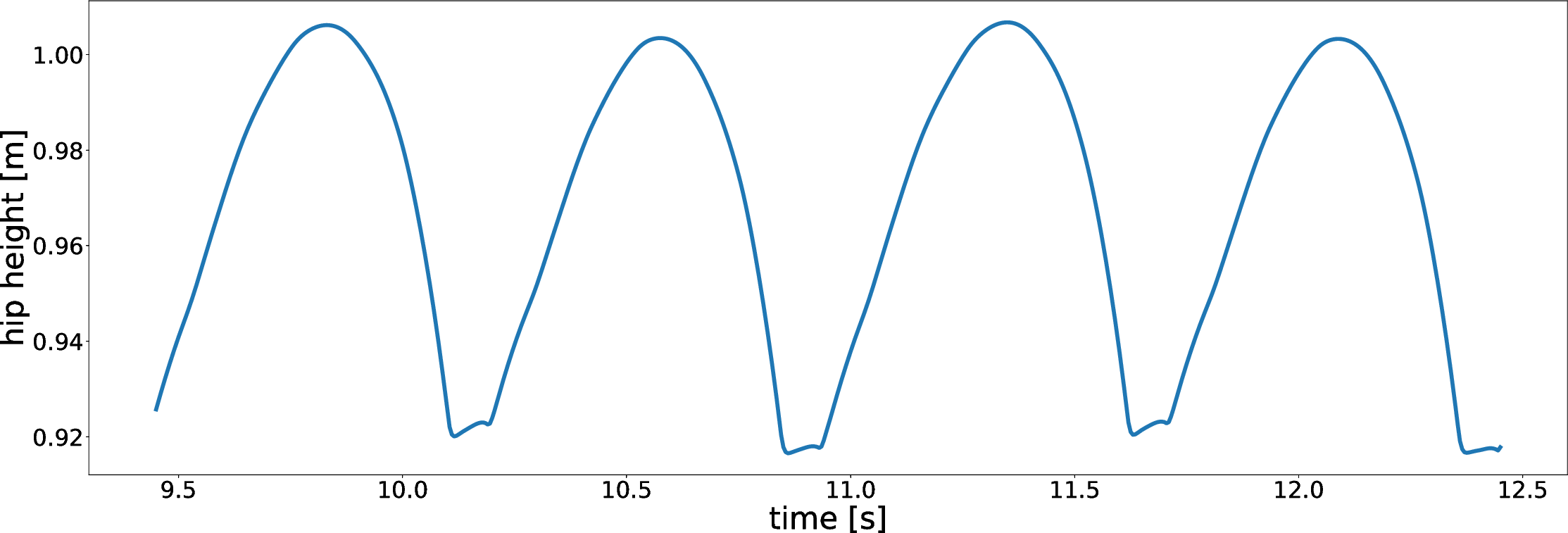

Supplement: S1 Appendix — (ZIP) [file pcbi.1011771.s001.zip › S7_Fig.tif]
