# Supplementary material for: Identifying essential factors for energy-efficient walking control across a wide range of velocities in reflex-based musculoskeletal systems
Source: PLoS Comput Biol. 2024 Jan 19;20(1):e1011771. doi: 10.1371/journal.pcbi.1011771 (PMC10798509; doi:10.1371/journal.pcbi.1011771)
Supplement: Attachment — Submitted filename: response_to_reviewers.pdf [file pcbi.1011771.s003.pdf]

September 15th, 2023

Cover Letter Describing Our Responses to Editorial Decision (PCOMPBIOL-D-23-00582) from PLOS Computational Biology

Dear Associate Professor James O'Dwyer and Reviewers:

We extend our heartfelt gratitude for considering our manuscript (PCOMPBIOL-D-23-00582) for publication in your esteemed journal *PLOS Computational Biology.* We are also appreciative of the opportunity to engage in a constructive discussion with the reviewers.

The insightful comments provided by the reviewers have been instrumental in enhancing the quality of this manuscript. Please find below our point-by-point responses to the issues raised by the reviewers. We hope that the revised version of the manuscript meets standards for publication in your journal.

We are grateful once again for your time and consideration. We look forward to hearing from you.

Sincerely,
Shunsuke Koseki

Dept. of Robotics, Graduate School of Engineering,
Tohoku University
6-6-01 Aoba, Aramaki, Aoba-ku, Sendai 980-8579, Japan
E-mail address: shunsuke.koseki.q4[at]dc.tohoku.ac.jp

Title: Identifying essential factors for energy-efficient walking control across a wide range of velocities in reflex-based musculoskeletal systems

Authors: Shunsuke Koseki[1], Mitsuhiro Hayashibe[1], and Dai Owaki[1]

Affiliations:

[1] Department of Robotics, Graduate School of Engineering, Tohoku University, Sendai 980-8579, Japan.

Description of the modifications in the revised manscript

We are grateful to the reviewers for their insightful comments, which have helped us to substantially improve this manuscript. The major aspects of our revisions to the article are summarized as follows:

(1) Clarifying the scientific problems addressed in this study,

(2) Summarizing the relationship with existing studies on the reflex-based bipedal model and identifying the contribution of this study (Fig 3 in the revised paper: overall system diagram and contributions within the system), and

(3) Clarifying the contribution of our findings to the field of biology, especially, human biomechanics.

Through the additional experiments, we have unequivocally demonstrated our conclusions and contributions to the field of human biomechanics. Specifically, we establish the criticality of "minimizing stimulation to the hip flexor (HFL) during the swing phase, while ensuring sufficient hip swing to prevent falls. This is achieved through the coordinated activity between the HFL and the hamstrings (HAM) during the swing phase, which is a crucial aspect for maintaining energy efficiency in reflex-based walking control across a wide range of velocities." Furthermore, we have meticulously validated the robustness and reliability of these findings, independent of the parameters used, i.e., leg length, sensory time delay, and weight coefficients in the objective cost function.

During the modification process, we implemented certain adjustments to both the musculoskeletal model and the range of the control parameters. These modifications substantially contributed to the attainment of more reliable and evident conclusions.

- The range of control variable values, specifically, the range of values of p_i in Eq.(S1)-(S9) and q_i in Eq.(S14)-(S22), differed significantly from those used in the previous studies.
- We have corrected the wrong sign in Eq.(S26) from $\theta_h^{tar} = \theta_h^{tar} - c_d d + c_v v_x$ to $\theta_h^{tar} = \theta_h^{tar} + c_d d + c_v v_x$.
- The inclusion of the ankle segment of the model employed in the previous manuscript was deemed unnecessary, as it artificially increased the mass around the ankle. Consequently, we have updated the musculoskeletal model.

Finally, to better incorporate the contributions obtained in this study into the title of this manuscript, we have changed the title as follows.

Title: Identifying essential factors for energy-efficient walking control across a wide range of velocities in reflex-based musculoskeletal systems

Please find below our point-by-point responses to the issues raised by the reviewers. We hope that the revised manuscript is now suitable for publication in your journal.

Please click the following hyperlink to jump to each response.

Reviewer #1

Reviewer #2

Reviewer #3

Comments from Reviewer #1

Red font: Reviewer's comments
Black font: Authors' comments
Blue font: Revised or newly added sentences in the revised paper

Comment 1

While acknowledging the importance of neuro-muscular feedback the authors make numerous changes which deviate substantially from a reflexive controller without clearly motivating them. CPGs, which are prominently featured in the introduction do not seem to play a role in the presented approach. The introduction of desired positions, constant control parameters and PD-control for selected muscles highly increases the number of control parameters. The motivation and influence these changes have and their individual impact is not explained.

Response to comment 1:

We express our gratitude to the reviewer for the careful review of our manuscript. The reviewer's comments were exceptionally insightful and provided us with a more nuanced perspective of our study. The reflex-based controller employed in this study is identical in principle to that of Wang et al (Wang et al., 2012). We made no ad-hoc adjustments to the basic reflex-based control model to ensure optimal simulation performance. In the revised manuscript, because the reflex-based controller remains identical to that used in the previous work, we have described the overview of the controller in the main manuscript and have explained the detailed equations in the Supplementary Material. This approach was undertaken as suggested by the second reviewer (Reviewer #2, Minor Comment 2) to clarify and highlight the novel contribution of this study. The novel contribution is to extend the reflex-based control system to include speed control and identify key reflex circuits that contribute to energy-efficient walking across wide-range velocities. Therefore, we firmly hope that this revision affirms our contributions. The impacts of introducing PD control and desired position control (SIMBICON) have been explained in the manuscript and Supplementary Material, respectively, based on the description in the original paper as follows:

Generating walking through reflex-based control: Reflex-based control, Lines 113-149

The reflex-based controller employed in this study is identical in principle to that of Wang et al. [28]. We did not make any ad-hoc adjustments to the basic reflex-based control model to ensure optimal simulation performance. The controller computes the muscle stimulation, denoted as u_i, for each muscle, i, by incorporating sensory feedback with a time delay of Δt as input. The control law switches depending on whether the leg is in the stance or swing phase. Moreover, additional stimulation is introduced during the late stance and late swing phases. The control system comprises three primary functions: force feedback, length feedback, and muscle-driven proportional derivative (PD) control.

- Force feedback:
  The force feedback law returns the stimulation signal, u_i^F, in response to the actuator force, F_i. In humans, the signals of the F_i arise from Golgi tendon organs and are carried by type Ib afferents to the spinal cord. u_i^F is calculated as follows:

$$u_i^F = G_i \tilde{F}_i(t - \Delta t_i), \tag{1}$$

$$\tilde{F}_i(t - \Delta t_i) = \frac{F_i(t - \Delta t_i)}{F_i^0}, \tag{2}$$

  where the gain, G_i, is the positive control parameter, and $\tilde{F}_i(t - \Delta t_i)$ is the scaled actuator force, (i.e., $\tilde{F}_i = F_i/F_i^0$), which includes the sensory time delay, Δt_i.

- Length feedback
  Through length feedback, we calculate the stimulation signal, denoted as u_i^l, corresponding to the length of the muscle actuator, l_i. This function models the stretch reflex of the muscle spindle. u_i^l is calculated as follows:

$$u_i^l = \max\{0, G_i(l_i(t - \Delta t_i) - l_i^{tar})\}, \tag{3}$$

  where the target length, l_i^{tar}, and gain, G_i, are positive control parameters.

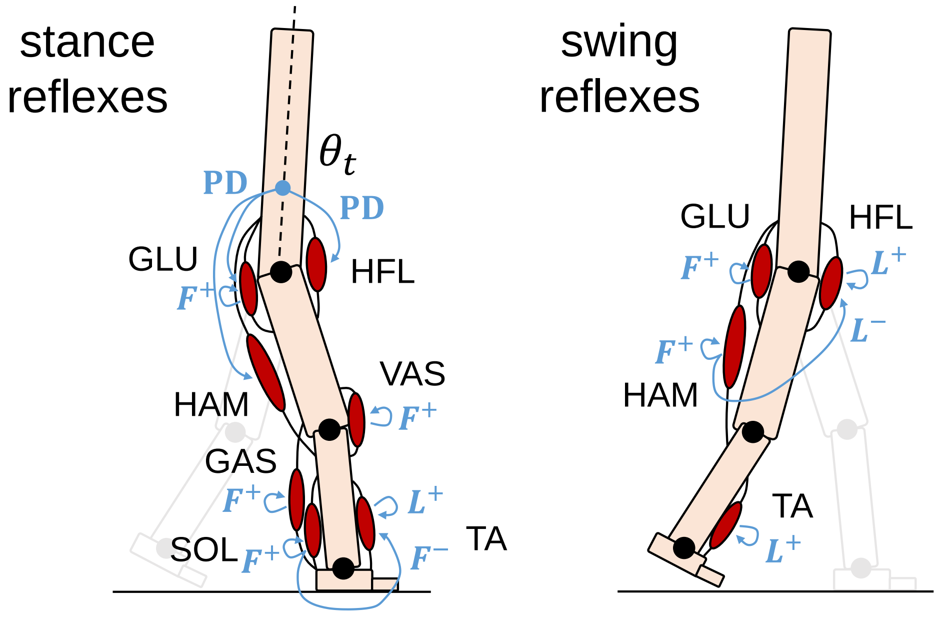

Fig 2. Key reflexes in the stance and swing phase. F represents force feedback, L represents length feedback, and PD represents muscle-driven PD control. $+$ and $-$ denote positive and negative feedback, respectively. In the stance phase, F^+ at GLU, VAS, and SOL generate compliant leg behavior. L^+ at TA prevents overextension of the ankle joint, which is suppressed by the F^- from the SOL. F^+ at GAS contributes to push-off and prevents overextension of the knee joint. Muscle-driven PD controls at HFL, GLU, and HAM balance the torso. In the swing phase, L^+ facilitates leg swing, which is suppressed by L^- from HAM. F^+ at GLU and HAM apply braking force to the swing leg. L^+ at TA raises the toes to create clearance between the feet and the ground.

- Muscle-driven PD control
  The muscle-driven PD control generates the stimulation necessary to move joint θ_j (Fig 1B) to the target angle, θ_j^{tar}. This function can be interpreted as a polysynaptic reflex that is mediated by the joint position afferent input from group III fibers and descending signals of the target joint angles from the supraspinal. The muscle-driven PD control laws are employed to stabilize the generated gait by the hip muscles during the stance phase to balance the torso and during stance preparation to prepare for ground contact (described in S1 Appendix). For muscle actuator, i, which applies torque to joint θ_j in the positive direction, $u_i^{\theta_j}$ is defined as follows:

$$u_i^{\theta_j} = \max\{0, K_i(\theta_j^{tar} - \theta_j(t - \Delta t_i)) - D_i\dot{\theta}_j(t - \Delta t_i)\}. \quad (4)$$

  Conversely, for muscle actuator i, which applies torque in the negative direction, u_i^{θ} is defined as follows:

$$u_i^{\theta} = \max\{0, K_i(\theta_j(t - \Delta t_i) - \theta_j^{tar}) + D_i\dot{\theta}_j(t - \Delta t_i)\}, \quad (5)$$

  where the proportional gain, K_i, and derivative gain, D_i, are positive control parameters.

Fig 2 illustrates the reflexes in the stance phase and swing phase. Please see S1 Appendix or the original paper [28] for more details.

Modulation of control parameters, Lines 152-162

Fig 3 displays the control diagram of the musculoskeletal model, which is structured into spinal and supraspinal layers. We added a parameter modulator that allows a wide range of walking while maintaining energy efficiency to the previous model [28]. The reflex-based controller in the spinal layer activates muscles. The parameter modulator in the supraspinal layer coordinates the control parameter set $\boldsymbol{Y}^{tar}$ to achieve the input target velocity, v_{vel}^{tar}. Each control parameter, $y_i^{tar} \in \boldsymbol{Y}^{tar}$, is calculated using the function $P_i(v_x)$ in the parameter modulator for the input target velocity, v_x^{tar} (Fig 4). $P_i(v_x)$ is derived via the polynomial regression of the relationship between the velocity and parameter value, which is determined through optimization by increasing/decreasing the target velocity stepwise. SIMBICON in the supraspinal cord adjusts the desired foot placements.

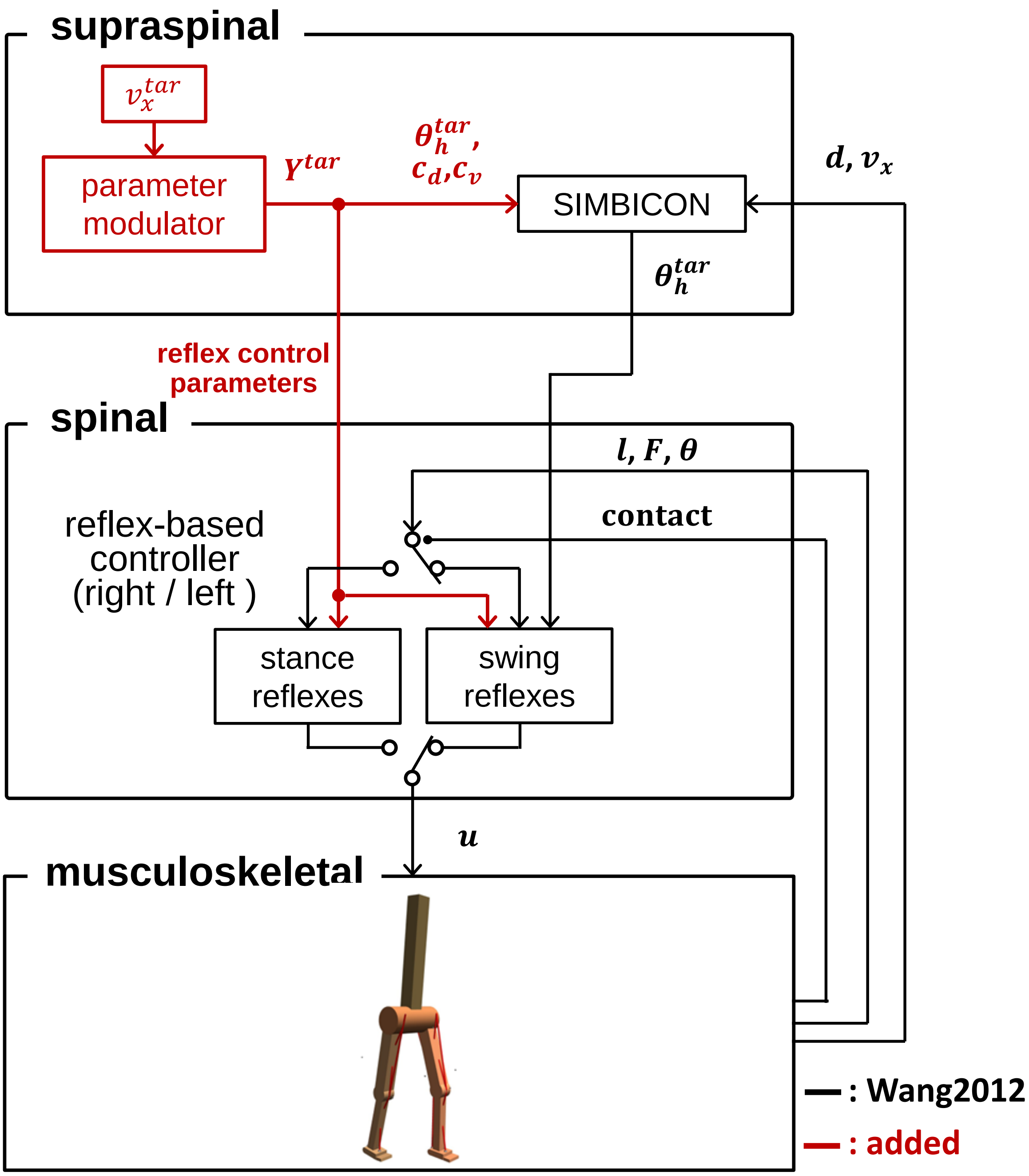

Fig 3. Control diagram for the musculoskeletal model.
The musculoskeletal model is driven by activated muscles receiving stimulation signals, u, from the reflex-based controller in the spinal layer. Each leg (right and left) is controlled by separate stance and swing reflexes. The control law switches depending on whether the leg is in contact with the ground. Sensory information from the model is fed back to the controller for motion generation. The supraspinal layer controls the walking velocity and adjusts the desired foot placements. The parameter modulator coordinates the control parameter set $\boldsymbol{Y}^{tar}$ to achieve input target velocity, v_{vel}^{tar}. SIMBICON adjusts the target hip angle, θ_h^{tar}, in the stance preparation phase using the sensory information (d and v_x, see S1 Appendix.

Supplementary material: Reflex-based control, Lines 8-38

Stance Phase

During the stance phase, the stimulation, u_i, to each muscle actuator is calculated as follows:

$$
\begin{aligned}
u_{SOL} &= p_{SOL} + G_{SOL}\tilde{F}_{SOL}(t-\Delta t_l), && (6)\\
u_{TA} &= p_{TA} + \max\{0, G_{TA\_st}(l_{TA}(t-\Delta t_l) - l^{tar}_{TA\_st})\} - G_{SOL\_TA}\tilde{F}_{SOL}(t-\Delta t_l), && (7)\\
u_{GAS} &= p_{GAS} + G_{GAS}\tilde{F}_{GAS}(t-\Delta t_l), && (8)\\
u_{VAS} &= p_{VAS} + G_{VAS}\tilde{F}_{VAS}(t-\Delta t_m) + f_{VAS}, && (9)\\
f_{VAS} &= \begin{cases} 0 & (\theta_k < \theta_k^{off}) \\ k_{\theta_k}\{\theta_k(t-\Delta t_{VAS}) - \theta_k^{off}\} & (\theta_k \geq \theta_k^{off}) \end{cases}, && (10)\\
u_{HAM} &= p_{HAM} + \max\{0, K_{HAM}(\theta_t(t-\Delta t_s) - \theta_t^{tar}) + D_{HAM}\dot{\theta}_t\}, && (11)\\
u_{RF} &= p_{RF}, && (12)\\
u_{GLU} &= p_{GLU} + \max\{0, K_{GLU}(\theta_t(t-\Delta t_s) - \theta_t^{tar}) + D_{GLU}\dot{\theta}_t\}, && (13)\\
u_{HFL} &= p_{HFL} + \max\{0, K_{HFL}(\theta_t^{tar} - \theta_t(t-\Delta t_s)) + D_{HFL}\dot{\theta}_t\}, && (14)
\end{aligned}
$$

where p_i are positive constant control parameters that are approximate to zero. The time delays, Δt_l, Δt_m, and Δt_s, are set to 20, 10, and 5 ms, respectively. The shorter transmission pathway to the spinal cord results in smaller time delays; therefore, the muscles near the hip joint have minimal time delays. Positive force feedback at GLU, VAS, and SOL contributes to compliant leg behavior. When the gains, denoted as G, are set higher, the stance leg emulates an inverted pendulum model. Conversely, when the gains are set at smaller values, it behaves as a spring-inverted pendulum model [3]. Positive length feedback at TA prevents overextension of the ankle joint. This action is suppressed by negative force feedback from the SOL, which serves to extend the ankle joint during a push-off. Positive force feedback at GAS contributes to push-off and prevents overextension of the knee joint. Muscles around the hip joint, namely HFL, GLU, and HAM, function together to maintain the reference lean angle θ_t^{tar} through muscle-driven PD controls, thus stabilizing the torso. The term, f_{VAS}, prevents knee hyperextensions during the mid-stance phase. Stimulation of the VAS is suppressed when the knee joint angle, θ_k, exceeds the threshold angle, θ_k^{off}. During the swing initiation phase, when the hip extension angle, θ_h, exceeds θ_{SI}, as depicted in Fig S1A, constant stimulation values s_i that are greater than zero are added to the VAS, RF, GLU, and HFL to encourage swing initiation [4].

$$
\begin{aligned}
u_{VAS} &\leftarrow u_{VAS} - s_{VAS}, && (15)\\
u_{RF} &\leftarrow u_{RF} + s_{RF}, && (16)\\
u_{GLU} &\leftarrow u_{GLU} - s_{GLU}, && (17)\\
u_{HFL} &\leftarrow u_{HFL} + s_{HFL}. && (18)
\end{aligned}
$$

Swing Phase

During the swing phase, the stimulation, u_i, to each muscle actuator is calculated as follows:

$$
\begin{aligned}
u_{SOL} &= q_{SOL}, && (19)\\
u_{TA} &= q_{TA} + \max\{0, G_{TA\_sw}(l_{TA}(t-\Delta t_l) - l^{tar}_{TA\_sw})\}, && (20)\\
u_{GAS} &= q_{GAS}, && (21)\\
u_{VAS} &= q_{VAS}, && (22)\\
u_{HAM} &= q_{HAM} + G_{HAM}\tilde{F}_{HAM}(t-\Delta t_s), && (23)\\
u_{RF} &= q_{RF}, && (24)\\
u_{GLU} &= q_{GLU} + G_{GLU}\tilde{F}_{GLU}(t-\Delta t_s), && (25)\\
u_{HFL} &= q_{HFL} + \max\{0, G_{HFL}(l_{HFL}(t-\Delta t_s) - l^{tar}_{HFL})\} \\
&\quad - \max\{0, G_{HAM\_HFL}(l_{HAM}(t-\Delta t_s) - l^{tar}_{HAM})\} + f_{HFL}, && (26)\\
f_{HFL} &= k_{lean}\{\theta_t^{tar} - \theta_t(t-\Delta t_{HFL})\}, && (27)
\end{aligned}
$$

where q_i are positive constant control parameters similar to p_i and also approximate zero. Positive length feedback at HFL facilitates leg swing, which is suppressed by negative length feedback from HAM in the mid-swing phase. The term f_{HFL} stimulates the HFL according to the current torso angle, θ_t, and the torso target angle, θ_t^{tar}. The required protraction speed relies on the torso lean. Positive force feedback at GLU and HAM applies braking force to the swinging leg, transferring some of the angular momentum into leg lowering and retraction. Positive length feedback at TA raises the toes, ensuring sufficient clearance between the feet and the ground. During the stance preparation phase, when the hip flexion angle, θ_h, exceeds θ_{SP}, as depicted in Fig S1A, the swinging leg transitions into stance preparation. Muscle-driven PD controls are integrated into the VAS, GLU, and HFL to produce the desired pose for touchdown in this phase.

$$\begin{aligned} u_{VAS} &\leftarrow u_{VAS} \\ &\quad + \max\{0, K_{SP\_VAS}(\theta_k(t-\Delta t_m) - \theta_k^{tar}) + D_{SP\_HFL}\dot{\theta}_k(t-\Delta t_m)\}, \quad (28) \\ u_{GLU} &\leftarrow u_{GLU} \\ &\quad + \max\{0, K_{SP\_GLU}(\theta_h^{tar} - \theta_h(t-\Delta t_m)) + D_{SP\_GLU}\dot{\theta}_h(t-\Delta t_m)\}, \quad (29) \\ u_{HFL} &\leftarrow u_{HFL} \\ &\quad + \max\{0, K_{SP\_HFL}(\theta_h(t-\Delta t_m) - \theta_h^{tar}) + D_{SP\_HFL}\dot{\theta}_h(t-\Delta t_m)\}. \quad (30) \end{aligned}$$

To reproduce a stable gait, the target hip angle, θ_h^{tar}, is adjusted according to the balance feedback law (SIMBICON) [5]:

$$\theta_h^{tar} = \theta_h^{tar} - c_d d + c_v v_x, \quad (31)$$

where d represents the horizontal distance from the stance ankle to the hip segment, as depicted in Fig S1B. v_x represents the horizontal velocity of the model, and c_d and c_v are positive constant parameters. This balance feedback law can be interpreted as the supraspinal control that uses sensory information from the vestibular organs.

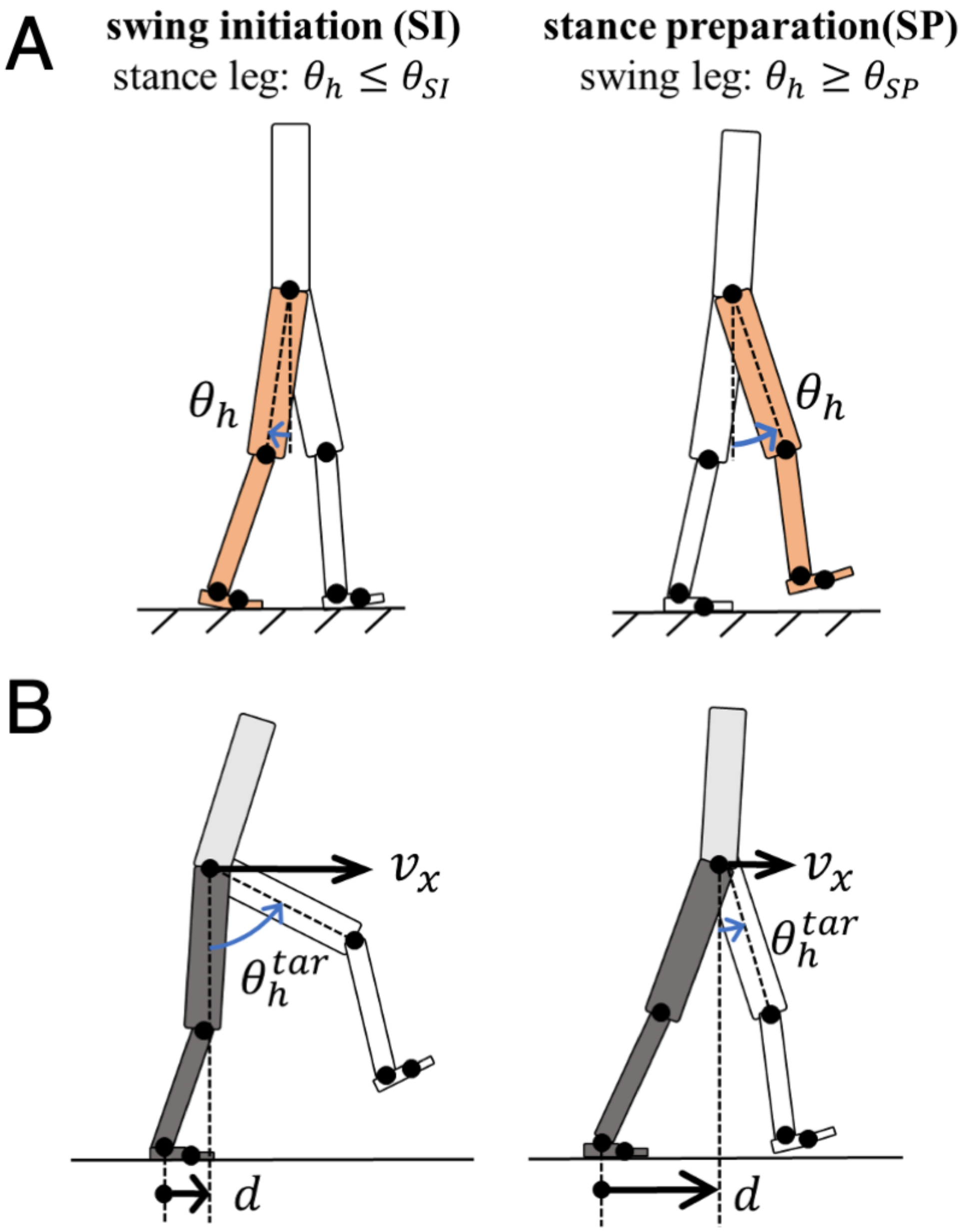

Fig S1: Description of the reflex-based control. A. Transition conditions of swing initiation (SI) and stance preparation (SP) phases. The SI phase commences when the hip extension angle, θ_h, of the stance leg exceeds a predetermined threshold, θ_{SI}. The SP phase commences when the hip flexion angle, θ_h, of the swing leg exceeds the threshold, θ_{SP}. **B. Diagram of the balance feedback law.** The feedback control system dynamically adjusts the target hip angle, θ_h^{tar}, depending on the horizontal distance from the stance ankle to the hip segment, d, and the horizontal model velocity, v_x. For instance, when d is small or v_x is large, θ_h^{tar} is increased, causing the foot to be placed more forward to prevent falling, as depicted on the left. Conversely, when d is large or v_x is small, θ_h^{tar} is decreased.

Comment 2

The authors have implemented their model in Mujoco but it is not clear to the reviewer, if the simulation generates physically feasible results. At least, examples of ground reaction forces should be shown to demonstrate a sound simulation.

Response to Comment 2:

We are grateful for your insightful comment. We employed MujoCo because of its capability in conducting simulations with relatively low computational costs while ensuring the validity of physical calculations. The collision model integrated into MujoCo accurately models contact and repulsive forces between objects with complex shapes, accounting for their shape, size, and material properties. The algorithm incorporates the flexibility to achieve precise simulation of contact and reaction forces. Considering one of the core aspects of this study, –optimizing 56 parameters to replicate efficient gait across a wide range of speeds——, we considered it impractical to use OpenSim. While OpenSim is a reliable tool in the field of biomechanics, considering the computational time costs involved, it is not a feasible option.

To validate the physics of the simulation, we have measured the ground reaction forces (GRFs) of the generated gaits at slow, normal, and fast walking speeds and compared them to those of human data. Although we observed overshoots and undershoots of GRFs at heel and toe strikes, the cross-correlation values (R) between the GRFs of the generated and human gaits were positive in fore-art (x) and vertical directions (z) for all conditions.

We have incorporated the rationale behind using MujoCo simulations and the validation of GRFs in the following part of the revised manuscript.

Generating walking through reflex-based control: Musculoskeletal model, Lines 67-75

A two-dimensional musculoskeletal model with a height of 180 cm and weight of 80 kg was employed in this study, as illustrated in Fig 1A. Detailed parameters are listed in Table 1. The model was constructed within a MuJoCo simulation environment [42]. We employed Mujoco because of its ability to conduct simulations at a relatively low computational cost while ensuring the validity of the physical calculations. The collision model implemented in Mujoco accurately models contact and repulsive forces between objects with complex shapes, considering their shape, size, and material properties. This algorithm incorporates the flexibility required for a precise simulation of contact and reaction forces. ...

Results: Generated gaits, Lines 318-320

... Fig 8 illustrates the ground reaction forces (GRF) and kinematics of the generated gait using the optimized functions derived with $A = 10^6$ and those of humans at corresponding speeds [57].

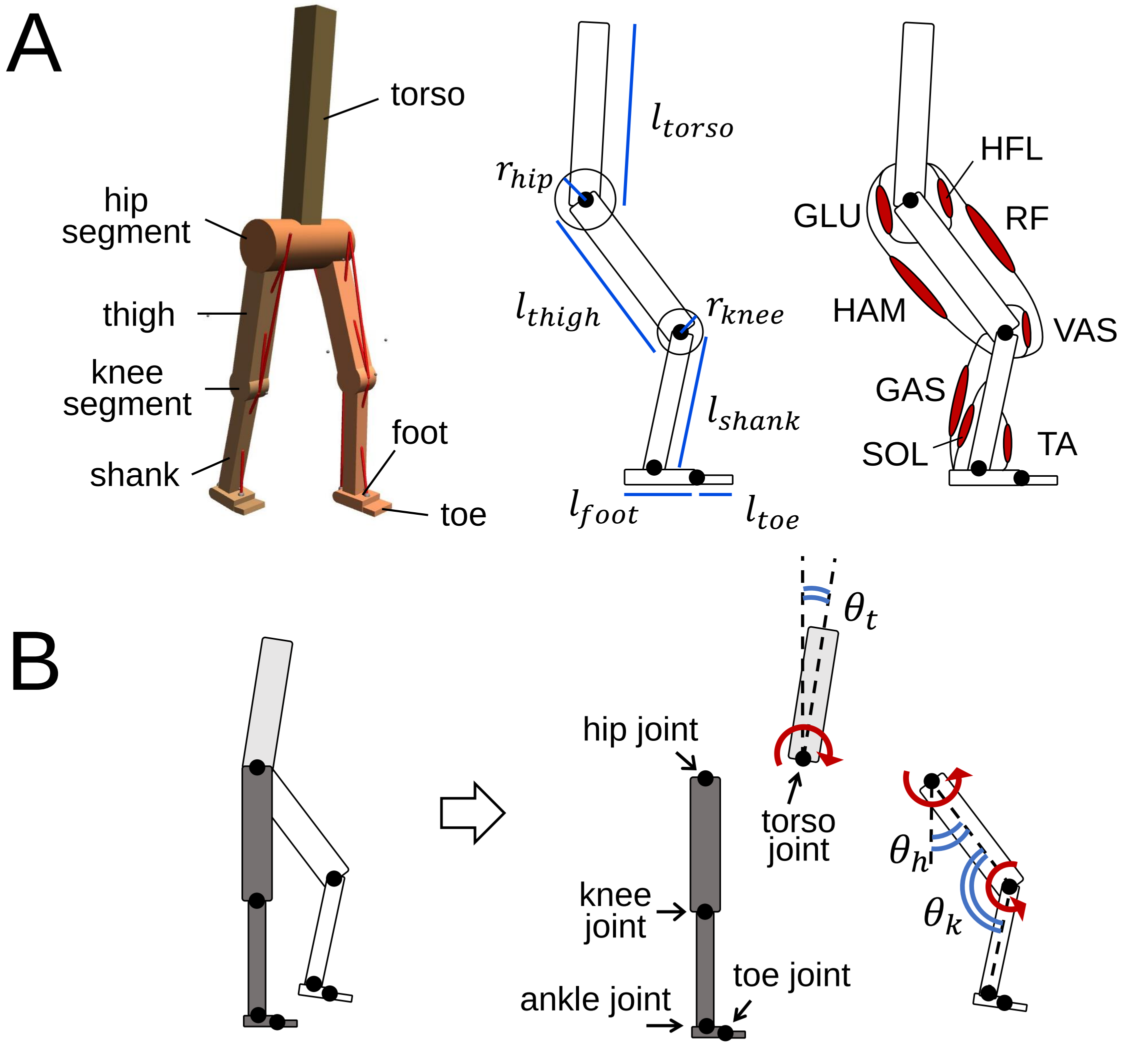

Fig 1. Musculoskeletal model employed in this study. A. Structure of the musculoskeletal model. Left: Oblique view of the bipedal model in the MuJoCo simulator. Center: Side view of the model with segment parameters. Right: Muscle alignments. **B. Internal degrees of freedom of the model.** θ_t, θ_h, and θ_k represent torso joint angle, hip joint angle, and knee joint angle, respectively. The red arrows depict the positive direction of joint rotation.

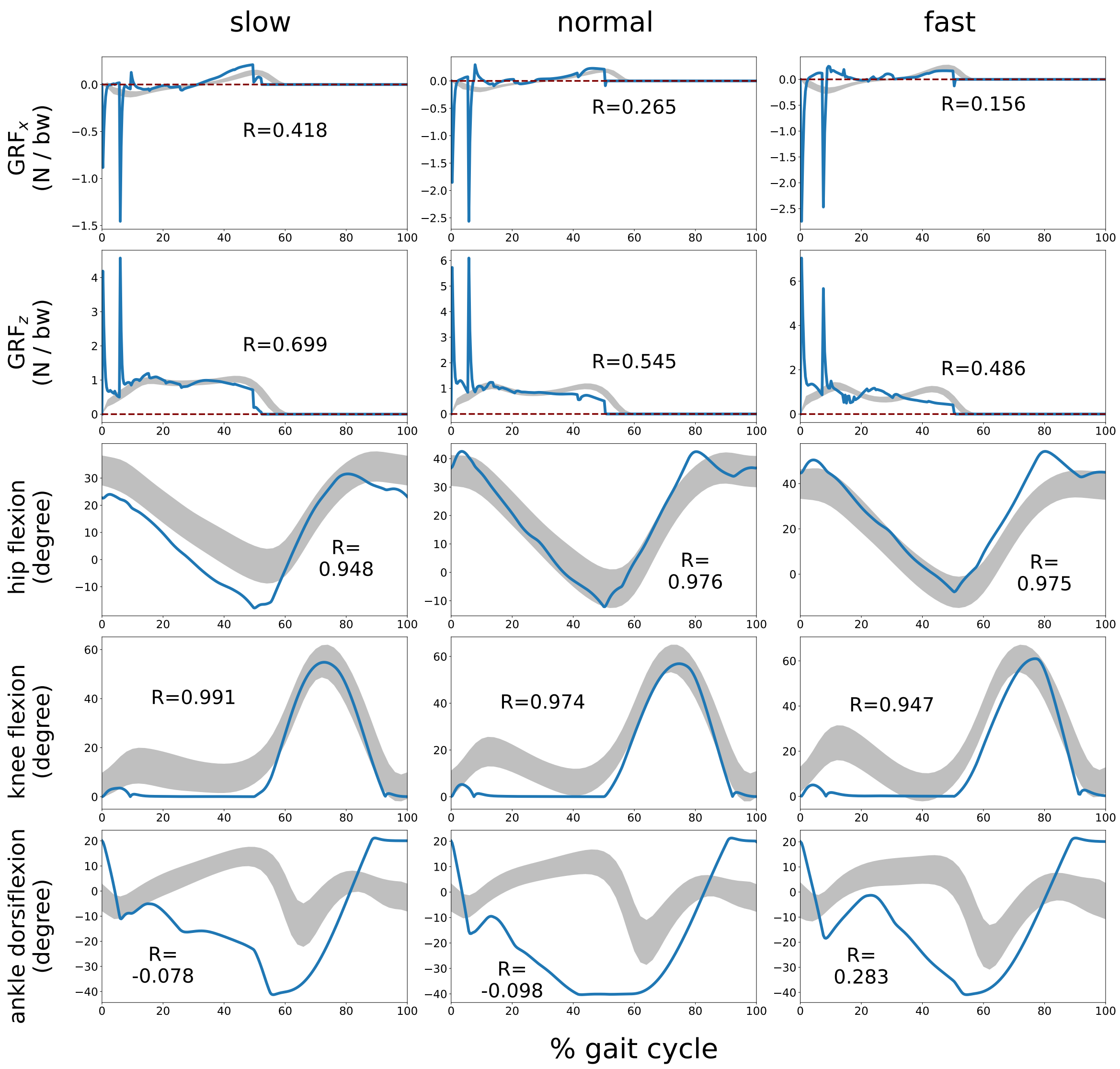

Fig 8. GRFs and kinematics of the generated gait. v_x^{tar} was set to 0.9 (slow), 1.25 (normal), and 1.6 m/s (fast). Blue lines depict the generated gait, while gray lines represent those of humans (mean±1 s.d.) [57]. GRF values are normalized by the body weight. R denotes the cross-correlation value.

Comment 3

While the question is interesting, and has been to an extend already answered by Dzeladini, and the optimization approach is interesting, the reviewer does not understand the reason for not applying this optimization to the original, reflex based model and can only assume that the required adaptions are necessary to make the simulation work. This however would largely reduce the optimization's impact on general problems.

Response to Comment 3:

As carefully explained in our response to comment 1, the reflex-based controller employed in this study is identical in principle to that of Wang et al., which is based on Geyer's model. Therefore, we believe that the generality regarding the optimization is not lost. Again, we did not make any ad-hoc adjustments to the basic reflex-based control model to ensure optimal simulation performance.

Generating walking through reflex-based control: Reflex-based control, Lines 113-115

> ... The reflex-based controller employed in this study is identical in principle to that of Wang et al. [28]. We did not make any ad-hoc adjustments to the basic reflex-based control model to ensure optimal simulation performance. ...

Comment 4

According to their discussion, the authors fail to clearly motivate their extensive extension of the reflex-based control system, and also did not clearly identify relevant reflex-control parameters, as many parameters seem to have no influence and of those who have, many have been introduced by the authors without a clear functional explanation. The term reflex rules seem to be very losely used as any type of feedback. Especially the direct inclusion of joint angles into the feedback rule deviates from established proprioceptive understanding of muscle-tendon units and their control.

Response to Comment 4:

Thank you for the insightful comment and valuable input. To better emphasize the motivation of this study, we have revised **Introduction**.

Through the additional experiments (**Results: Identifying factors essential for improving energy efficiency, Key reflex circuits for energy-efficient walking over wide range velocities, and Robustness to parameter changes**), we have been able to demonstrate our conclusion and contribution to the field of human biomechanics: "minimizing stimulation to the hip flexor (HFL) during the swing phase, while ensuring sufficient hip swing to prevent falls. This is achieved through the coordinated activity between the HFL and the hamstrings (HAM) during the swing phase, which is a crucial aspect for maintaining energy efficiency in reflex-based walking control across a wide range of velocities." We have also confirmed that these facts are reliable and robust regardless of the parameters used, including leg length, sensory time delay, and weight coefficients in the objective cost function.

We incorrectly used the term "reflexes" as any kind of feedback. We have revised the manuscript to appropriately use the terms "reflex" and "feedback" to correspond to their biological meanings. We have also added biological explanations for the primary reflexive functions (**Reflex-based control**). Furthermore, we redesigned the control diagram (Fig 3) to divide it into a spinal layer and a supraspinal layer to clearly distinguish non-reflexive control.

Introduction, Lines 36-49

> Previous studies have attempted to regulate velocities within the reflex-based control frameworks [29,31], but their methods were limited to transitions between predetermined velocities and did not provide precise velocity controls. Furthermore, the transitions between these predetermined velocities were accomplished through the utilization of distinct control parameters designated for these transitions. Given that walking speed assumes continuous values, achieving precise control over walking speed through such means would theoretically require infinite parameters for transitions. Because "transition'' differs from "control'' in terms of its adaptability, it is essential to extend reflex-based systems to affect precise velocity controls while improving energy efficiency to explore reflex control mechanisms in walking. Furthermore, the development of energy-efficient control over a wide range

of velocities in the reflex-based system will lead to improving the performance of the controller for exoskeletons [38,39] and prosthetic leg [17,37] by adjusting their control parameters according to user walking velocity.

Results: Identifying factors essential for improving energy efficiency, Lines 349-363

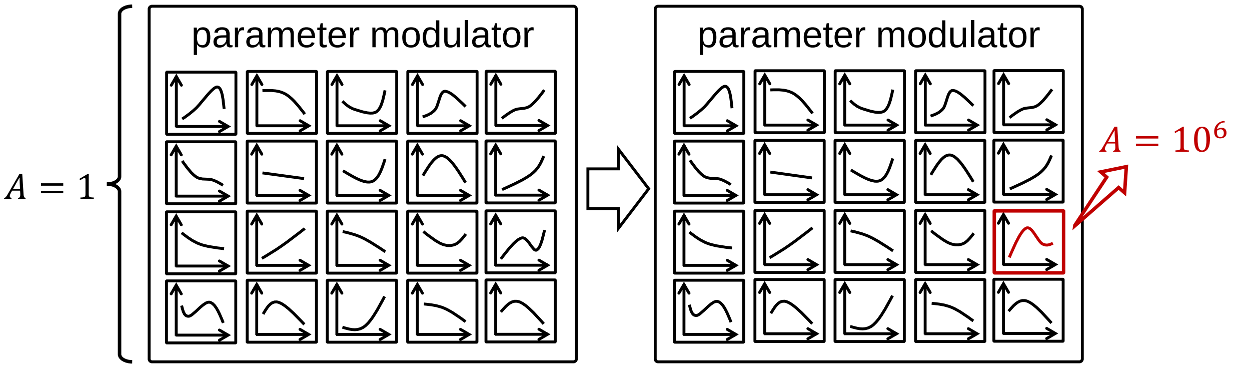

Fig 11. Illustration of the replacement of the optimized function. Left: Before the replacement, all functions were derived with $A = 1$. Right: Only the target control parameter function (red) was replaced with $A = 10^6$. Functions with other parameters were not replaced.

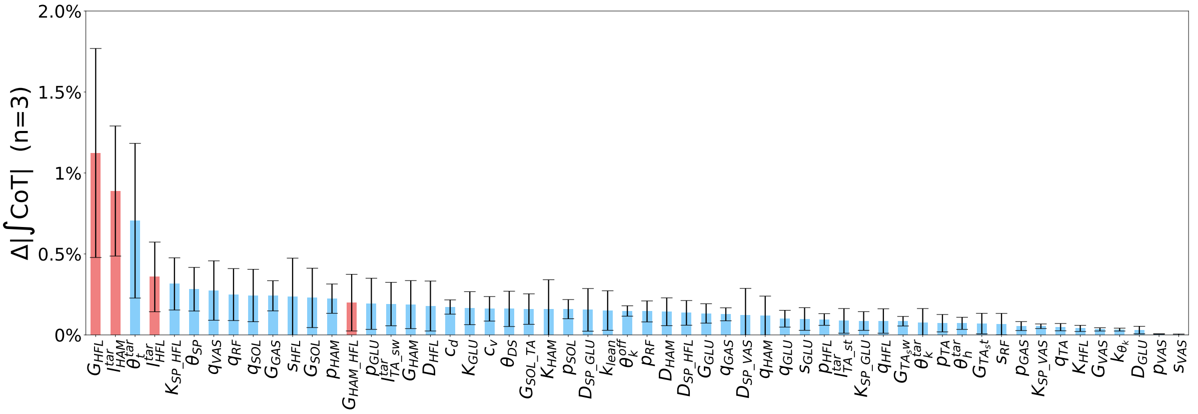

Fig 12. Absolute change in $\int$CoT values when the optimized function of each parameter was substituted to that derived with $A = 10^6$ in the generated gait with $A = 1$ ($n = 3$). The red bars represent control parameters associated with the reflex circuits under focus.

In this study, we aim to identify the key reflex circuits that influence energy-efficient walking across a wide range of velocities. First, to identify the essential factors for improved energy efficiency, we substituted the optimized function of each control parameter that derived at $A = 10^6$, which achieved more energy-efficient walking over a wide range of velocities, in the gait with $A = 1$ (a baseline). Fig 11 illustrates this approach.

Fig 12 illustrates the absolute change in percentage of the $\int$CoT values, $\Delta|\int\mathrm{CoT}|$, to the generated gait with $A = 1$. From the results, we found that G_{HFL}, l^{tar}_{HAM} and θ^{tar}_{t} are the three parameters that changed $\Delta|\int\mathrm{CoT}|$ value by more than 0.5%. This result suggests that reflex circuits that involve these parameters may have a significant impact on energy-efficient walking across a wide range of velocities. The two length-feedback reflex circuits, $G_{HFL}(l_{HFL} - l^{tar}_{HFL})$ and $G_{HAM\_HFL}(l_{HAM} - l^{tar}_{HAM\_HFL})$, is the primary circuits involved in these parameters, G_{HFL} and l^{tar}_{HAM}: both reflex circuits stimulate HFL muscle during the swing phase. θ^{tar}_{t}, which is the reference angle of the torso, changed $\Delta|\int\mathrm{CoT}|$ value of the third. This parameter was not related to a specific reflex circuit but was shared with several reflex circuits.

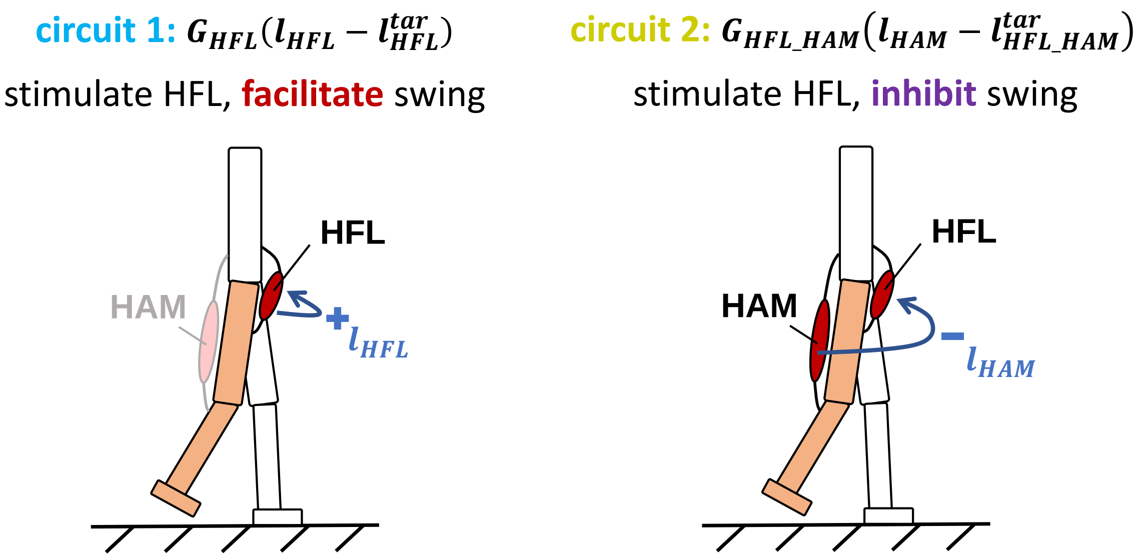

Fig 13. Key reflex circuits for energy-efficient gait. $G_{HFL}(l_{HFL} - l_{HFL}^{tar})$ is the positive length feedback stimulating HFL to swing the leg forward. $G_{HAM\_HFL}(l_{HAM} - l_{HAM\_HFL}^{tar})$ is the negative length feedback inhibiting the HFL proportional to the stretch of the HAM in the swing phase. In these terms, G represents the gain, l represents the length of the muscle, and l^{tar} represents the constant target length. Both reflex circuits are active only in the swing phase.

Results: Key reflex circuits for energy-efficient walking over wide range velocity, Lines 366-395

The aforementioned results suggest that $G_{HFL}(l_{HFL} - l_{HFL}^{tar})$ and $G_{HAM\_HFL}(l_{HAM} - l_{HAM\_HFL}^{tar})$ could be the parts of the essential reflex circuits on the wide-range velocity walking with maintaining energy efficiency. Therefore, we proceeded to investigate how modulating these reflex circuits affects the energy efficiency of the gait. We measured the change in $\int$ CoT values when the optimized functions of the control parameters associated with $G_{HFL}(l_{HFL} - l_{HFL}^{tar})$ (reflex circuit 1) and $G_{HAM\_HFL}(l_{HAM} - l_{HAM\_HFL}^{tar})$ (reflex circuit 2) were replaced with those derived with $A = 10^6$ in the generated gait with $A = 1$ (a baseline), similar to Fig 11, and the result is shown in Fig 14. The cyan and olive bar represents the relative $\int$ CoT value when we replaced only the optimized functions associated with reflex circuits 1 and 2, respectively. The pink bar represents the relative $\int$ CoT value when we replaced the optimized function of both reflex circuits. The results indicated that the modulation of only these two reflex circuits resulted in a comparable or even more substantial reduction in $\int$ CoT value compared to the modulation of all 56 parameters for the case of $A = 10^6$ shown in the purple bar. This finding strongly suggests that the two length-feedback reflex circuits, $G_{HFL}(l_{HFL} - l_{HFL}^{tar})$ and $G_{HAM\_HFL}(l_{HAM} - l_{HAM\_HFL}^{tar})$, are the key reflex circuits of the energy efficiency in the reflex-based bipedal walking control.

To elucidate the mechanism behind the improved energy efficiency, we examined the change in energy consumption by individual muscles, muscle activation patterns, and stimulation signal for the corresponding muscle when we modulated the two identified reflex circuits. Fig 15 shows the energy consumed by individual muscles during a 30 m walk at $v_x^{tar} = 1.0$. We found that energy consumption of the HFL, GLU, and HAM muscles was significantly reduced after reflex circuits 1 and 2 were modulated. Fig 16 shows the muscle activations of the bipedal model for $v_x^{tar} = 1.0$ m/s. HFL muscle was mainly activated in the swing phase while GLU and HAM muscles were activated in the stance phase. Fig 17 illustrates the stimulation signal applied to HFL muscle during the swing phase before (blue line) and after the modulation (pink line) of reflex circuits 1 and 2: the stimulation signal applied to HFL muscle decreased after the modulation of these identified reflex circuits.

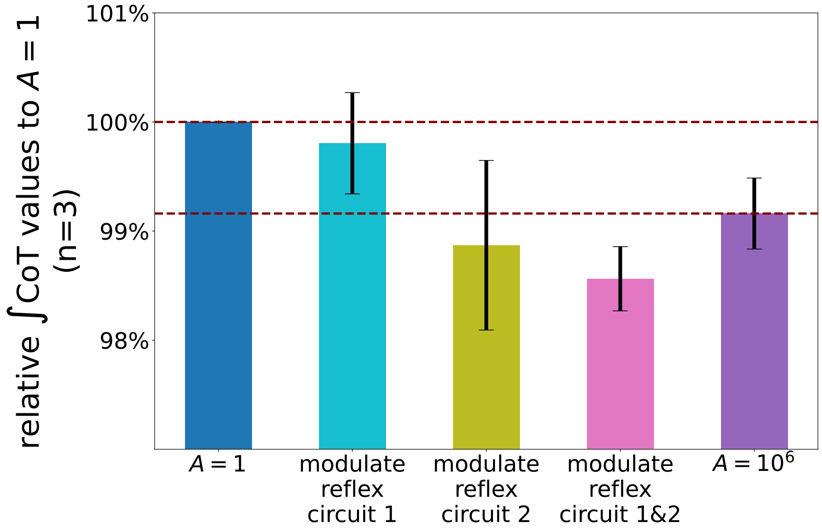

Fig 14. Relative $\int$CoT values to the generated walking with $A = 1$ when modulating identified reflex circuits (n=3). The cyan and olive bar represents the relative $\int$CoT value when only the optimized function of control parameters associated with reflex circuit 1 (i.e. G_{HFL} and l^{tar}_{HFL}) and reflex circuit 2 (i.e. G_{HAM_HFL} and $l^{tar}_{HAM\_HFL}$) were replaced with those derived with $A = 10^6$ in the generated gait with $A = 1$, respectively. The pink bar represents when the optimized function of the control parameters associated with both reflex circuits (i.e., G_{HFL}, l^{tar}_{HFL}, G_{HAM_HFL}, $l^{tar}_{HAM\_HFL}$) were replaced. The purple bar indicates the value in the generated gait using the optimized functions derived with $A = 10^6$. When circuit 2 was modulated, some generated gaits did not follow the specific input v^{tar}_{vel}, with their velocities being changed by more than 0.05 m/s. Data acquired from the target velocity that resulted in these outliers were excluded from the calculation of estimated CoT curves.

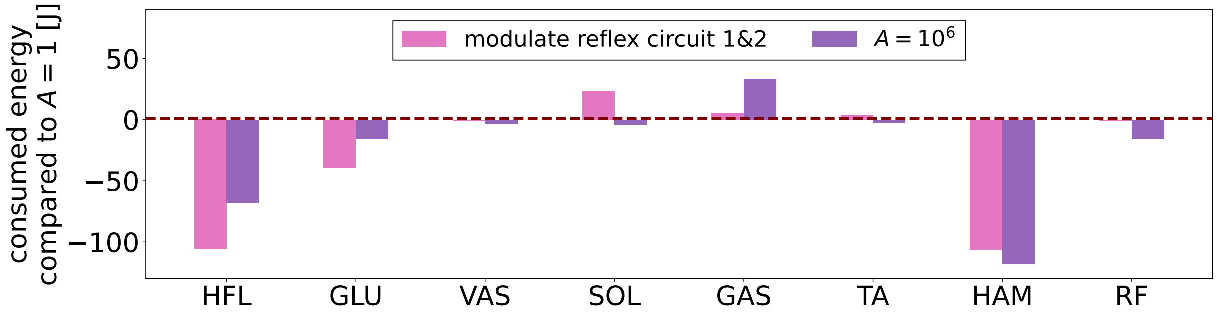

Fig 15. Energy consumed by individual muscles compared to the generated gait with $A = 1$ during a 30 m walk. v^{tar}_{x} was set to 1.0 m/s. The pink bars represent the values when the optimized functions of the control parameters associated with reflex circuits 1 and 2 are replaced with those derived with $A = 10^6$ in the gait generated with $A = 1$, and the purple bars represent the values in the gait with $A = 10^6$. The negative value indicates that the energy consumption at the muscle was reduced compared to the generated gait with $A = 1$, while the positive value indicates increased energy consumption.

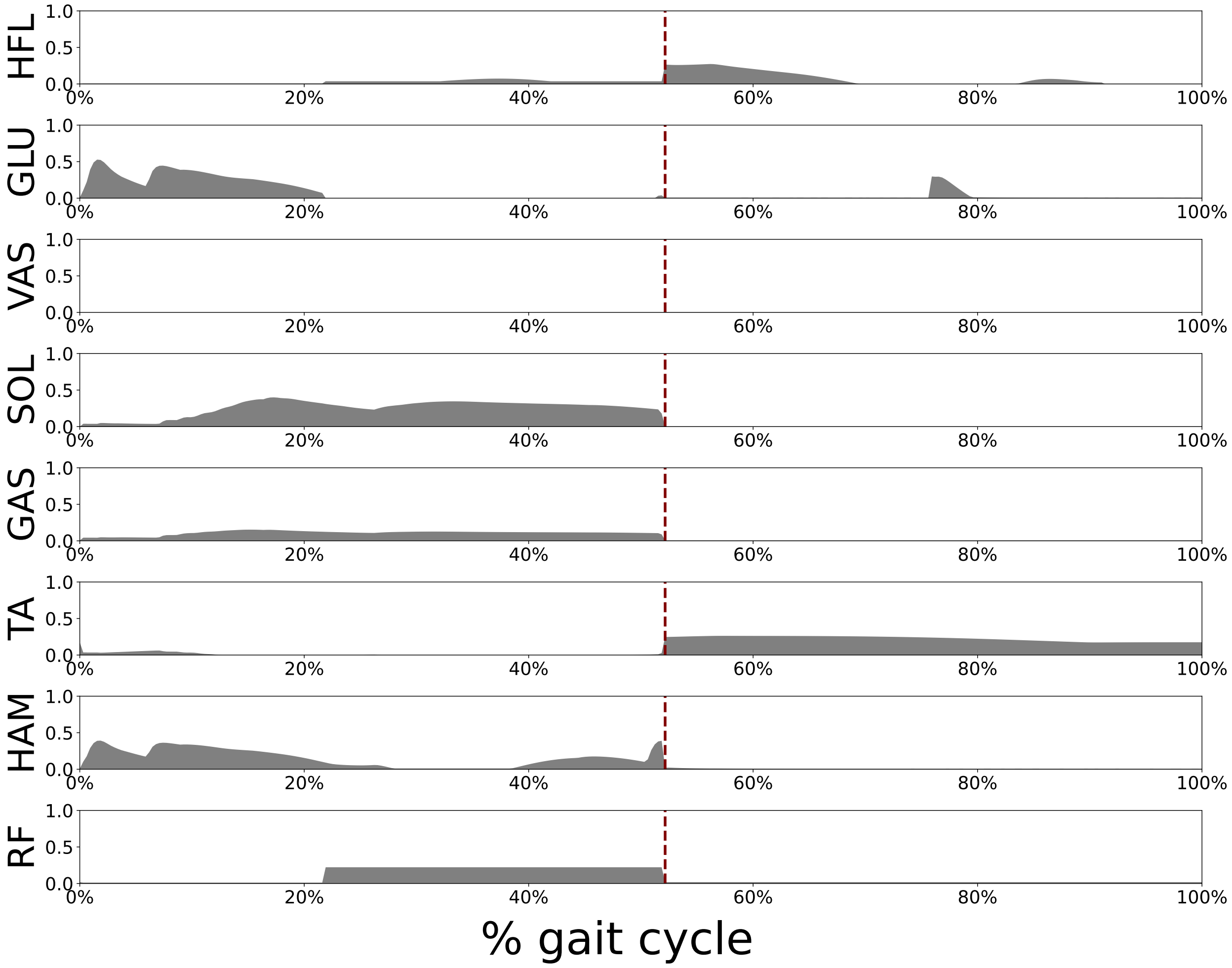

Fig 16. Individual muscle activations. The gait was generated using the optimized functions derived with $A = 10^6$ and v_x^{tar} was set to 1.0 m/s. The red bar indicates the timing of the transition from stance to swing.

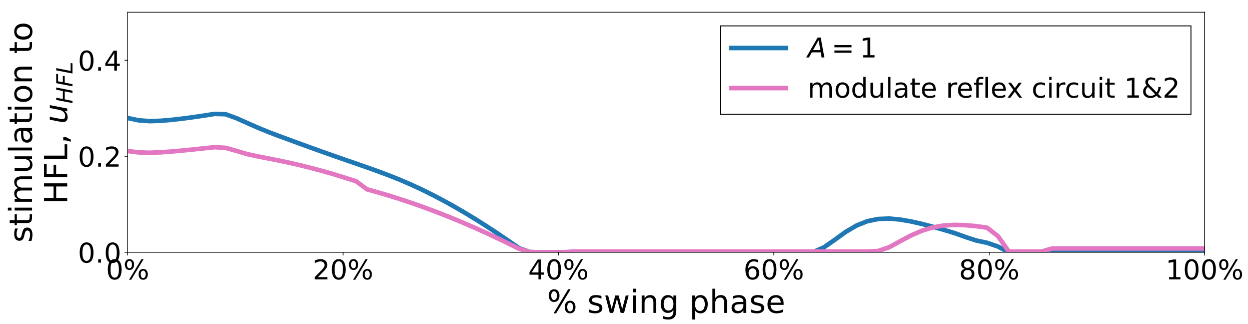

Stimulation to the HFL in the swing phase before and after modulating reflex circuit 1&2. v_x^{tar} was set to 1.0 m/s. The blue and pink lines are the stimulation to the HFL, which does not drop below 0, at generated gait with $A = 1$ and after the optimized function of the control parameters associated with reflex circuit 1 and 2 are replaced with those derived with $A = 10^6$, respectively.

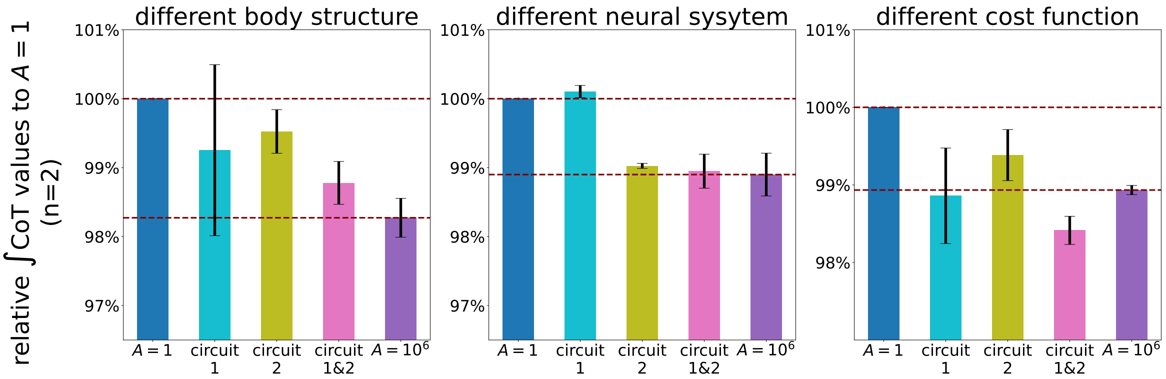

Fig 18. Relative $\int$ CoT values to the generated walking with $A = 1$ under different setting parameters (n=2). In a different body structure setting, the segment lengths of the model were shortened by 20 %. In a different neural system setting, the time delay of the sensory information to the controller was doubled. In this setting, stable gaits were not generated for $A > 100$. In a different cost function setting, the weight coefficients in the objective function (Eq.(31)) were changed to $\alpha_E = 2500$, $\alpha_v = 5$, and $\alpha_t = 0$.

Results, Robustness to parameter changes, Lines 397-409

Under various parameter conditions, leg length, sensory time delay, and weight coefficients for the cost function, we have validated the reliability and robustness of the identified two reflex circuits concerning their impact on energy-efficient walking over a wide range of velocities:

- shorten segment length by 20% (different body structure)
- double the time delay of sensory information transmission to the controller (different neural system)
- change the weight coefficients in the objective cost function (Eq.(31)), $\alpha_E = 2500$, $\alpha_v = 5$, and $\alpha_t = 0$ (different cost function)

Fig 18 illustrates the relative $\int$ CoT values to the baseline ($A = 1$) under various conditions. We found that modulating reflex circuits 1 and 2 resulted in decreased $\int$ CoT values, comparable to the result for all 56 parameter modulation (generated gaits can be seen in S1 Video).

Discussion, Lines 429-445

As shown in Fig 14, we identified that $G_{HFL}(l_{HFL} - l^{tar}_{HFL})$ and $G_{HAM\_HFL}(l_{HAM} - l^{tar}_{HAM\_HFL})$ significantly contributed to improve the energy efficiency of the gait generated through reflex-based control. Furthermore, this conclusion has been robustly validated across various settings, including a different body structure, different neural systems, and different cost functions, as shown in Fig 18. Our findings elucidated that the modulation of these reflex circuits resulted in a reduction of stimulation to the HFL during the swing phase, subsequently leading to reduced energy consumption by the HFL, GLU, and HAM (Fig 15). HFL expended less energy due to reduced stimulation. GLU and HAM were mainly activated during the stance phase (Fig 16), and these two muscles are used to maintain the torso balance in the stance phase [27]. Reduced HFL activity during the swing phase leads to less energy consumption at the contralateral GLU and HAM because the work to compensate for torso acceleration caused by HFL is decreased. Therefore, it can be concluded that minimizing the stimulation to the HFL during the swing phase while ensuring sufficient hip swing to prevent falls is essential to improve energy efficiency in reflex-based walking control. This not only reduces HFL activity but also the effort of the GLU and HAM, which compensates for torso acceleration caused by HFL.

Generating walking through reflex-based control: Reflex-based control, Lines 123-125, 130-132, and 135-139

- **Force feedback:**
  The force feedback law returns the stimulation signal, u^F_i, in response to the actuator force, F_i. In humans, the signals of the F_i arise from Golgi tendon organs and are carried by type Ib afferents to the spinal cord. ...
- **Length feedback**
  From the length feedback, stimulation u^l_i is calculated for the length of the muscle actuator, l_i. This function models the stretch reflex of the muscle spindle. ...
- **Muscle-driven PD control**
  The muscle-driven PD control generates the stimulation necessary to move joint θ_j (Fig 1B, in the main text) to the target angle, θ^{tar}_j. This function can be interpreted as a polysynaptic reflex that is mediated by the joint position afferent input from group III fibers and descending signal of the target joint angles from the supraspinal. ...

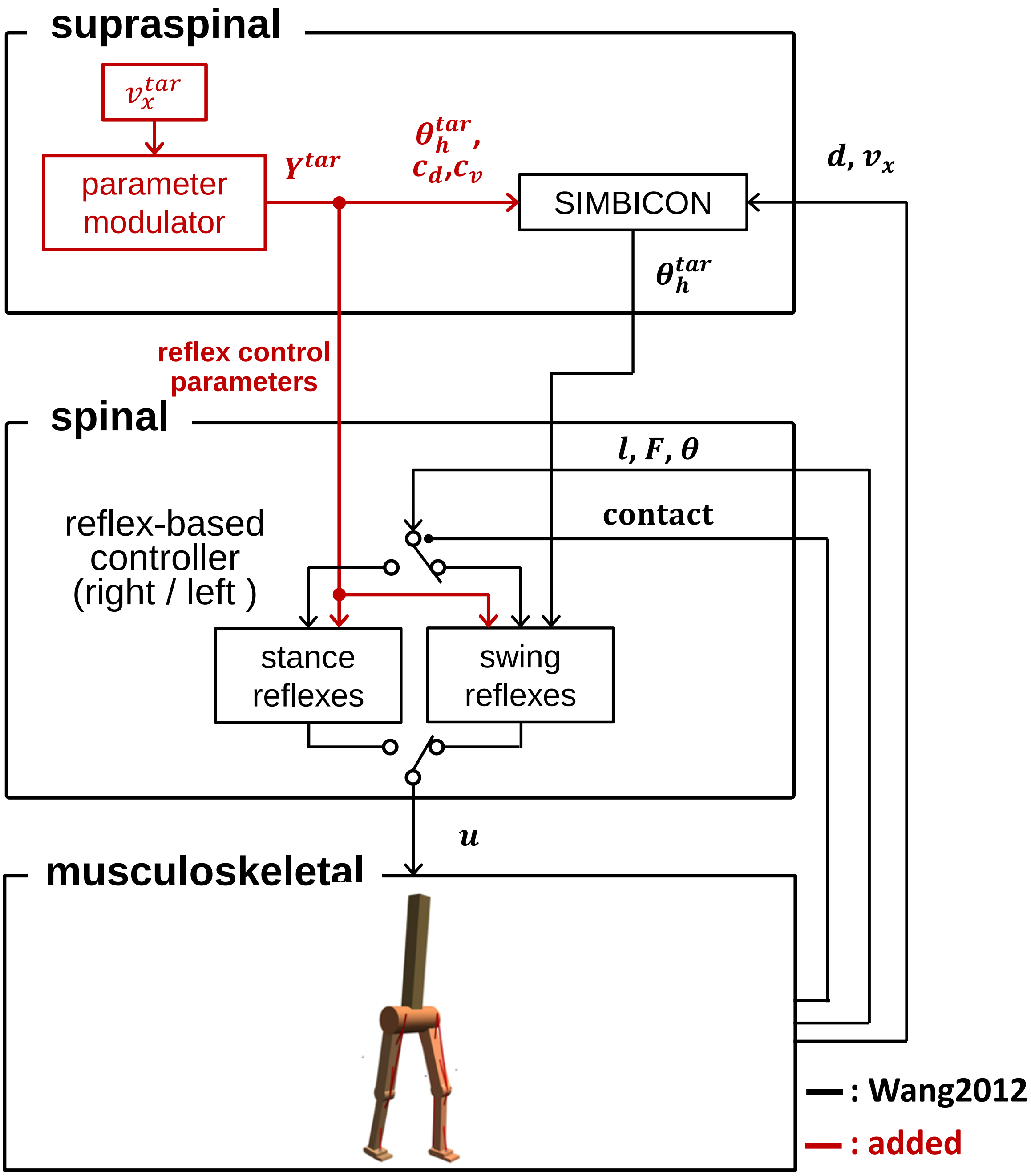

Fig 3. Control diagram for the musculoskeletal model.
The musculoskeletal model is driven by activated muscles receiving stimulation signals, u, from the reflex-based controller in the spinal layer. Each leg (right and left) is controlled by separate stance and swing reflexes. The control law switches depending on whether the leg is in contact with the ground. Sensory information from the model is fed back to the controller for motion generation. The supraspinal layer controls the walking velocity and adjusts the desired foot placements. The parameter modulator coordinates the control parameter set $\boldsymbol{Y}^{tar}$ to achieve input target velocity, v_{vel}^{tar}. SIMBICON adjusts the target hip angle, θ_h^{tar}, in the stance preparation phase using the sensory information (d and v_x, see S1 Appendix.

Comment 5

In general, the paper's organisation makes it hard for the reader to follow the authors reasoning and clearly determine what has been taken from previous work and what is the authors contribution. Some contradictions, e.g. the supposedly very small values for p and q which at times can still reach magnitudes of 0.1 and more and are not compared to the other terms of the controllers, make the comprehension even harder.

Response to Comment 5:

We appreciate the valuable comment. The novel contribution of this study is extending the reflex-based control system to include speed control and identifying key reflex circuits that contribute to energy-efficient walking across wide-range velocities. We have revised Fig 2 to clearly illustrate the aspects adopted from previous work and our contribution, where we have added a parameter modulator that allows a wide range of walking while maintaining the energy efficiency similar to that of the previous model.

We express our gratitude to the reviewer for the comment about p and q value ranges in the reflex-based controller. We have mentioned in lines 129-130 of the previous manuscript that some p and q values were set to enable large values; however, the range of control variable settings varied from previous studies. This might have confused the reviewers. Consequently, we have re-conducted the simulation using the revised model described in the introduction of this response letter.

We hope that the revised manuscript has sufficiently improved over the previous version.

Comments from Reviewer #2

Red font: Reviewer's comments
Black font: Authors' comments
Blue font: Revised or newly added sentences in the revised paper

Major Comment 1

(1) The investigation remains largely exploratory and lacks any clear technical objective to be validated or a scientific question to be answered. The study exploits large (i.e., high-dimensional) solution space with rather arbitrary parameter choices to produce 2D walking kinematics.

(2)-1 In contrary to what the authors identify as a problem (L1-2; L33-36), they still employ a reflex-based controller that involves " vast number of control parameters " (54 in total) and quite a substantial computational overhead (L236-242; L242-243), but add another layer of optimization, namely the performance-weighted least squares (PWLS) parameter modulation method to optimize these control parameters as a function of target velocity while minimizing the CoT.

(2)-2 In doing so, many parameters are chosen based on " trial and error (L230) " or " empirical selection (L246-247) "; some even discussed as main findings (L252-252; L309-319) while many reported without any justification or rationale (L236-242).

(2)-3 Such unprincipled, unsystematic exploration led to only illustrative presentation of the search/optimization results (L235-254; L263-280; Fig 4-7), without any criteria (or expectation) by which the contribution of each step or choice can be assessed.

(3) On a related note, many questionable aspects of the results are left with no interpretation or discussion including the outlier in the solution space (Fig 4), under-/overshoot or errors in terms of tracking desired velocity (Fig 6), and seemingly subtle to no influence of the key parameter (the strength bias parameter A; Fig 7, especially on the final optimized polynomial functions for each control parameter Fig 7C).

(4)Lastly, but not the least - in fact most importantly - when identifying the essential factors for improving energy efficiency, which was presented as one of the main goals for this work (in the Title, Abstract, Summary, Introduction, Discussion, and Conclusion), the authors simply when about replacing the result in optimized parameter modulator from one condition to another (A=1, A=800) and evaluated its impact on CoT, which only resulted in 3% changes in few control parameters (or 5% at most, in one case). I am completely not convinced whether this is the most proper (systematic and rigorous) way to test and demonstrate the key point of the entire work!

Response to Major Comment 1-(1):

We express our sincere gratitude to the reviewer for the helpful and valuable comments and suggestions. The primary scientific question to be answered in this study is "Identifying essential factors for energy-efficient walking control across a wide range of velocities in reflex-based musculoskeletal systems". To achieve this objective, we used the proposed PLWS to identify the reflex circuits that primarily impact energy-efficient walking (**Identifying factors essential for improving energy efficiency**, **Key reflex circuits for energy-efficient walking over wide range velocity**, and **Robustness to parameter changes**) by using the evaluation index of energy efficiency across the wide velocity range (**Performance evaluation of PWLS**). Consequently, we found the fact that the key two reflex circuits have a significant impact on energy-improving efficiency (Fig 13 in the revised manuscript).

Results: Performance evaluation of PWLS, Lines 333-347

In this section, we evaluate the optimization performance of the proposed polynomial regression (PWLS) method. PWLS calculates regression curves by weighting higher-performing (i.e., energy-efficient) data points, in which A determines the strength of the bias toward them. Fig 10 left shows the estimated CoT curves for the generated walking with different A values (A = 1, 10, 10^2, 10^4, and 10^6) in Eq. (41). To quantify the performance of the PWLS, we defined the integrated CoT

value over the corresponding velocity range, $\int \mathrm{CoT}$ as follows:

$$\int \mathrm{CoT} = \int_{v_{x\,min}}^{v_{x\,max}} \mathrm{CoT}\, dv_x, \tag{32}$$

where $v_{x\,min}$ and $v_{x\,max}$ represent the lower and upper limits of the velocity that the model can walk. For example, when the model can walk from $v_{x\,min} = 0.7\,\mathrm{m/s}$ to $v_{x\,max} = 1.6\,\mathrm{m/s}$ and the estimated CoT curve is represented $\mathrm{CoT} = 0.6v_x^2 - 1.2v_x - 0.1$, $\int \mathrm{CoT}$ is calculated as follows:

$$\int \mathrm{CoT} = \int_{v_{x\,min}=0.7\,\mathrm{m/s}}^{v_{x\,max}=1.6\,\mathrm{m/s}} (0.6v_x^2 - 1.2v_x - 0.1)\, dv_x \simeq 0.556. \tag{33}$$

Fig 10 right presents relative $\int \mathrm{CoT}$ value of the generated gaits with different A values. We found that lower $\int \mathrm{CoT}$ for larger A: more energy-efficient walking was generated with larger A. However, excessive A resulted in increased instability: we could not find steady walking at specific v_x^{tar} values for $A > 10^8$.

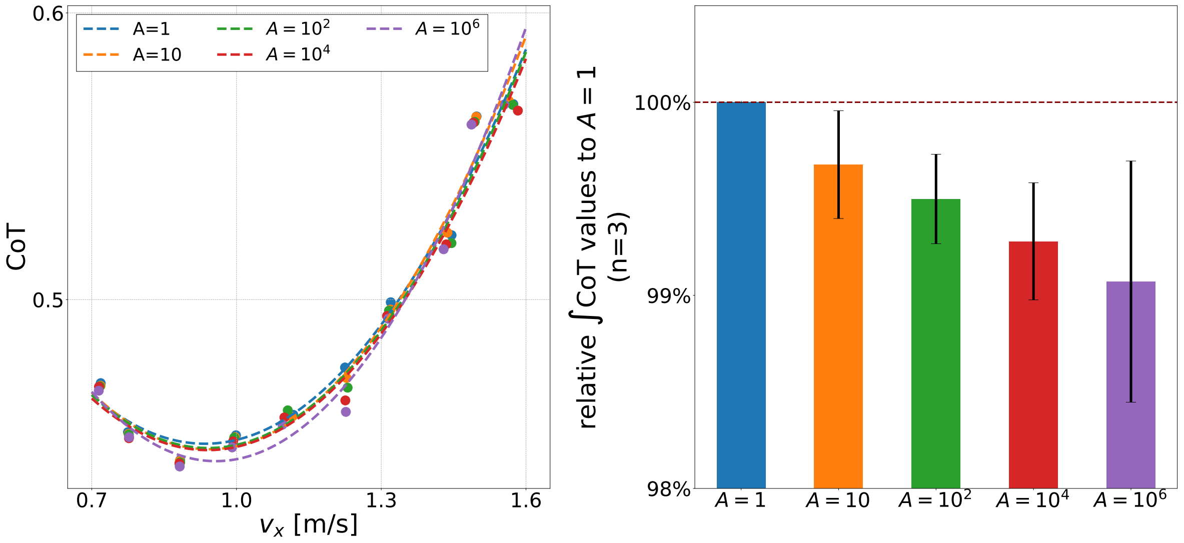

Fig 10. Contribution of the PWLS to gait generation. Left: Estimated CoT curves for generated gaits with different A values. Right: Relative $\int \mathrm{CoT}$ value of the generated gaits with different A values (n=3). The generated gait with $A = 1$ is the baseline. $\int \mathrm{CoT}$ is the integral of the estimated CoT curves from the lower limit velocity to the upper limit velocity.

Results: Identifying factors essential for improving energy efficiency, Lines 349-363

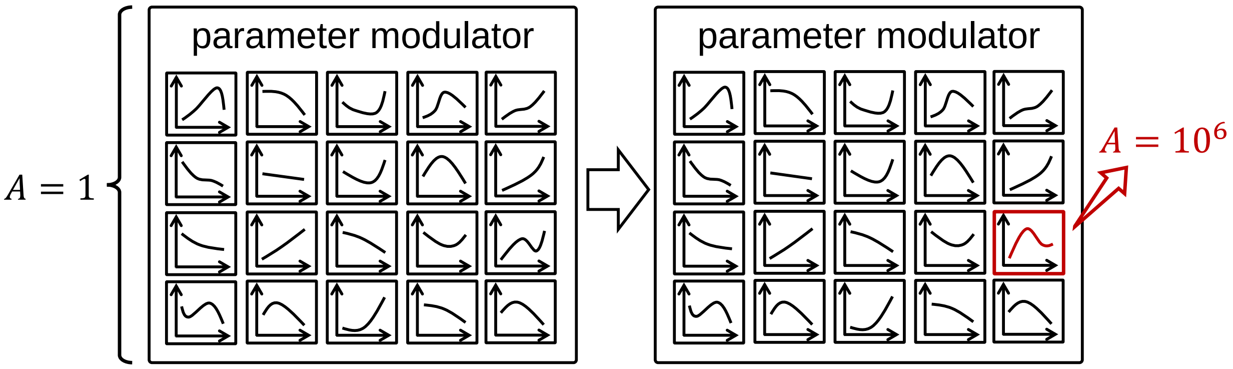

Fig 11. Illustration of the replacement of the optimized function. Left: Before the replacement, all functions were derived with $A = 1$. Right: Only the target control parameter function (red) was replaced with $A = 10^6$. Functions with other parameters were not replaced.

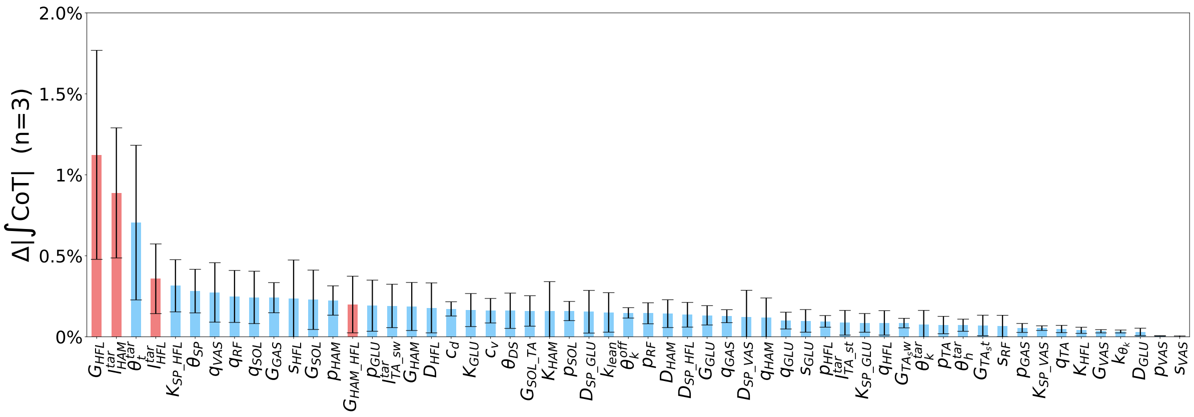

Fig 12. Absolute change in $\int$ CoT values when the optimized function of each parameter was substituted to that derived with $A = 10^6$ in the generated gait with $A = 1$ ($n = 3$). The red bars represent control parameters associated with the reflex circuits under focus.

In this study, we aim to identify the key reflex circuits that influence energy-efficient walking across a wide range of velocities. First, to identify the essential factors for improved energy efficiency, we substituted the optimized function of each control parameter that derived at $A = 10^6$, which achieved more energy-efficient walking over a wide range of velocities, in the gait with $A = 1$ (a baseline). Fig 11 illustrates this approach.

Fig 12 illustrates the absolute change in percentage of the $\int$ CoT values, $\Delta|\int \text{CoT}|$, to the generated gait with $A = 1$. From the results, we found that G_{HFL}, l^{tar}_{HAM} and θ^{tar}_t are the three parameters that changed $\Delta|\int \text{CoT}|$ value by more than 0.5%. This result suggests that reflex circuits that involve these parameters may have a significant impact on energy-efficient walking across a wide range of velocities. The two length-feedback reflex circuits, $G_{HFL}(l_{HFL} - l^{tar}_{HFL})$ and $G_{HAM\_HFL}(l_{HAM} - l^{tar}_{HAM\_HFL})$, is the primary circuits involved in these parameters, G_{HFL} and l^{tar}_{HAM}: both reflex circuits stimulate HFL muscle during the swing phase. θ^{tar}_t, which is the reference angle of the torso, changed $\Delta|\int \text{CoT}|$ value of the third. This parameter was not related to a specific reflex circuit but was shared with several reflex circuits.

Results: Key reflex circuits for energy-efficient walking over wide range velocity, Lines 366-395

The aforementioned results suggest that $G_{HFL}(l_{HFL} - l^{tar}_{HFL})$ and $G_{HAM\_HFL}(l_{HAM} - l^{tar}_{HAM\_HFL})$ could be the parts of the essential reflex circuits on the wide-range velocity walking with maintaining energy efficiency. Therefore, we proceeded to investigate how modulating these reflex circuits

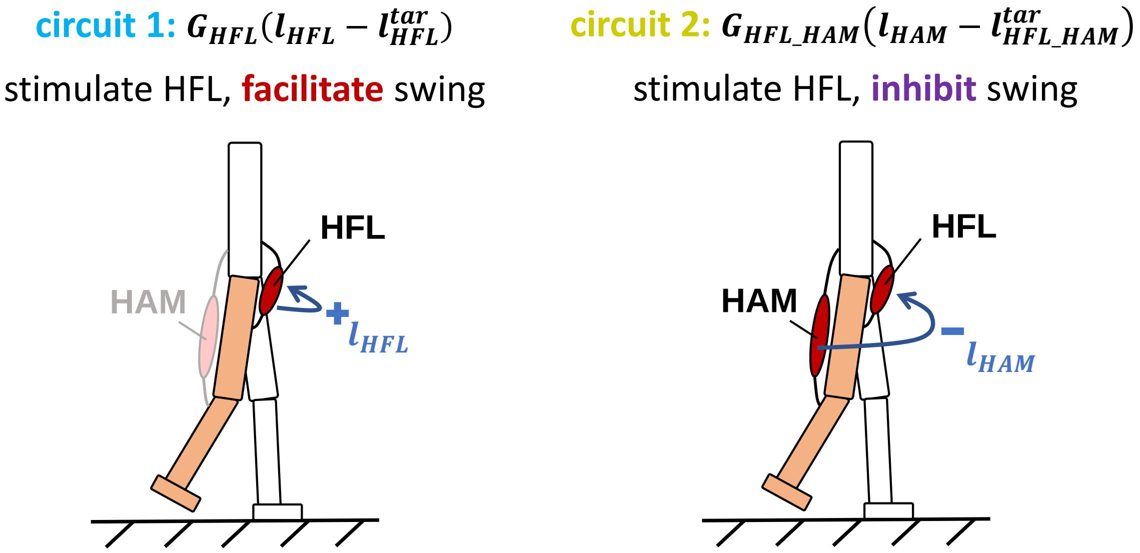

Fig 13. Key reflex circuits for energy-efficient gait. $G_{HFL}(l_{HFL} - l^{tar}_{HFL})$ is the positive length feedback stimulating HFL to swing the leg forward. $G_{HAM\_HFL}(l_{HAM} - l^{tar}_{HAM\_HFL})$ is the negative length feedback inhibiting the HFL proportional to the stretch of the HAM in the swing phase. In these terms, G represents the gain, l represents the length of the muscle, and l^{tar} represents the constant target length. Both reflex circuits are active only in the swing phase.

affects the energy efficiency of the gait. We measured the change in $\int$ CoT values when the optimized functions of the control parameters associated with $G_{HFL}(l_{HFL} - l^{tar}_{HFL})$ (reflex circuit 1) and $G_{HAM\_HFL}(l_{HAM} - l^{tar}_{HAM\_HFL})$ (reflex circuit 2) were replaced with those derived with $A = 10^6$ in the generated gait with $A = 1$ (a baseline), similar to Fig 11, and the result is shown in Fig 14. The cyan and olive bar represents the relative $\int$ CoT value when we replaced only the optimized functions associated with reflex circuits 1 and 2, respectively. The pink bar represents the relative $\int$ CoT value when we replaced the optimized function of both reflex circuits. The results indicated that the modulation of only these two reflex circuits resulted in a comparable or even more substantial reduction in $\int$ CoT value compared to the modulation of all 56 parameters for the case of $A = 10^6$ shown in the purple bar. This finding strongly suggests that the two length-feedback reflex circuits, $G_{HFL}(l_{HFL} - l^{tar}_{HFL})$ and $G_{HAM\_HFL}(l_{HAM} - l^{tar}_{HAM\_HFL})$, are the key reflex circuits of the energy efficiency in the reflex-based bipedal walking control.

To elucidate the mechanism behind the improved energy efficiency, we examined the change in energy consumption by individual muscles, muscle activation patterns, and stimulation signal for the corresponding muscle when we modulated the two identified reflex circuits. Fig 15 shows the energy consumed by individual muscles during a 30 m walk at $v^{tar}_x = 1.0$. We found that energy consumption of the HFL, GLU, and HAM muscles was significantly reduced after reflex circuits 1 and 2 were modulated. Fig 16 shows the muscle activations of the bipedal model for $v^{tar}_x = 1.0$ m/s. HFL muscle was mainly activated in the swing phase while GLU and HAM muscles were activated in the stance phase. Fig 17 illustrates the stimulation signal applied to HFL muscle during the swing phase before (blue line) and after the modulation (pink line) of reflex circuits 1 and 2: the stimulation signal applied to HFL muscle decreased after the modulation of these identified reflex circuits.

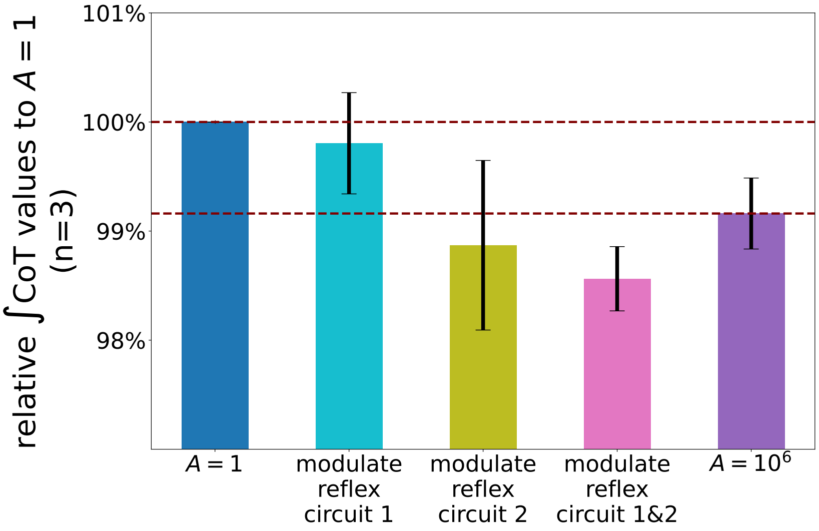

Fig 14. Relative $\int$CoT values to the generated walking with $A = 1$ when modulating identified reflex circuits (n=3). The cyan and olive bar represents the relative $\int$CoT value when only the optimized function of control parameters associated with reflex circuit 1 (i.e. G_{HFL} and l^{tar}_{HFL}) and reflex circuit 2 (i.e. G_{HAM_HFL} and $l^{tar}_{HAM\_HFL}$) were replaced with those derived with $A = 10^6$ in the generated gait with $A = 1$, respectively. The pink bar represents when the optimized function of the control parameters associated with both reflex circuits (i.e., G_{HFL}, l^{tar}_{HFL}, G_{HAM_HFL}, $l^{tar}_{HAM\_HFL}$) were replaced. The purple bar indicates the value in the generated gait using the optimized functions derived with $A = 10^6$. When circuit 2 was modulated, some generated gaits did not follow the specific input v^{tar}_{vel}, with their velocities being changed by more than 0.05 m/s. Data acquired from the target velocity that resulted in these outliers were excluded from the calculation of estimated CoT curves.

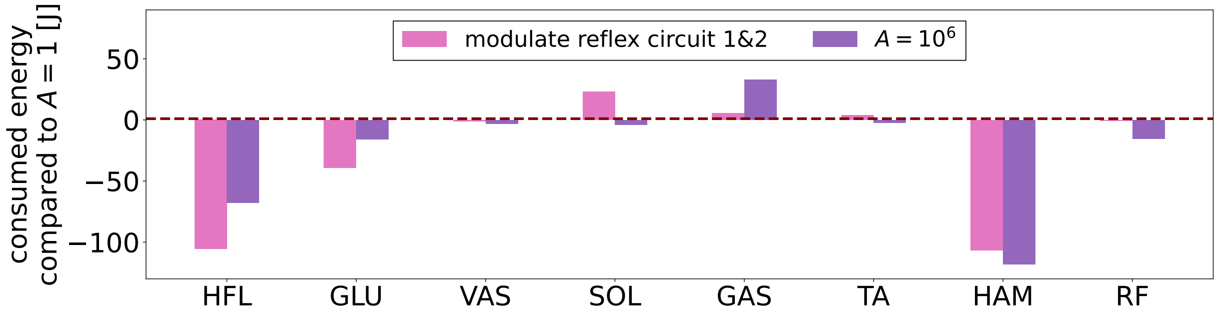

Fig 15. Energy consumed by individual muscles compared to the generated gait with $A = 1$ during a 30 m walk. v^{tar}_x was set to 1.0 m/s. The pink bars represent the values when the optimized functions of the control parameters associated with reflex circuits 1 and 2 are replaced with those derived with $A = 10^6$ in the gait generated with $A = 1$, and the purple bars represent the values in the generated gait with $A = 10^6$. The negative value indicates that the energy consumption at the muscle was reduced compared to the generated gait with $A = 1$, while the positive value indicates increased energy consumption.

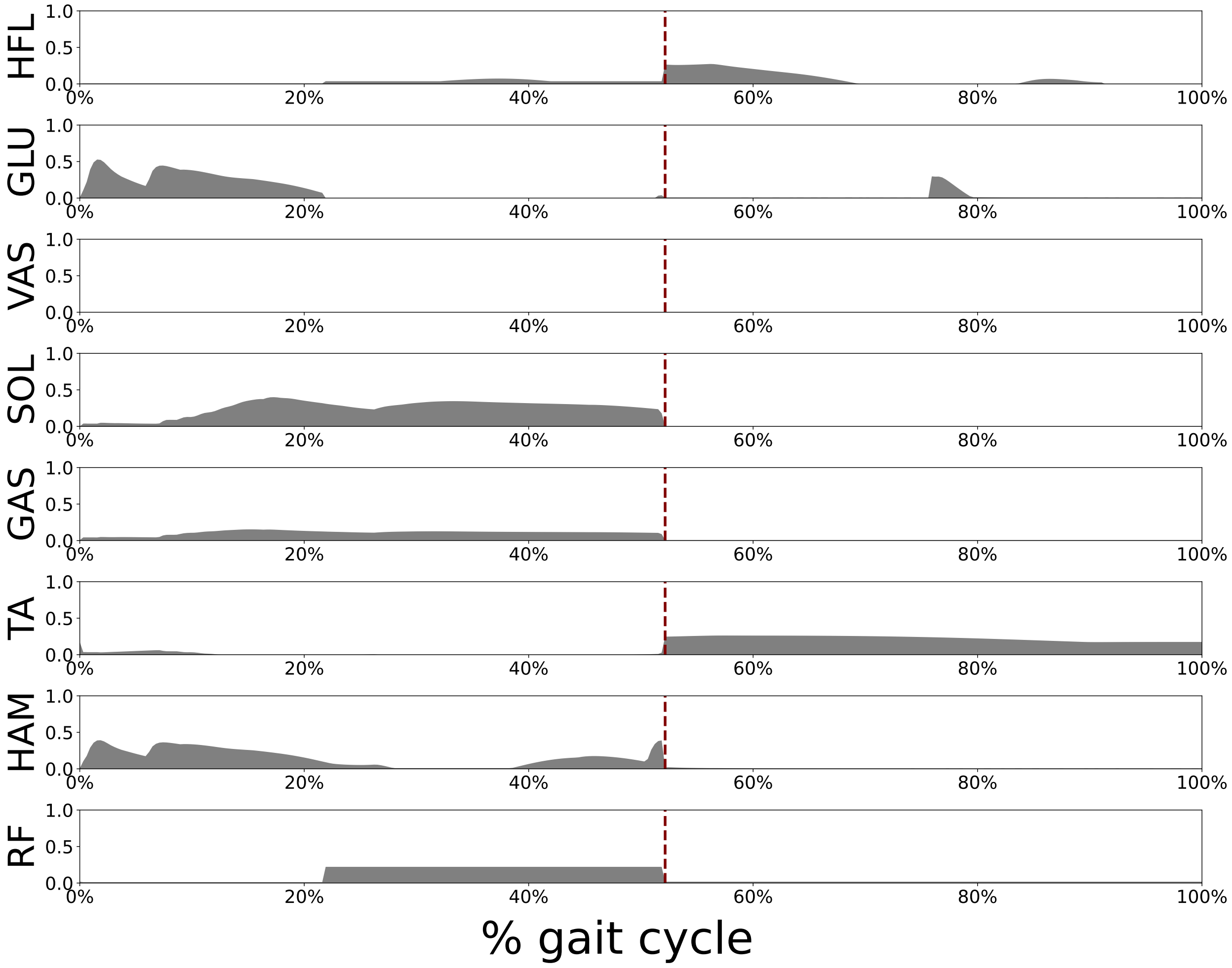

Fig 16. Individual muscle activations. The gait was generated using the optimized functions derived with $A = 10^6$ and v_x^{tar} was set to 1.0 m/s. The red bar indicates the timing of the transition from stance to swing.

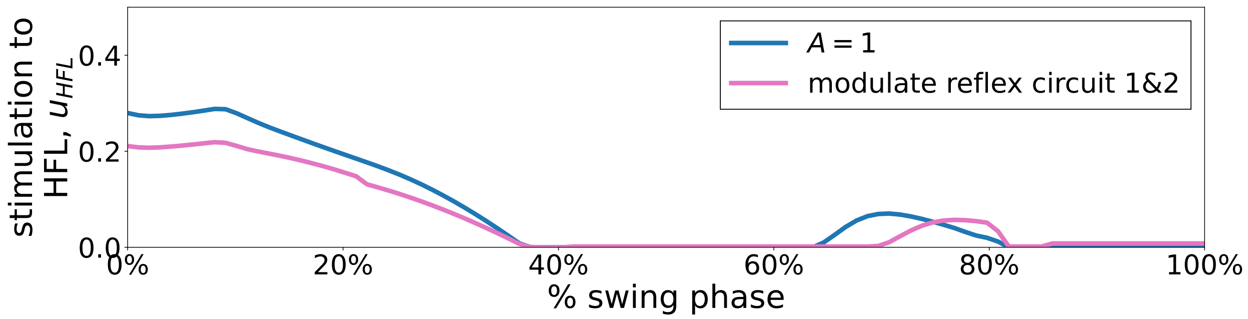

Stimulation to the HFL in the swing phase before and after modulating reflex circuit 1&2. v_x^{tar} was set to 1.0 m/s. The blue and pink lines are the stimulation to the HFL, which does not drop below 0, at generated gait with $A = 1$ and after the optimized function of the control parameters associated with reflex circuit 1 and 2 are replaced with those derived with $A = 10^6$, respectively.

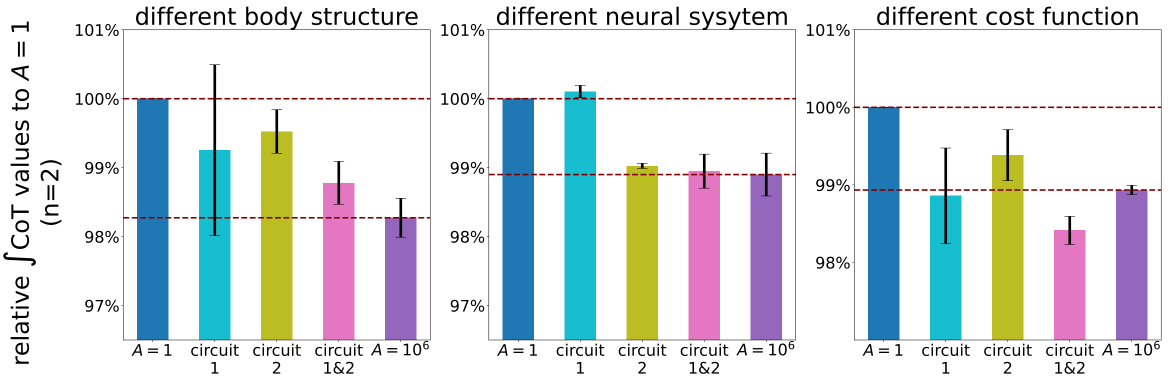

Fig 18. Relative $\int$ CoT values to the generated walking with $A = 1$ under different setting parameters (n=2). In a different body structure setting, the segment lengths of the model were shortened by 20 %. In a different neural system setting, the time delay of the sensory information to the controller was doubled. In this setting, stable gaits were not generated for $A > 100$. In a different cost function setting, the weight coefficients in the objective function (Eq.(31)) were changed to $\alpha_E = 2500$, $\alpha_v = 5$, and $\alpha_t = 0$.

Results, Robustness to parameter changes, Lines 397-409

Under various parameter conditions, leg length, sensory time delay, and weight coefficients for the cost function, we have validated the reliability and robustness of the identified two reflex circuits concerning their impact on energy-efficient walking over a wide range of velocities:

- shorten segment length by 20% (different body structure)
- double the time delay of sensory information transmission to the controller (different neural system)
- change the weight coefficients in the objective cost function (Eq.(31)), $\alpha_E = 2500$, $\alpha_v = 5$, and $\alpha_t = 0$ (different cost function)

Fig 18 illustrates the relative $\int$ CoT values to the baseline ($A = 1$) under various conditions. We found that modulating reflex circuits 1 and 2 resulted in decreased $\int$ CoT values, comparable to the result for all 56 parameter modulation (generated gaits can be seen in S1 Video).

Response to Major Comment 1-(2)-1:

The issue we addressed in the Introduction is not that previous reflex-based controllers have excessive number of control parameters, but that achieving velocity regulation in reflex-based controllers is challenging owing to the requirement for tuning of a large number of control parameters. Therefore, in this study, we developed a reflex-based control framework that enables the regulation of walking velocity across a wide range of speeds. To clarify this, we have revised **Introduction** as follows.

Introduction, Lines 50-56

The purpose of this study is to extend the reflex-based control system to enable controlled walking velocities based on target speeds and gain a better understanding of its modulation and energy-efficient maintenance mechanism across a wide range of walking velocities. To achieve this, we developed a novel performance-weighted least squares (PWLS) method to design a parameter modulator that coordinates a vast number of control parameters for an input target velocity while maintaining energy efficiency.

Response to Major Comment 1-(2)-2:

The hyperparameters for the CMA-ES were set to the recommended values (Hansen, 2015). We described this in the revised manuscript (**Results**). We also added the justifications or rationales of how we choose the values to the empirically chosen parameters (**Design of weight parameters** and **Results**) as follows:

Modulation of control parameters, Design of weight parameters, Lines 274-275

... In this study, M was set to 125, giving $2M + 1 = 250$, which is approximately 0.5% of the control parameter sets collected in the dataset.

Results, Lines 297-302 and 306-309

... For each v_x^{tar}, $\lambda = 20$ points were sampled per generation, with a generation number of $G = 300$, which we confirmed that optimization was converged (See S1 Appendix). This resulted in 102,000 evaluation trials. The other hyperparameters for CMA-ES were set to $\sigma^0 = 0.1$ and $\mu = 7$. λ and μ are recommended value [45]. σ^0 was set to be large, but not out of the solution space in the optimization process. ...

... Using PWLS, we obtained P_i (Fig 4). We found that at least a polynomial degree of $m = 6$ is required to generate gaits. Therefore, to compute polynomials with minimal computational cost and to avoid overfitting, we chose a polynomial degree of $m = 6$.

Response to Major Comment 1-(2)-3:

Because as many as 56 control parameters in the controller exist, many solutions that generate gaits exist, some of which may lead to unnatural and inefficient local minima (Song et al., 2021). To assess the generated gait, we have compared GRFs and kinematics of the generated gaits at small, normal, and fast walking speeds to those of humans. Consequently, we have discussed the calculated cross-correlation values, R, between the generated gaits and humans.

Results: Generated gaits, Lines 318-320

... Fig 8 illustrates the ground reaction forces (GRF) and kinematics of the generated gait using the optimized functions derived with $A = 10^6$ and those of humans at corresponding speeds [57].

Discussion, Lines 453-461

The kinematics of the generated gaits were compared to that of humans as shown in Fig 8. At knee joints, the model straightened the knee earlier in the stance phase compared to humans. This strategy is known to generate more efficient solutions in gait optimization [29]. At ankle joints, the cross-correlation values of ankle dorsiflexion were very low. The previous study also showed

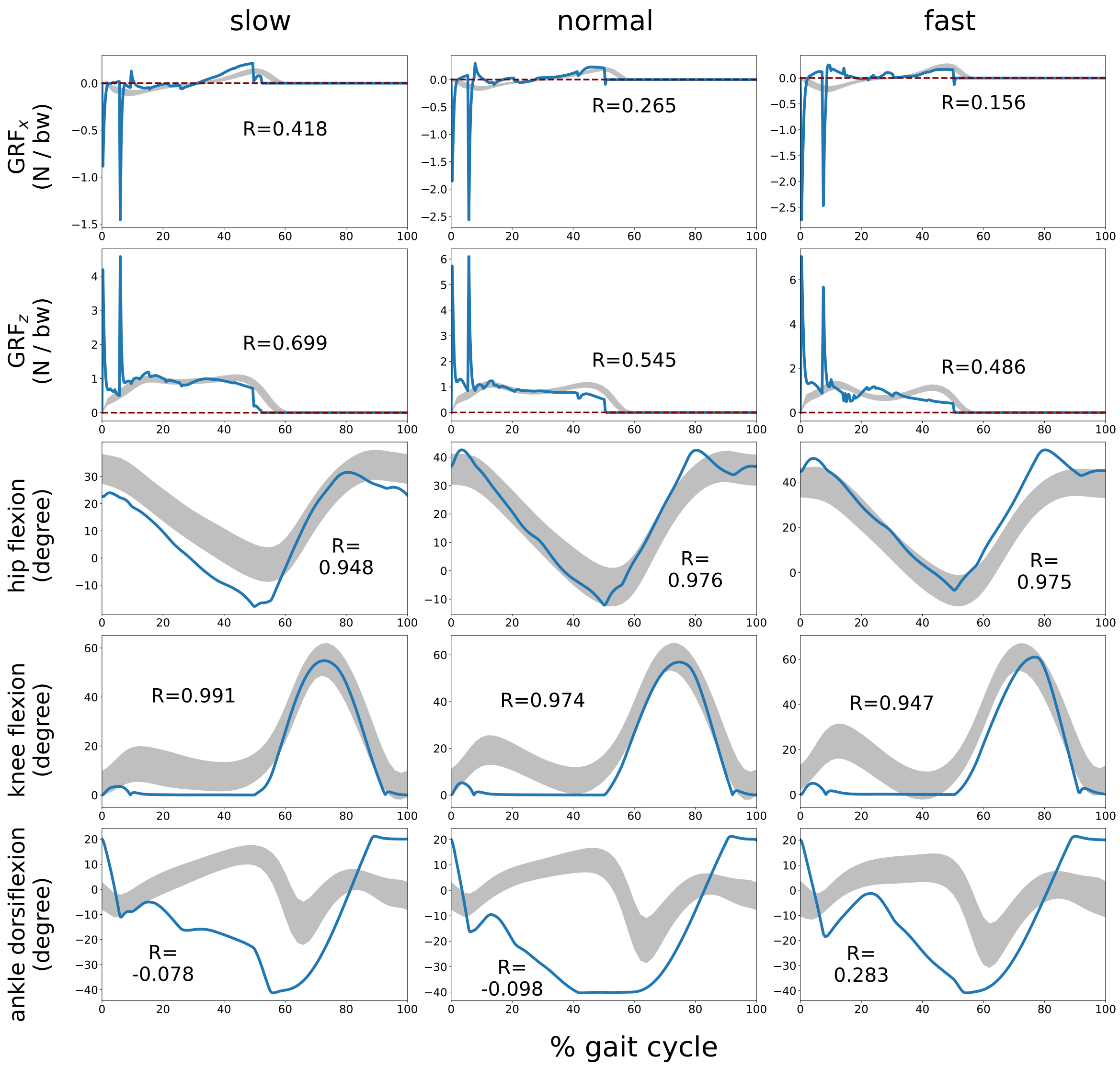

Fig 8.GRFs and kinematics of the generated gait. v_x^{tar} was set to 0.9 (slow), 1.25 (normal), and 1.6 m/s (fast). Blue lines depict the generated gait, while gray lines represent those of humans (mean±1 s.d.) [57]. GRF values are normalized by the body weight. R denotes the cross-correlation value.

low cross-correlation values at ankle joints when optimized to minimize energetic cost [29]. More complex cost function [28] and more human-like body structures [33,34] may be needed to generate human-like ankle joint kinematics.

Response to Major Comment 1-(3):

Fig 5 in the revised manuscript did not show the outlier CoT in the first submission with an unnatural gait because of the simulation re-conducted with the revised model described in the introduction of this response letter. Regarding " under-/overshoot in terms of tracking desired velocity (Fig 6) ", it was the effect of heel strike and toe-off during steady walking, and we have incorporated this point to the **Discussion** as follows:

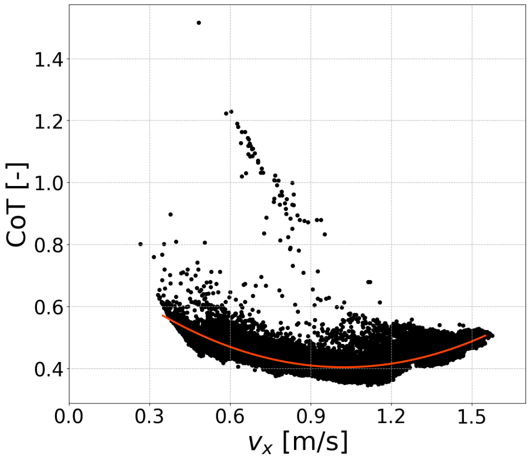

Fig 4 (previous manuscript). Generated walking speeds and their CoTs in the dataset. The CoT velocity value can be approximated by quadratic curves (orange lines) of CoT= $0.367v_x^2 - 0.752v_x + 0.788$.

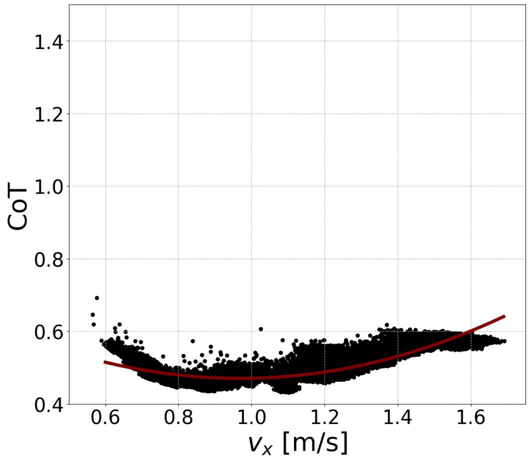

Fig 5. Generated walking speeds and their CoTs in the dataset. The CoT velocity value can be approximated by quadratic curves (red lines).

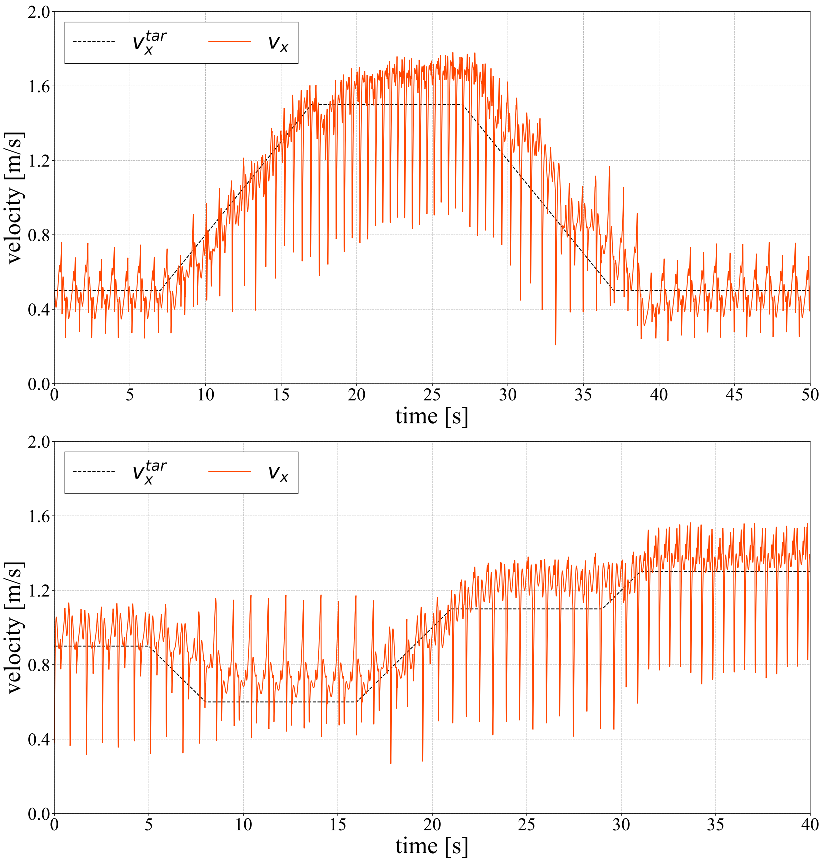

Fig 6 (previous manuscript). Time evolution of the generated walking speed (orange) in response to the target velocity, v_x^{tar} (dotted line). v_x^{tar} was adjusted with a change rate of 0.1 m/s per second. See S1 Video.

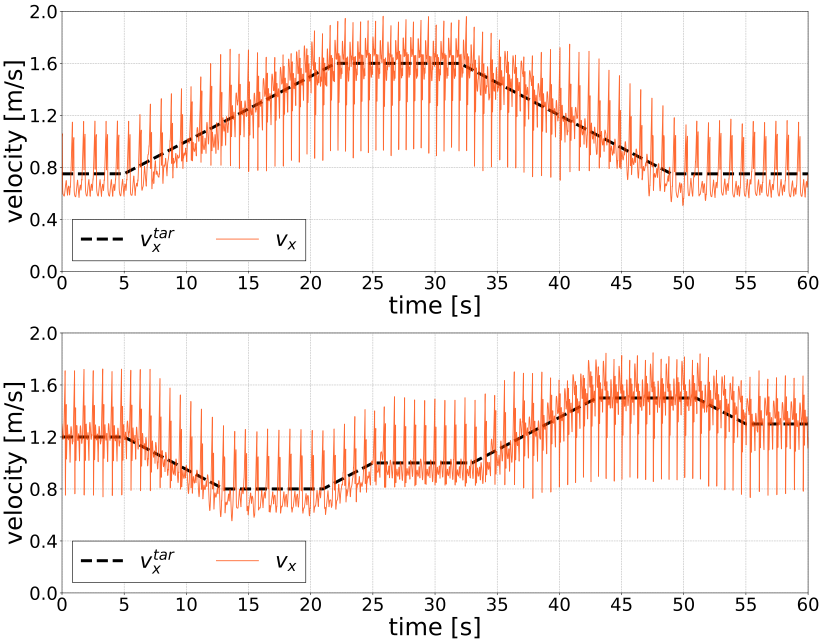

Fig 9. Time evolution of the generated walking speed (orange) in response to the target velocity, v_x^{tar} (dotted line). v_x^{tar} was adjusted with a change rate of 0.05 m/s per second. See S1 Video.

Discussion, Lines 446-453

The generated walking deviates from human walking in some aspects. Overshoots and undershoots were observed in the measured GRFs (Fig 8) and time evolution of the walking velocities (Fig 9). In the GRF profiles, two undershoots exist in GRF_x and two overshoots in exist GRF_z. The first occurred at heel strike and the second occurred at toe-off of the contralateral leg. In the time evolution of the walking velocities, we observed overshoots at toe-off and undershoots at heel strike. These overshoots and undershoots can potentially be mitigated by reducing the simulation timestep [42]. However, employing a smaller timestep leads to increased computational cost.

Response to Major Comment 1-(4):

We are grateful for the valuable comment. Through the additional experiments (**Results**: **Identifying factors essential for improving energy efficiency**, **Key reflex circuits for energy-efficient walking over wide range velocity**, **Robustness to parameter changes**), we have specified our conclusion and contribution to the field related to human biomechanics, "minimizing stimulation to the hip flexor (HFL) during the swing phase, while ensuring sufficient hip swing to prevent falls. This is achieved through the coordinated activity between the HFL and the hamstrings (HAM) during the swing phase, which is a crucial aspect for maintaining energy efficiency in reflex-based walking control across a wide range of velocities." We have also confirmed that these facts are reliable and robust regardless of the parameters used, i.e., leg length, sensory time delay, and weight coefficients in the objective cost function (**Results: Robustness to parameter changes**).

Major Comment 2

The findings are unlikely to generalize to broader (e.g., more realistic) context and the implications do not shed much light on the biological (neural) control of human locomotion. This work employs a 14-segment, 9-DoF (degrees of freedom) model in the sagittal plane. Furthermore, the control is purely based on spinal reflex circuitry from a set of few of representative muscles. This is not to say that simple models are wrong. Such simplified model has been used to elucidate important principles of human locomotion, as in many papers cited by the authors (e.g., Geyer & Herr 2010, IEEE TNSRE) and more (Dzeladini et al., 2014, Front Human Neurosci; Kuo 2002, Motor Control; Taga et al., 1991, Biol Cybern). However, the scope of this paper, including the justification for methodological choices and discussion of the results and implications, lacks any insights beyond the technical increment at hand, i.e., implementation of velocity regulation with extra layer of optimization. For example, one could ask what the implications are for assuming a control that solely relies on feedback (vs. passive mechanics, active feedforward, or their combination) or a fixed set of parameters tuned as a function of walking velocity while minimizing energetic cost (vs. other functional criteria such as stability or effort). On the other hand, what are the principles derived from the examined model that can still hold true in a more generalized context, such as under gravity, physical interaction with the environment (e.g., ground), 3D or whole-body dynamics, viscoelastic tendons, system dynamics (e.g., stability and robustness), in the presence of noise/variability or external disturbances, or across wider range of locomotive behaviors including healthy and pathological? The control of human locomotion involves complex interactions between descending supraspinal neural commands (e.g., corticospinal, reticulospinal), interconnected spinal circuits and its modulation on top of simple length- and force-dependent reflexes, as well as the musculoskeletal system and the environment (Rossignol et al., Physiol Rev, 2006). None of the above aspects are discussed than being listed as yet some other factors influencing human locomotion, only in the Introduction. Moreover, owing to the exploratory approach critiqued above, and due to the very nature of ' optimal ' solutions (Sternad, 2018; Curr Opin Behav Sci) especially in such a high-dimensional parameter space, the findings even within the scope of this paper is unlikely to be generalizable other test conditions. While I agree that the current work holds potential for engineering applications such as control of robots and prosthetics (L10-12; L16-19; L372-373), I cannot agree that the current findings " shed light on the intricate mechanisms underlying human walking (Abstract) ".

Response to Major Comment 2:

Thank you for the critical and constructive comment. As described in the response to comment 1, through the additional experiments (**Results: Identifying factors essential for improving energy efficiency and Key reflex circuits for energy-efficient walking over wide range velocity**), we have identified two key reflex circuits and drawn our conclusion. We have also confirmed that these facts are reliable and robust regardless of the parameters used, i.e., leg length, sensory time delay, and weight coefficients in the objective cost function (**Results: Robustness to parameter changes**).

Results: Identifying factors essential for improving energy efficiency, Lines 349-363

Please see the response to major comment 1.

Results: Key reflex circuits for energy-efficient walking over wide range velocity, Lines 366-395

Please see the response to major comment 1.

Results: Robustness to parameter changes, Lines 397-409

Please see the response to major comment 1.

Locomotion is generated through the tight interaction of the control of human locomotion involves complex interactions between descending supraspinal commands, interconnected spinal circuits including reflexes and CPGs, and the musculoskeletal system. This study mainly focuses on reflexes in the spinal cord and descending modulation signals. To investigate how other control components affect human locomotion control is one of the important future work. We have described this in the **Discussion**.

Discussion, Lines 462-474

Although this study successfully demonstrated the implementation of velocity control in a reflex-based control system, it took 20 s to transit walking speed by 1.0 m/s without falling. Humans, on the other hand, can adjust their walking speed by 1.0 m/s in less than 2 s [59]. Consequently, our control framework lacks the component to change walking speeds rapidly while ensuring stability.

Extending the proposed framework to achieve velocity control as fast as humans is one of the future works. Human locomotion involves complex interactions between descending supraspinal commands, interconnected spinal circuits including reflexes and CPGs, and the musculoskeletal system. This study primarily focuses on reflexes in the spinal cord. Therefore, to achieve fast walking speed control, we consider including other control components. Moreover, the bipedal model motion was constrained to the sagittal plane. Hence, the extension of the motion into three dimensions is another important future work to apply the controller to engineering applications.

We believe that this robust conclusion and contribution to the field related to human biomechanics can "shed light on the intricate mechanisms underlying human walking".

Minor Comment 1

I cannot agree, or at least cannot understand, the statement " limited computational capacities in neural systems cannot handle the numerous parameter changes needed for each point-to-point velocity transition. (L39-41) ". Please elaborate or clarify.

Response to Minor Comment 1:

We are grateful for the insightful comment. Previous studies have succeeded in transitions between predetermined walking velocities within the reflex-based control frameworks (Song et al., 2012, Song et al., 2015). However, in these studies, transitions between predetermined velocities were achieved by using a control parameter set for the transition in addition to the steady gait. Considering that humans continuously change the walking velocity, precise control of the walking velocity would theoretically require an infinite number of prepared parameter sets for transitions based on the previous approach. From a computational perspective, learning and memorizing an infinite set of parameters is inefficient. We have described this as "limited computational capacities in neural systems cannot handle the numerous parameter changes needed for each point-to-point velocity transition". To clearly explain this point, we have revised the manuscript as follows.

Introduction, Lines 36-46

Previous studies have attempted to regulate velocities within the reflex-based control frameworks [29,31], but their methods were limited to transitions between predetermined velocities and did not provide precise velocity controls. Furthermore, the transitions between these predetermined velocities were accomplished through the utilization of distinct control parameters designated for these transitions. Given that walking speed assumes continuous values, achieving precise control over walking speed through such means would theoretically require infinite parameters for transitions. Because "transition" differs from "control" in terms of its adaptability, it is essential to extend reflex-based systems to affect precise velocity controls while improving energy efficiency to explore reflex control mechanisms in walking. ...

Minor Comment 2

If the model, and especially the reflex-based controller is essentially the same as what's been provided in previous works, I suggest that the authors simply cite those work. At the minimum, some portions can be moved to the Supplementary materials. This way, the novel contribution of this work, which is the additional PWLS method used to implement velocity regulation (or control), can be also highlighted better.

Response to Minor Comment 2:

Thank you for the valuable suggestion. The reflex-based controller employed in this study is identical in principle to that of Wang et al. (Wang et al., 2012). We did not make ad-hoc adjustments to the basic reflex-based control model to ensure optimal simulation performance. In the revised manuscript, because the reflex-based controller is identical to that used in the previous work, we have described the overview of the controller in the main manuscript and explained the detailed equations in the Supplementary material to clarify and highlight the novel contribution of this work. The novel contribution of this work is to extend the

reflex-based control system including speed control and identify key reflex circuits that play a significant role in energy-efficient walking. Therefore, we believe that this movement of sentences can highlight these contributions better.

Generating walking through reflex-based control: Reflex-based control, Lines 113-149

The reflex-based controller employed in this study is identical in principle to that of Wang et al. [28]. We did not make any ad-hoc adjustments to the basic reflex-based control model to ensure optimal simulation performance. The controller computes the muscle stimulation, denoted as u_i, for each muscle, i, by incorporating sensory feedback with a time delay of Δt as input. The control law switches depending on whether the leg is in the stance or swing phase. Moreover, additional stimulation is introduced during the late stance and late swing phases. The control system comprises three primary functions: force feedback, length feedback, and muscle-driven proportional derivative (PD) control.

- Force feedback:
  The force feedback law returns the stimulation signal, u_i^F, in response to the actuator force, F_i. In humans, the signals of the F_i arise from Golgi tendon organs and are carried by type Ib afferents to the spinal cord. u_i^F is calculated as follows:

$$u_i^F = G_i \tilde{F}_i(t - \Delta t_i), \tag{34}$$

$$\tilde{F}_i(t - \Delta t_i) = \frac{F_i(t - \Delta t_i)}{F_i^0}, \tag{35}$$

  where the gain, G_i, is the positive control parameter, and $\tilde{F}_i(t - \Delta t_i)$ is the scaled actuator force, (i.e., $\tilde{F}_i = F_i / F_i^0$), which includes the sensory time delay, Δt_i.

- Length feedback
  Through length feedback, we calculate the stimulation signal, denoted as u_i^l, corresponding to the length of the muscle actuator, l_i. This function models the stretch reflex of the muscle spindle. u_i^l is calculated as follows:

$$u_i^l = \max\{0, G_i(l_i(t - \Delta t_i) - l_i^{tar})\}, \tag{36}$$

  where the target length, l_i^{tar}, and gain, G_i, are positive control parameters.

- Muscle-driven PD control
  The muscle-driven PD control generates the stimulation necessary to move joint θ_j (Fig 1B) to the target angle, θ_j^{tar}. This function can be interpreted as a polysynaptic reflex that is mediated by the joint position afferent input from group III fibers and descending signals of the target joint angles from the supraspinal. The muscle-driven PD control laws are employed to stabilize the generated gait by the hip muscles during the stance phase to balance the torso and during stance preparation to prepare for ground contact (described in S1 Appendix). For muscle actuator, i, which applies torque to joint θ_j in the positive direction, $u_i^{\theta_j}$ is defined as follows:

$$u_i^{\theta_j} = \max\{0, K_i(\theta_j^{tar} - \theta_j(t - \Delta t_i)) - D_i \dot{\theta}_j(t - \Delta t_i)\}. \tag{37}$$

  Conversely, for muscle actuator i, which applies torque in the negative direction, u_i^{θ} is defined as follows:

$$u_i^{\theta} = \max\{0, K_i(\theta_j(t - \Delta t_i) - \theta_j^{tar}) + D_i \dot{\theta}_j(t - \Delta t_i)\}, \tag{38}$$

  where the proportional gain, K_i, and derivative gain, D_i, are positive control parameters.

Fig 2 illustrates the reflexes in the stance phase and swing phase. Please see S1 Appendix or the original paper [28] for more details.

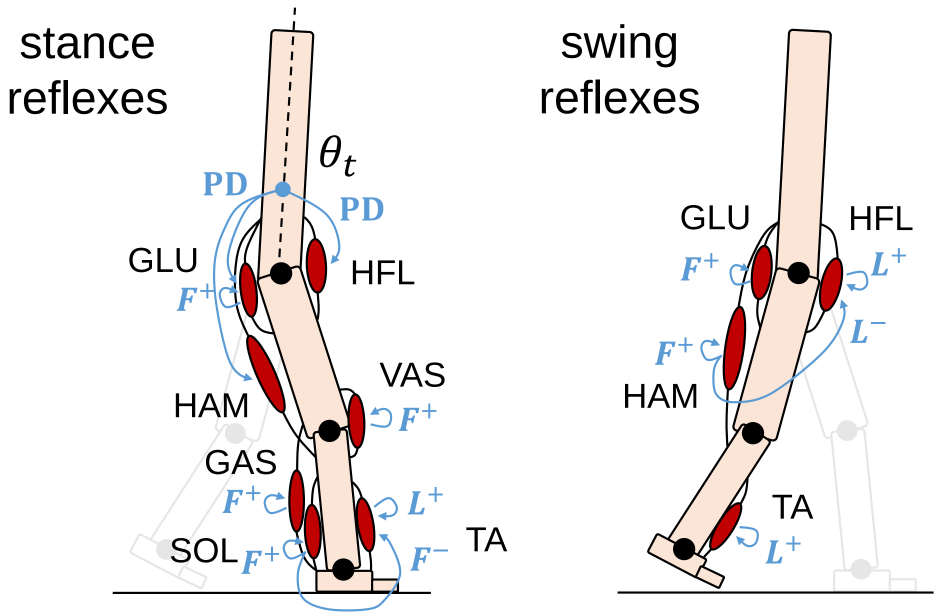

Fig 2. Key reflexes in the stance and swing phase. F represents force feedback, L represents length feedback, and PD represents muscle-driven PD control. $+$ and $-$ denote positive and negative feedback, respectively. In the stance phase, F^+ at GLU, VAS, and SOL generate compliant leg behavior. L^+ at TA prevents overextension of the ankle joint, which is suppressed by the F^- from the SOL. F^+ at GAS contributes to push-off and prevents overextension of the knee joint. Muscle-driven PD controls at HFL, GLU, and HAM balance the torso. In the swing phase, L^+ facilitates leg swing, which is suppressed by L^- from HAM. F^+ at GLU and HAM apply braking force to the swing leg. L^+ at TA raises the toes to create clearance between the feet and the ground.

Supplementary material: Reflex-based control, Lines 8-38

Stance Phase

During the stance phase, the stimulation, u_i, to each muscle actuator is calculated as follows:

$$
\begin{aligned}
u_{SOL} &= p_{SOL} + G_{SOL}\tilde{F}_{SOL}(t - \Delta t_l), && (39)\\
u_{TA} &= p_{TA} + \max\{0, G_{TA\_st}(l_{TA}(t - \Delta t_l) - l^{tar}_{TA\_st})\} - G_{SOL\_TA}\tilde{F}_{SOL}(t - \Delta t_l), && (40)\\
u_{GAS} &= p_{GAS} + G_{GAS}\tilde{F}_{GAS}(t - \Delta t_l), && (41)\\
u_{VAS} &= p_{VAS} + G_{VAS}\tilde{F}_{VAS}(t - \Delta t_m) + f_{VAS}, && (42)\\
f_{VAS} &= \begin{cases} 0 & (\theta_k < \theta^{off}_k) \\ k_{\theta_k}\{\theta_k(t - \Delta t_{VAS}) - \theta^{off}_k\} & (\theta_k \geq \theta^{off}_k) \end{cases}, && (43)\\
u_{HAM} &= p_{HAM} + \max\{0, K_{HAM}(\theta_t(t - \Delta t_s) - \theta^{tar}_t) + D_{HAM}\dot{\theta}_t\}, && (44)\\
u_{RF} &= p_{RF}, && (45)\\
u_{GLU} &= p_{GLU} + \max\{0, K_{GLU}(\theta_t(t - \Delta t_s) - \theta^{tar}_t) + D_{GLU}\dot{\theta}_t\}, && (46)\\
u_{HFL} &= p_{HFL} + \max\{0, K_{HFL}(\theta^{tar}_t - \theta_t(t - \Delta t_s)) + D_{HFL}\dot{\theta}_t\}, && (47)
\end{aligned}
$$

where p_i are positive constant control parameters that are approximate to zero. The time delays, Δt_l, Δt_m, and Δt_s, are set to 20, 10, and 5 ms, respectively. The shorter transmission pathway to the spinal cord results in smaller time delays; therefore, the muscles near the hip joint have minimal time delays. Positive force feedback at GLU, VAS, and SOL contributes to compliant leg behavior. When the gains, denoted as G, are set higher, the stance leg emulates an inverted pendulum model. Conversely, when the gains are set at smaller values, it behaves as a spring-inverted pendulum model [3]. Positive length feedback at TA prevents overextension of the ankle joint. This action is suppressed by negative force feedback from the SOL, which serves to extend the ankle joint during a push-off. Positive force feedback at GAS contributes to push-off and prevents overextension of the knee joint. Muscles around the hip joint, namely HFL, GLU, and HAM, function together to maintain the reference lean angle θ^{tar}_t through muscle-driven PD controls, thus stabilizing the torso. The term, f_{VAS}, prevents knee hyperextensions during the mid-stance phase. Stimulation of the VAS is suppressed when the knee joint angle, θ_k, exceeds the threshold angle, θ^{off}_k. During the swing initiation phase, when the hip extension angle, θ_h, exceeds θ_{SI}, as depicted in Fig S1A, constant stimulation values s_i that are greater than zero are added to the VAS, RF, GLU, and HFL to encourage swing initiation [4].

$$
\begin{aligned}
u_{VAS} &\leftarrow u_{VAS} - s_{VAS}, && (48)\\
u_{RF} &\leftarrow u_{RF} + s_{RF}, && (49)\\
u_{GLU} &\leftarrow u_{GLU} - s_{GLU}, && (50)\\
u_{HFL} &\leftarrow u_{HFL} + s_{HFL}. && (51)
\end{aligned}
$$

Swing Phase

During the swing phase, the stimulation, u_i, to each muscle actuator is calculated as follows:

$$
\begin{aligned}
u_{SOL} &= q_{SOL}, && (52)\\
u_{TA} &= q_{TA} + \max\{0, G_{TA\_sw}(l_{TA}(t - \Delta t_l) - l^{tar}_{TA\_sw})\}, && (53)\\
u_{GAS} &= q_{GAS}, && (54)\\
u_{VAS} &= q_{VAS}, && (55)\\
u_{HAM} &= q_{HAM} + G_{HAM}\tilde{F}_{HAM}(t - \Delta t_s), && (56)\\
u_{RF} &= q_{RF}, && (57)\\
u_{GLU} &= q_{GLU} + G_{GLU}\tilde{F}_{GLU}(t - \Delta t_s), && (58)\\
u_{HFL} &= q_{HFL} + \max\{0, G_{HFL}(l_{HFL}(t - \Delta t_s) - l^{tar}_{HFL})\} \\
&\quad - \max\{0, G_{HAM\_HFL}(l_{HAM}(t - \Delta t_s) - l^{tar}_{HAM})\} + f_{HFL}, && (59)\\
f_{HFL} &= k_{lean}\{\theta^{tar}_t - \theta_t(t - \Delta t_{HFL})\}, && (60)
\end{aligned}
$$

where q_i are positive constant control parameters similar to p_i and also approximate zero. Positive length feedback at HFL facilitates leg swing, which is suppressed by negative length feedback from HAM in the mid-swing phase. The term f_{HFL} stimulates the HFL according to the current torso angle, θ_t, and the torso target angle, θ_t^{tar}. The required protraction speed relies on the torso lean. Positive force feedback at GLU and HAM applies braking force to the swinging leg, transferring some of the angular momentum into leg lowering and retraction. Positive length feedback at TA raises the toes, ensuring sufficient clearance between the feet and the ground. During the stance preparation phase, when the hip flexion angle, θ_h, exceeds θ_{SP}, as depicted in Fig S1A, the swinging leg transitions into stance preparation. Muscle-driven PD controls are integrated into the VAS, GLU, and HFL to produce the desired pose for touchdown in this phase.

$$\begin{aligned} u_{VAS} &\leftarrow u_{VAS} \\ &\quad + \max\{0, K_{SP\_VAS}(\theta_k(t-\Delta t_m) - \theta_k^{tar}) + D_{SP\_HFL}\dot{\theta}_k(t-\Delta t_m)\}, \quad (61) \\ u_{GLU} &\leftarrow u_{GLU} \\ &\quad + \max\{0, K_{SP\_GLU}(\theta_h^{tar} - \theta_h(t-\Delta t_m)) + D_{SP\_GLU}\dot{\theta}_h(t-\Delta t_m)\}, \quad (62) \\ u_{HFL} &\leftarrow u_{HFL} \\ &\quad + \max\{0, K_{SP\_HFL}(\theta_h(t-\Delta t_m) - \theta_h^{tar}) + D_{SP\_HFL}\dot{\theta}_h(t-\Delta t_m)\}. \quad (63) \end{aligned}$$

To reproduce a stable gait, the target hip angle, θ_h^{tar}, is adjusted according to the balance feedback law (SIMBICON) [5]:

$$\theta_h^{tar} = \theta_h^{tar} - c_d d + c_v v_x, \quad (64)$$

where d represents the horizontal distance from the stance ankle to the hip segment, as depicted in Fig S1B. v_x represents the horizontal velocity of the model, and c_d and c_v are positive constant parameters. This balance feedback law can be interpreted as the supraspinal control that uses sensory information from the vestibular organs.

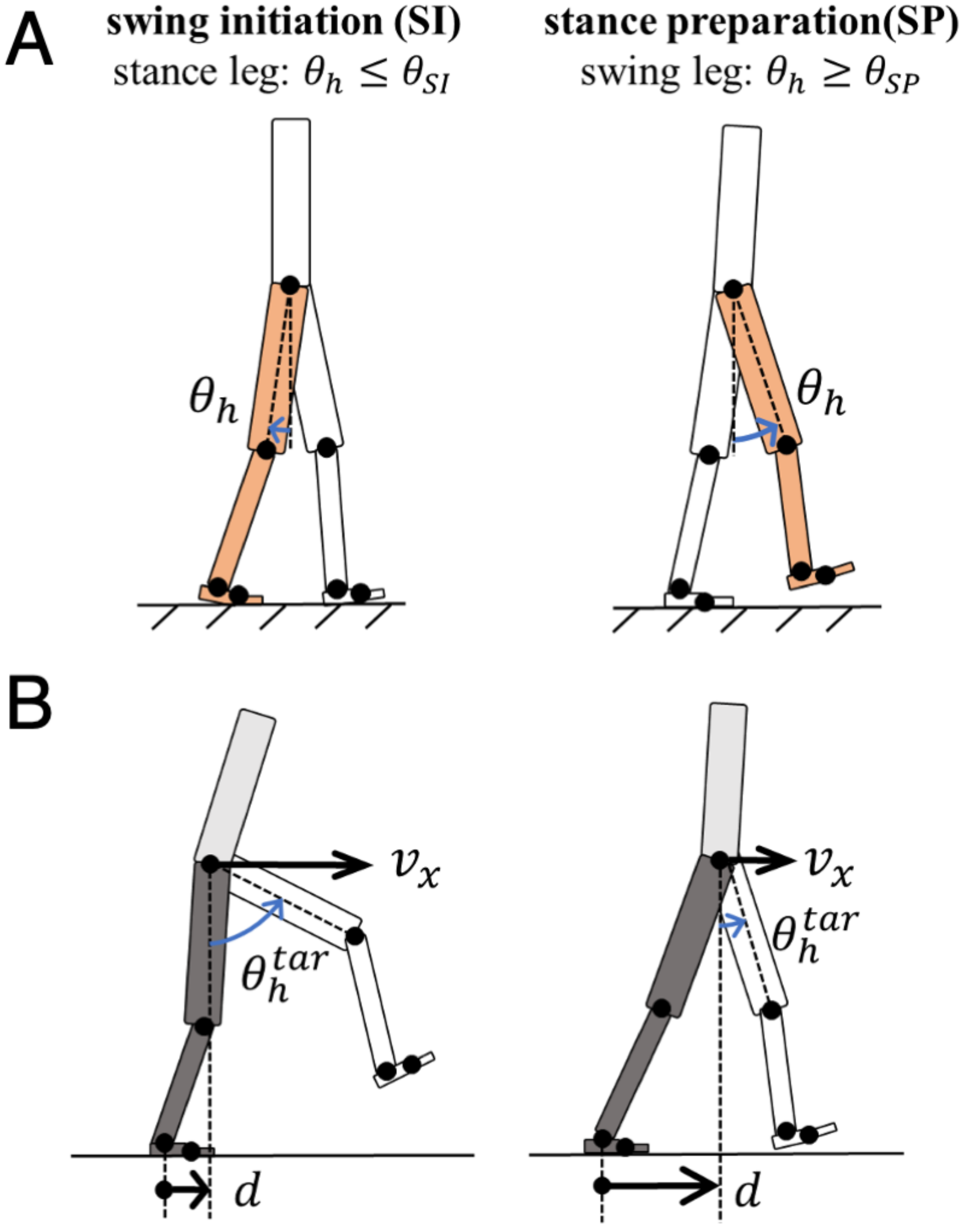

Fig S1: Description of the reflex-based control. A. Transition conditions of swing initiation (SI) and stance preparation (SP) phases. The SI phase commences when the hip extension angle, θ_h, of the stance leg exceeds a predetermined threshold, θ_{SI}. The SP phase commences when the hip flexion angle, θ_h, of the swing leg exceeds the threshold, θ_{SP}. **B. Diagram of the balance feedback law.** The feedback control system dynamically adjusts the target hip angle, θ_h^{tar}, depending on the horizontal distance from the stance ankle to the hip segment, d, and the horizontal model velocity, v_x. For instance, when d is small or v_x is large, θ_h^{tar} is increased, causing the foot to be placed more forward to prevent falling, as depicted on the left. Conversely, when d is large or v_x is small, θ_h^{tar} is decreased.

Minor Comment 3

Given the extensive and detailed methods, I request the authors use more organized/structured section headings for better readability, e.g., numbered rather than just by font sizes.

Response to Minor Comment 3:

Thank you for the valuable suggestion. In the format of PLOS Computational Biology, numbered section heading is not used. In the previous manuscript, the subsection of **Reflex-based control** has a sub-sub section, which may decrease readability. Consequently, we have revised the manuscript as carefully explained in minor comment 2 and excluded sub-subsections. Furthermore, we have divided the subsection of **Identifying factors essential for improving energy efficiency** into three parts: **Identifying factors essential for improving energy efficiency**, **Key reflex circuits for energy-efficient walking over wide range velocity**, and **Robustness to parameter changes,** to clearly show what was done for what purpose and what results were obtained. We believe these revisions have enhanced readability.

Minor Comment 4

Presenting and discussing the results not only in terms of control parameters, costs (in objective function), and outputs, but also in terms of biomechanics (kinematics) and neural control (muscle activations) may also help.

Response to Minor Comment 4:

Thank you for the valuable suggestion. In the revised manuscript, we have illustrated GRFs and kinematics of the generated gaits at small, normal, and fast walking speeds in terms of biomechanics. Further, these values are compared to those of humans and discussed.

In terms of neural control, we focused on Electromyogram (EMG) during walking; however, no open-source database that measured the EMG of the eight muscles used in this study existed. The EMG data used in the previous study to compare humans walking was not open source (M Jacquelin Perry, 2010). The EMG data published in open sources, to the best of our knowledge, do not include the muscles used in this study(Michael H Schwartz et al, 2008, Gabriele Bovi et al., 2011, Tiziana Lencioni et al., 2019, Jonathan Camargo et al., 2021) or only measured during stance phase (Tim J. van der Zee et al, 2022). Therefore, we showed muscle activity of the generated gait without comparison with humans.

Results: Generated gaits, Lines 318-320

> Fig 8 illustrates the ground reaction forces (GRF) and kinematics of the generated gait using the optimized functions derived with $A = 10^6$ and those of humans at corresponding speeds [57].

Results: Key reflex circuits for energy-efficient walking over wide range velocity, Lines 389-390

> Fig 16 shows the muscle activations of the model when v_x^{tar} was set to 1.0 m/s.

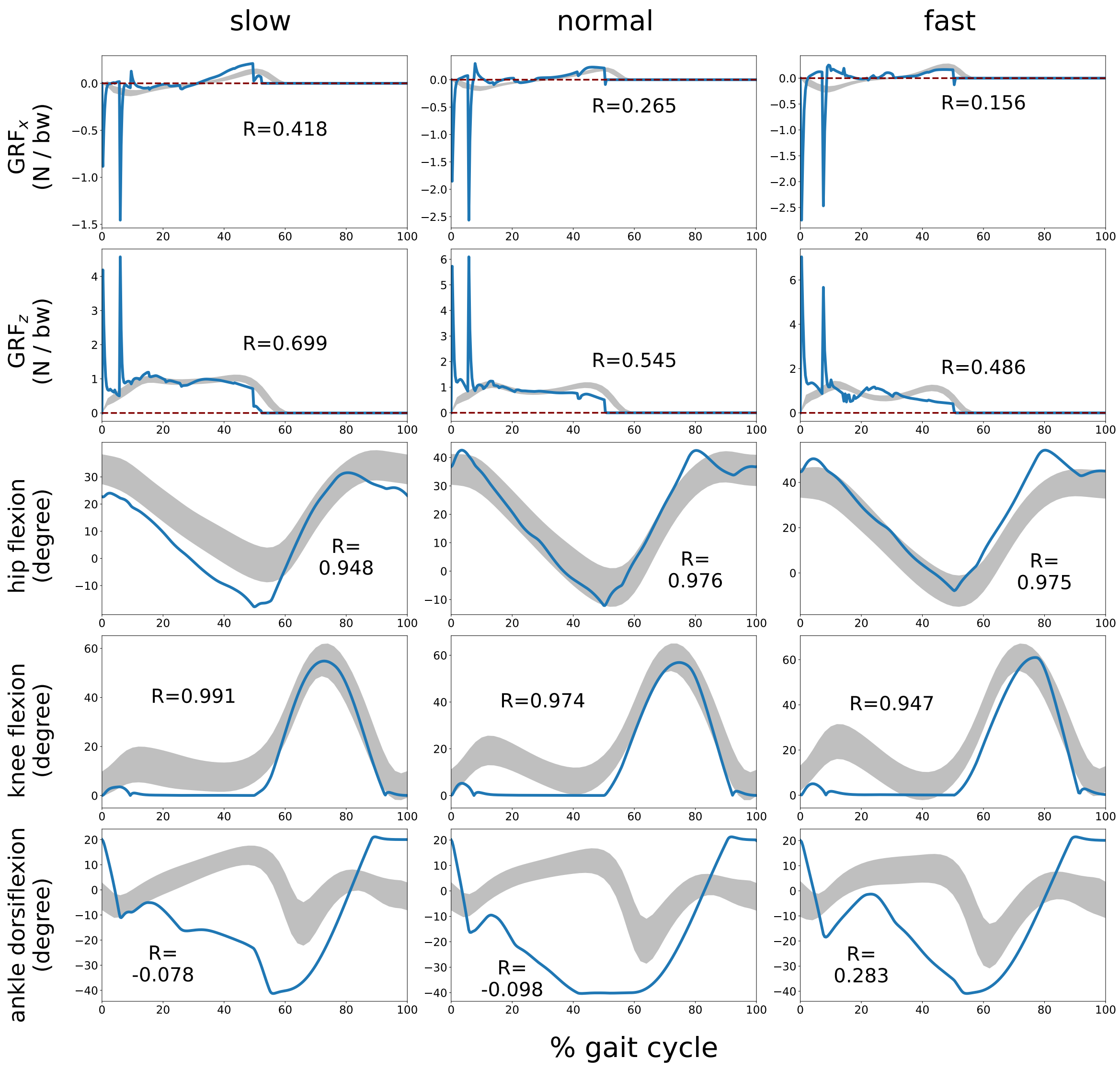

Fig 8.GRFs and kinematics of the generated gait. v_x^{tar} was set to 0.9 (slow), 1.25 (normal), and 1.6 m/s (fast). Blue lines depict the generated gait, while gray lines represent those of humans (mean±1 s.d.) [57]. GRF values are normalized by the body weight. R denotes the cross-correlation value.

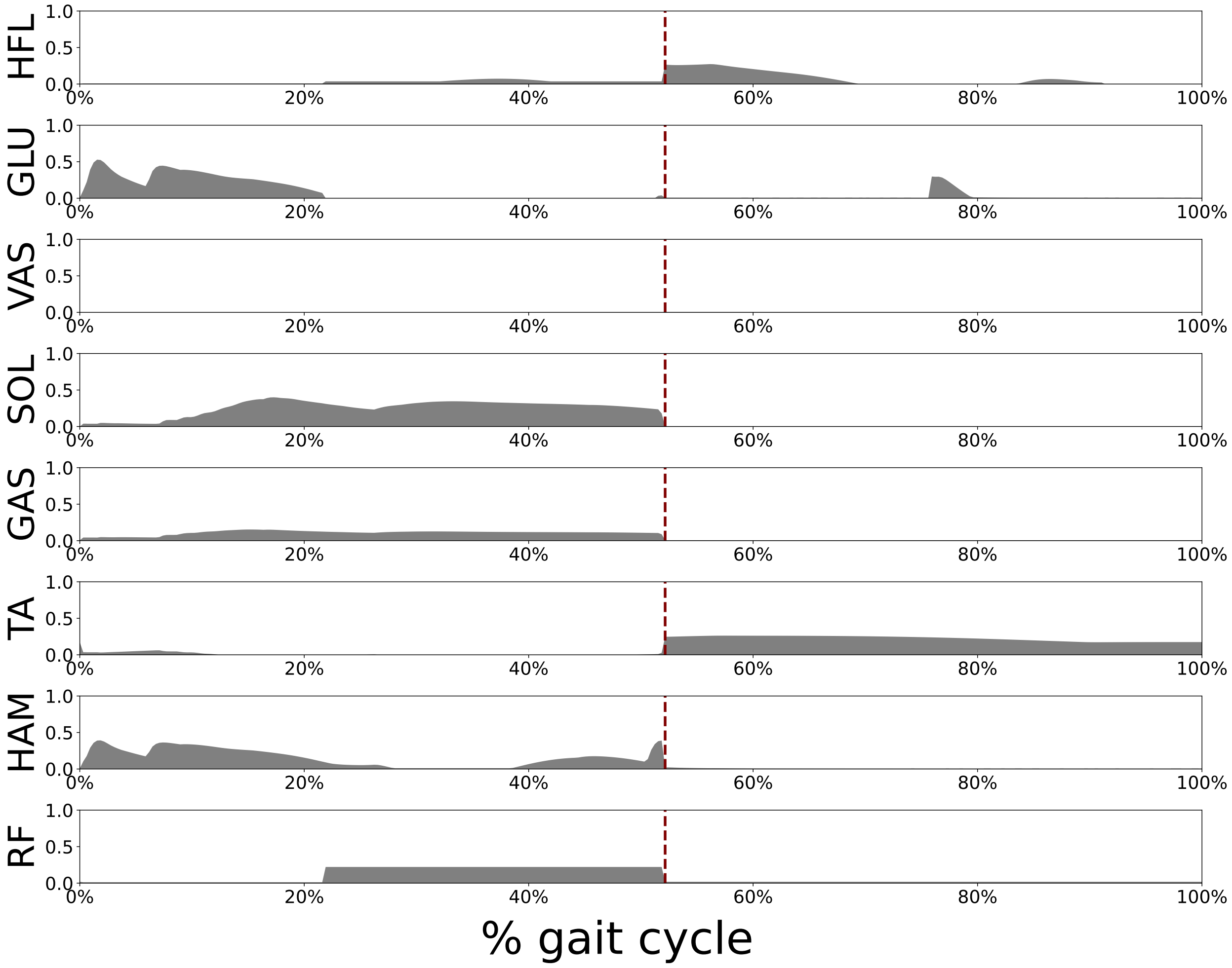

Fig 17. Individual muscle activations. The gait was generated using the optimized functions derived with $A = 10^6$ and v_x^{tar} was set to 1.0 m/s. The red bar indicates the timing of the transition from stance to swing.

Discussion, Lines 446-461

The generated walking deviates from human walking in some aspects. Overshoots and undershoots were observed in the measured GRFs (Fig 8) and time evolution of the walking velocities (Fig 9). In the GRF profiles, there are two undershoots in GRF_x and two overshoots in GRF_z. The first occurred at heel strike and the second occurred at toe-off of the contralateral leg. In the time evolution of the walking velocities, we observed overshoots at toe-off and undershoots at heel strike. These overshoots and undershoots can potentially be mitigated by reducing the simulation timestep [42]. However, employing a smaller timestep leads to increased computational cost. The kinematics of the generated gaits were compared to that of humans as shown in Fig 8. At knee joints, the model straightened the knee earlier in the stance phase compared to humans. This strategy is known to generate more efficient solutions in gait optimization [29]. At ankle joints, the cross-correlation values of ankle dorsiflexion were very low. The previous study also showed low cross-correlation values at ankle joints when optimized to minimize energetic cost [29]. More complex cost function [28] and more human-like body structures [33,34] may be needed to generate human-like ankle joint kinematics.

Minor Comment 5

What is the physiological basis for such a discretized, phase-dependent switching of control modes (L129-135)?

Response to Minor Comment 5:

Thank you for the comment. The signals from the mechanoreceptors in the foot are used for the control of phase transitions in humans (Geyer et al., 2010). Similarly, the control law switches depending on whether the leg is in the stance or swing in this reflex-based controller. We added the following sentences in the supplementary material.

Supplementary material: Reflex-based control, Lines 4-6

Humans use the signals from the mechanoreceptors in the foot for the control of phase transitions [2]. Similarly, the control law switches depending on whether the leg is in the stance or swing phase, and additional stimulation is added during the late stance phase and late swing phase.

Minor Comment 6

While CMA-ES is indeed suitable for global search in nonlinear and non-convex space, how would you validate the algorithm converged to a global minimum? More generally, do you have any insights as to what the landscape of the solution space looks like (e.g., redundant solutions with many local minima)?

Response to Minor Comment 6:

Thank you for the comment. The following figure illustrates the convergence curve for the different target velocities, v_x^{tar}, in the optimization process (added to the Supplementary material).

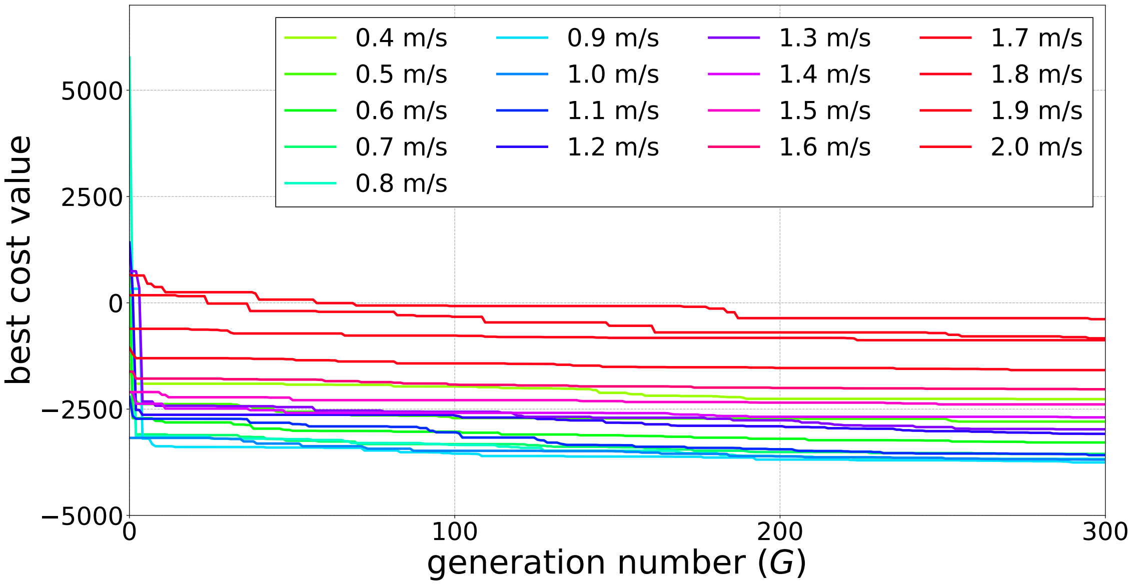

Figure 1: **Convergence curve** The vertical axis indicates the calculated minimum value of the cost function, f (Eq.(7)).

We can observe that the optimization was converged. However, because there are as many as 56 control parameters in the controller, there are many solutions that generate gaits, some of which are unnatural and inefficient local minima (Song et al., 2021). To validate that the optimization was converged to the global minima, we have compared GRFs and kinematics of the generated gaits at small, normal, and fast walking speeds to those of humans as described in minor comment 4. Then, we discussed the calculated cross-correlation values, R, between the generated gaits and humans.

Discussion, Lines 446-461

The generated walking deviates from human walking in some aspects. Overshoots and undershoots were observed in the measured GRFs (Fig 8) and time evolution of the walking velocities (Fig 9). In the GRF profiles, there are two undershoots in GRF_x and two overshoots in GRF_z. The first occurred at heel strike and the second occurred at toe-off of the contralateral leg. In the time evolution of the walking velocities, we observed overshoots at toe-off and undershoots at heel strike. These overshoots and undershoots can potentially be mitigated by reducing the simulation timestep [42]. However, employing a smaller timestep leads to increased computational cost. The kinematics of the generated gaits were compared to that of humans as shown in Fig 8. At knee joints, the model straightened the knee earlier in the stance phase compared to humans. This strategy is known to generate more efficient solutions in gait optimization [29]. At ankle joints, the cross-correlation values of ankle dorsiflexion were very low. The previous study also showed low cross-correlation values at ankle joints when optimized to minimize energetic cost [29]. More complex cost function [28] and more human-like body structures [33,34] may be needed to generate human-like ankle joint kinematics.

Minor Comment 7

The graphical presentation of the scheme for PWLS in the Supplementary material (video) was actually very helpful. If the authors intend to emphasize this, which is the novel contribution of this work, should they consider presenting this in the main manuscript?

Response to Minor Comment 7:

Thank you for the valuable suggestion. We have incorporated a new subsection (**Simple application example of PWLS**) that presents a simple application example of PWLS as follows.

Modulation of control parameters: Simple application example of PWLS, Lines 280-290

In this section, we explain how PWLS computes polynomials, based on a simple illustrative example. We assume that there is a dataset of a control parameter y_i, comprising a total of 18×9 data points, each evaluated through the CoT as depicted in Fig 5. The horizontal axis indicates the generated walking velocity and the vertical axis indicates the control parameter value. The black-colored data points correspond to a lower CoT value of 0.5 (more efficient), while the grey-colored data points correspond to a higher CoT value of 1.0 (less efficient). Fig 5 illustrates polynomials calculated through PWLS. It can be found that the polynomials pass close to the data points with highly evaluated data points with larger A, which determines the strength of the bias toward highly-evaluated data points (Eq.(41)). Thus, by assigning a greater weight to higher-performing data points, walking energy efficiency can be reinforced via regression.

We hope that the revised manuscript has sufficiently improved over the previous version.

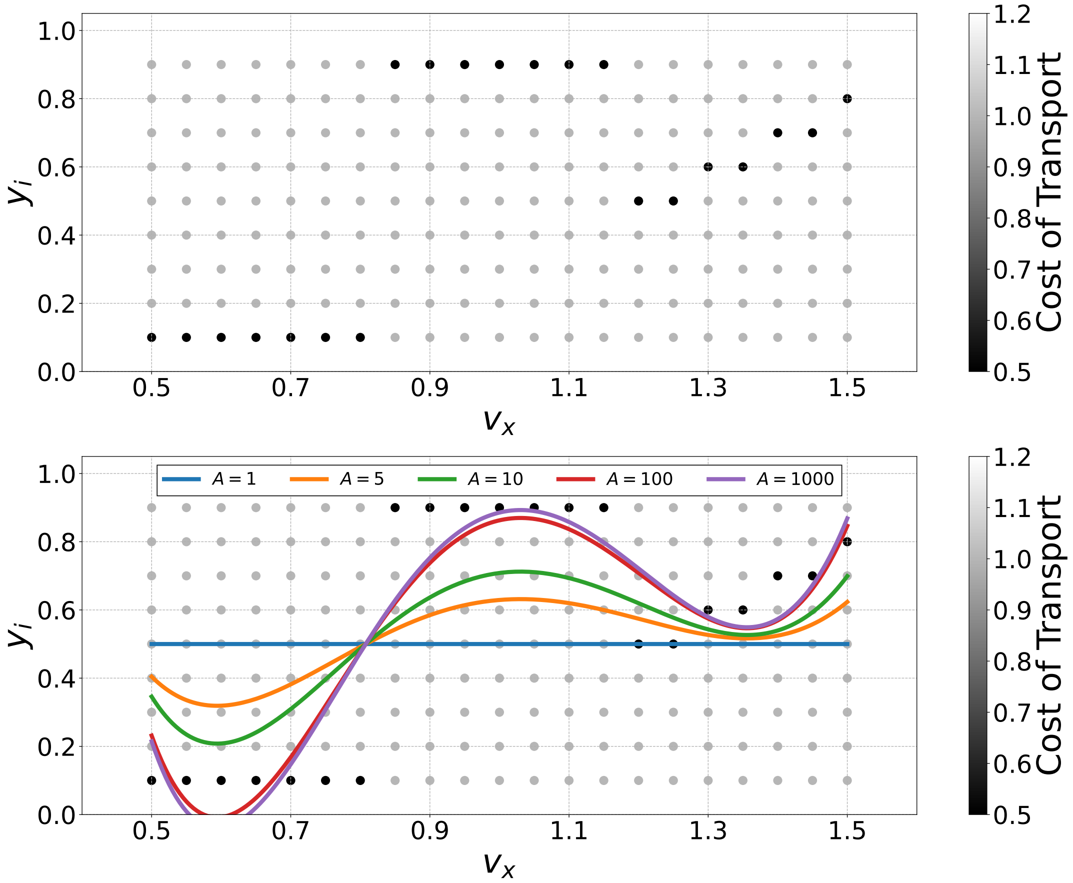

Fig 4. The regression curve derived through PWLS with different A values. Upper: prepared dataset, which consists of 18×9 data points, each evaluated through the CoT. The black-colored data points denote a lower CoT value of 0.5 (efficient), whereas the gray-colored data points denote a higher CoT value of 1.0 (inefficient). Lower: calculated regression curves, with a polynomial degree set to 6.

Comments from Reviewer #3

Red font: Reviewer's comments
Black font: Authors' comments
Blue font: Revised or newly added sentences in the revised paper

Major Comment 1

The authors identify nine key control parameters for walking based on a 2D simulation model. However, the method of identification could be made more compelling. Please discuss whether these results are generalizable and uninfluenced by your preset model parameters (e.g., spring and damper constants of joints, sensory time delay, and weight coefficients in the objective cost function).

Response to Major Comment 1:

We appreciate the reviewer's careful review of our manuscript. The reviewer's comments were very helpful and provided us with a better perspective of our study. Through the additional experiments, we have demonstrated our conclusion and contribution to the field related to human biomechanics, "minimizing stimulation to the hip flexor (HFL) during the swing phase, while ensuring sufficient hip swing to prevent falls. This is achieved through the coordinated activity between the HFL and the hamstrings (HAM) during the swing phase, which is a crucial aspect for maintaining energy efficiency in reflex-based walking control across a wide range of velocities." We have also confirmed that these facts are reliable and robust regardless of the parameters used, i.e., leg length, sensory time delay, and weight coefficients in the objective cost function.

Results: Identifying factors essential for improving energy efficiency, Lines 349-363

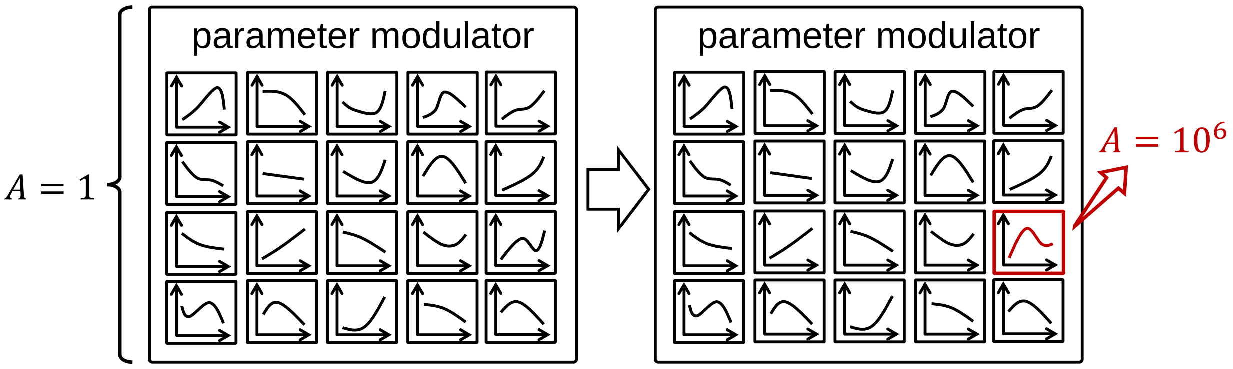

Fig 11. Illustration of the replacement of the optimized function. Left: Before the replacement, all functions were derived with $A = 1$. Right: Only the target control parameter function (red) was replaced with $A = 10^6$. Functions with other parameters were not replaced.

In this study, we aim to identify the key reflex circuits that influence energy-efficient walking across a wide range of velocities. First, to identify the essential factors for improved energy efficiency, we substituted the optimized function of each control parameter that derived at $A = 10^6$, which achieved more energy-efficient walking over a wide range of velocities, in the gait with $A = 1$ (a baseline). Fig 11 illustrates this approach.

Fig 12 illustrates the absolute change in percentage of the $\int$ CoT values, $\Delta|\int \text{CoT}|$, to the generated gait with $A = 1$. From the results, we found that G_{HFL}, l^{tar}_{HAM} and θ^{tar}_{t} are the three parameters that changed $\Delta|\int \text{CoT}|$ value by more than 0.5%. This result suggests that reflex circuits that involve these parameters may have a significant impact on energy-efficient walking across a wide range of velocities. The two length-feedback reflex circuits, $G_{HFL}(l_{HFL} - l^{tar}_{HFL})$ and $G_{HAM\_HFL}(l_{HAM} - l^{tar}_{HAM\_HFL})$, is the primary circuits involved in these parameters, G_{HFL} and l^{tar}_{HAM}: both reflex circuits stimulate HFL muscle during the swing phase. θ^{tar}_{t}, which is the reference angle of the torso, changed $\Delta|\int \text{CoT}|$ value of the third. This parameter was not related to a specific reflex circuit but was shared with several reflex circuits.

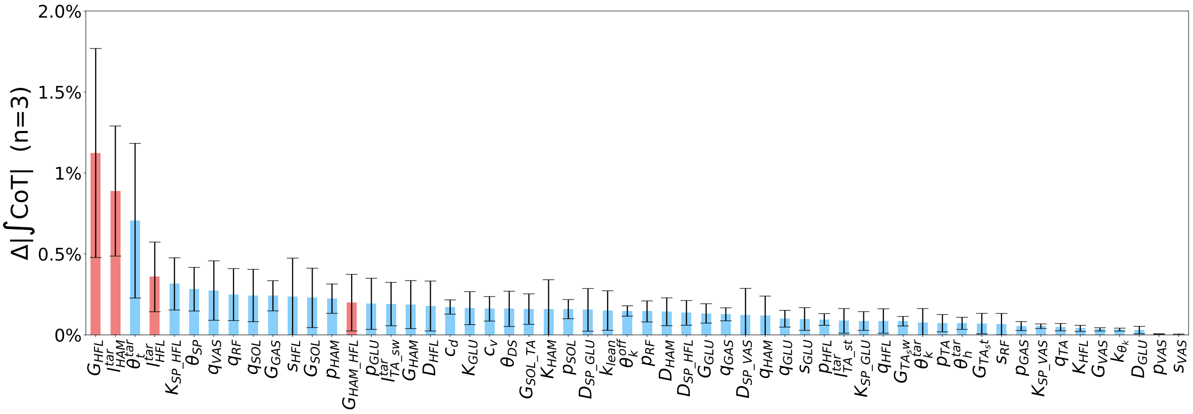

Fig 12. Absolute change in $\int$CoT values when the optimized function of each parameter was substituted to that derived with $A = 10^6$ in the generated gait with $A = 1$ ($n = 3$). The red bars represent control parameters associated with the reflex circuits under focus.

Results: Key reflex circuits for energy-efficient walking over wide range velocity, Lines 366-395

The aforementioned results suggest that $G_{HFL}(l_{HFL} - l^{tar}_{HFL})$ and $G_{HAM\_HFL}(l_{HAM} - l^{tar}_{HAM\_HFL})$ could be the parts of the essential reflex circuits on the wide-range velocity walking with maintaining energy efficiency. Therefore, we proceeded to investigate how modulating these reflex circuits affects the energy efficiency of the gait. We measured the change in $\int$CoT values when the optimized functions of the control parameters associated with $G_{HFL}(l_{HFL} - l^{tar}_{HFL})$ (reflex circuit 1) and $G_{HAM\_HFL}(l_{HAM} - l^{tar}_{HAM\_HFL})$ (reflex circuit 2) were replaced with those derived with $A = 10^6$ in the generated gait with $A = 1$ (a baseline), similar to Fig 11, and the result is shown in Fig 14. The cyan and olive bar represents the relative $\int$CoT value when we replaced only the optimized functions associated with reflex circuits 1 and 2, respectively. The pink bar represents the relative $\int$CoT value when we replaced the optimized function of both reflex circuits. The results indicated that the modulation of only these two reflex circuits resulted in a comparable or even more substantial reduction in $\int$CoT value compared to the modulation of all 56 parameters for the case of $A = 10^6$ shown in the purple bar. This finding strongly suggests that the two length-feedback reflex circuits, $G_{HFL}(l_{HFL} - l^{tar}_{HFL})$ and $G_{HAM\_HFL}(l_{HAM} - l^{tar}_{HAM\_HFL})$, are the key reflex circuits of the energy efficiency in the reflex-based bipedal walking control.

To elucidate the mechanism behind the improved energy efficiency, we examined the change in energy consumption by individual muscles, muscle activation patterns, and stimulation signal for the corresponding muscle when we modulated the two identified reflex circuits. Fig 15 shows the energy consumed by individual muscles during a 30 m walk at $v^{tar}_x = 1.0$. We found that energy consumption of the HFL, GLU, and HAM muscles was significantly reduced after reflex circuits 1 and 2 were modulated. Fig 16 shows the muscle activations of the bipedal model for $v^{tar}_x = 1.0$ m/s. HFL muscle was mainly activated in the swing phase while GLU and HAM muscles were activated in the stance phase. Fig 17 illustrates the stimulation signal applied to HFL muscle during the swing phase before (blue line) and after the modulation (pink line) of reflex circuits 1 and 2: the stimulation signal applied to HFL muscle decreased after the modulation of these identified reflex circuits.

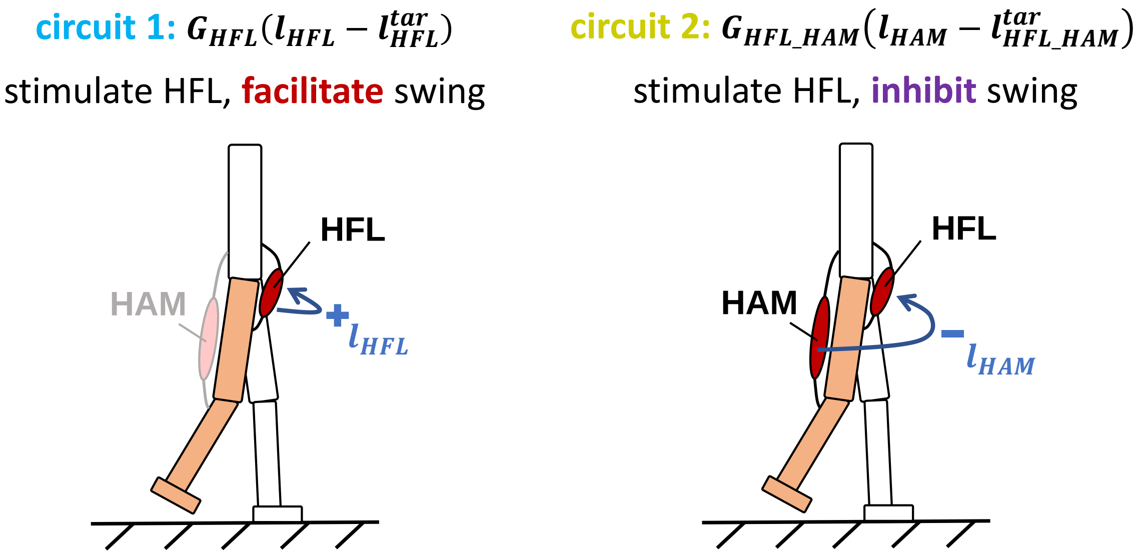

Fig 13. Key reflex circuits for energy-efficient gait. $G_{HFL}(l_{HFL} - l^{tar}_{HFL})$ is the positive length feedback stimulating HFL to swing the leg forward. $G_{HAM\_HFL}(l_{HAM} - l^{tar}_{HAM\_HFL})$ is the negative length feedback inhibiting the HFL proportional to the stretch of the HAM in the swing phase. In these terms, G represents the gain, l represents the length of the muscle, and l^{tar} represents the constant target length. Both reflex circuits are active only in the swing phase.

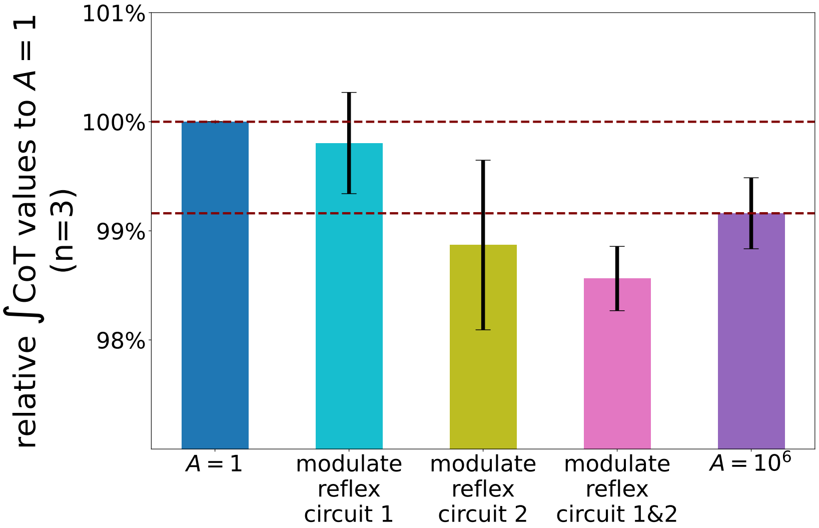

Fig 14. Relative $\int$CoT values to the generated walking with $A = 1$ when modulating identified reflex circuits (n=3). The cyan and olive bar represents the relative $\int$CoT value when only the optimized function of control parameters associated with reflex circuit 1 (i.e. G_{HFL} and l^{tar}_{HFL}) and reflex circuit 2 (i.e. G_{HAM_HFL} and $l^{tar}_{HAM\_HFL}$) were replaced with those derived with $A = 10^6$ in the generated gait with $A = 1$, respectively. The pink bar represents when the optimized function of the control parameters associated with both reflex circuits (i.e., G_{HFL}, l^{tar}_{HFL}, G_{HAM_HFL}, $l^{tar}_{HAM\_HFL}$) were replaced. The purple bar indicates the value in the generated gait using the optimized functions derived with $A = 10^6$. When circuit 2 was modulated, some generated gaits did not follow the specific input v^{tar}_{vel}, with their velocities being changed by more than 0.05 m/s. Data acquired from the target velocity that resulted in these outliers were excluded from the calculation of estimated CoT curves.

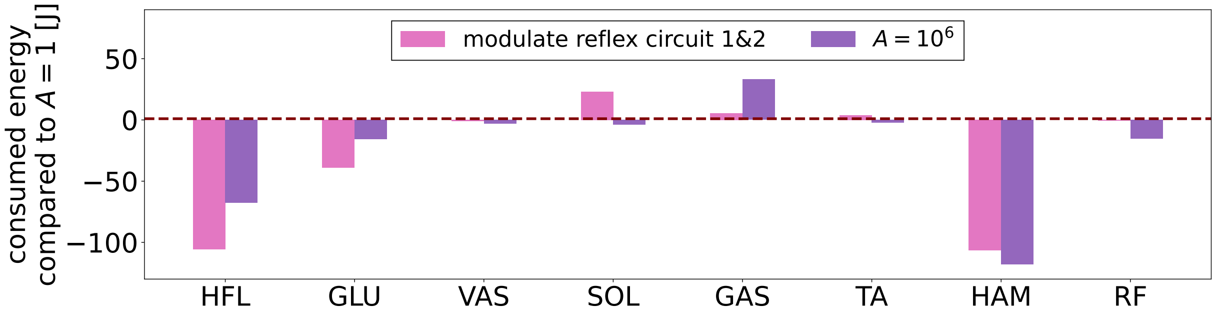

Fig 15. Energy consumed by individual muscles compared to the generated gait with $A = 1$ during a 30 m walk. v_x^{tar} was set to 1.0 m/s. The pink bars represent the values when the optimized functions of the control parameters associated with reflex circuits 1 and 2 are replaced with those derived with $A = 10^6$ in the gait generated with $A = 1$, and the purple bars represent the values in the generated gait with $A = 10^6$. The negative value indicates that the energy consumption at the muscle was reduced compared to the generated gait with $A = 1$, while the positive value indicates increased energy consumption.

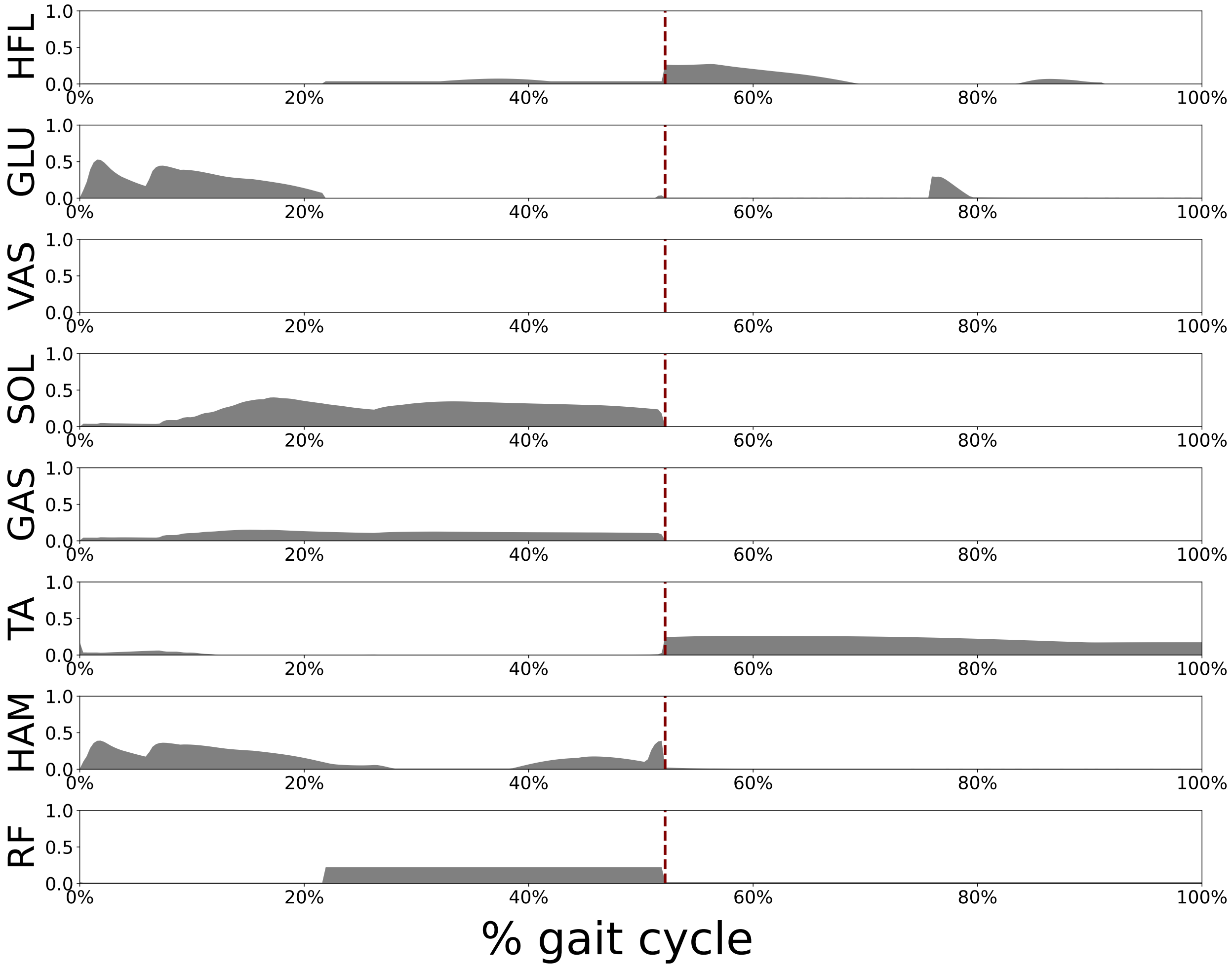

Fig 16. Individual muscle activations. The gait was generated using the optimized functions derived with $A = 10^6$ and v_x^{tar} was set to 1.0 m/s. The red bar indicates the timing of the transition from stance to swing.

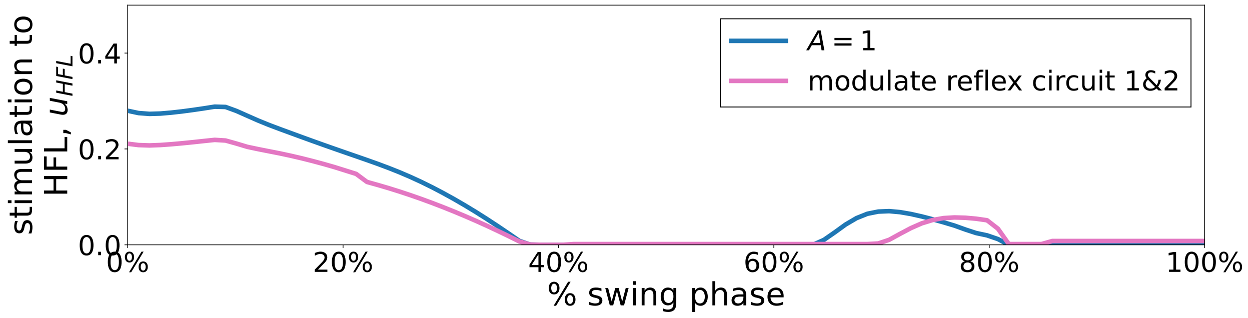

Stimulation to the HFL in the swing phase before and after modulating reflex circuit 1&2. v_x^{tar} was set to 1.0 m/s. The blue and pink lines are the stimulation to the HFL, which does not drop below 0, at generated gait with $A = 1$ and after the optimized function of the control parameters associated with reflex circuit 1 and 2 are replaced with those derived with $A = 10^6$, respectively.

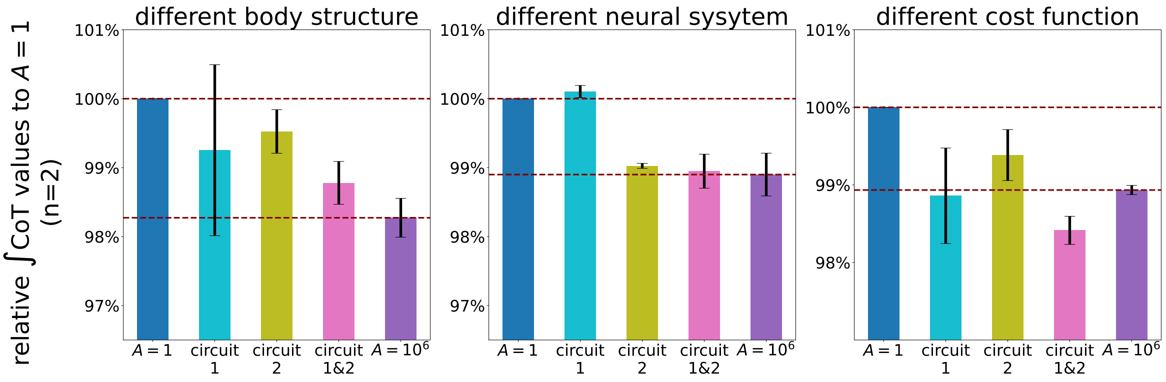

Fig 18. Relative $\int$ CoT values to the generated walking with $A = 1$ under different setting parameters (n=2). In a different body structure setting, the segment lengths of the model were shortened by 20 %. In a different neural system setting, the time delay of the sensory information to the controller was doubled. In this setting, stable gaits were not generated for $A > 100$. In a different cost function setting, the weight coefficients in the objective function (Eq.(31)) were changed to $\alpha_E = 2500$, $\alpha_v = 5$, and $\alpha_t = 0$.

Results, Robustness to parameter changes, Lines 397-409

Under various parameter conditions, leg length, sensory time delay, and weight coefficients for the cost function, we have validated the reliability and robustness of the identified two reflex circuits concerning their impact on energy-efficient walking over a wide range of velocities:

- shorten segment length by 20% (different body structure)
- double the time delay of sensory information transmission to the controller (different neural system)
- change the weight coefficients in the objective cost function (Eq.(31)), $\alpha_E = 2500$, $\alpha_v = 5$, and $\alpha_t = 0$ (different cost function)

Fig 18 illustrates the relative $\int$ CoT values to the baseline ($A = 1$) under various conditions. We found that modulating reflex circuits 1 and 2 resulted in decreased $\int$ CoT values, comparable to the result for all 56 parameter modulation (generated gaits can be seen in S1 Video).

Major Comment 2

Furthermore, could these findings be extended to a 3D context? As noted in the paper, such an extension could have practical applications.

Response to Major Comment 2:

Thank you for the valuable suggestion. As the reviewer pointed out, the extension to 3D context is essential for practical engineering applications. We expect that by applying the findings of existing research that achieved the generation of walking in a 3D musculoskeletal model (Song et al., Journal of Physiology, 2015), it will be possible to extend to the 3D context, which is one of our future plans. We have included the following sentences to the **Discussion**.

Discussion, Lines 472-474

Moreover, the bipedal model motion was constrained to the sagittal plane. Hence, the extension of the motion into three dimensions is another important future work to apply the controller to engineering applications.

Major Comment 3

Figure 7C indicates that for varying A, the values of many control parameters remain closely matched. When the optimized function of certain parameters is replaced with the one derived at A=800, the shift in energy efficiency is minimal (Fig 9). I feel it is not convincing to deduce that these parameters are less critical.

Response to Major Comment 3:

Thank you for the insightful comment. As thoroughly explained in the response to major comment 1, we have conducted additional experiments to provide a more convincing explanation in the revised manuscript. First, we focused on the baseline $A = 1$ and the condition $A = 10^6$, which achieved high energy-efficient walking over a wide range of velocities. We then examined how energy efficiency changed by replacing one of the 56 optimized functions with that derived with $A = 10^6$ in the baseline gait ($A = 1$) as shown in Fig 12. This led to the extraction of two primary factors (parameters) G_{HFL} and l^{tar}_{HAM}.

Second, based on the results, we then identified the reflex circuits involved in the two essential parameters (Fig 13). Subsequently, we followed up on how modifications to the identified reflex circuits affect the overall energy efficiency (Fig 14). Consequently, we confirmed that the modification of the two reflex circuits resulted in a change in energy efficiency comparable to that obtained by changing all functions to the condition $A = 10^6$, suggesting that the two reflex circuits contributed significantly to improving energy efficiency over a wide velocity range. Therefore, we believe that the revised manuscript now provides sufficiently convincing results of the key reflex circuits for energy efficiency.

Results, Identifying factors essential for improving energy efficiency, Lines 349-363

Please see the response to major comment 1.

Results, Key reflex circuits for energy-efficient walking over wide range velocity, Lines 366-395

Please see the response to major comment 1.

Results, Robustness to parameter changes, Lines 397-409

Please see the response to major comment 1.

Minor Comment 1

Why is the chosen speed range only between 0.5 to 1.5 m/s? The typical human walking speed exceeds this range. Considering the emphasis on "wide range" in the title, the chosen range appears somewhat restrictive.

Response to Minor Comment 1:

We appreciate your constructive comment. In the revised manuscript, we have re-conducted the simulation using the revised model as described in the introduction of this response letter. The range of velocity in newly generated walking gait was from 0.7 to 1.6 m/s, which covers the range of speeds at which humans normally walk (1.0-1.6 m/s).

Minor Comment 2

what is the joint torque function with spring and damper constants? Additionally, is the unit of the damper constant accurately represented? Shouldn't it be represented as Nms/rad instead?

Response to Minor Comment 2:

Thank you for the insightful comment. As the reviewer pointed out, the unit of the damper constants was incorrectly described as Nm/rad instead of Nms/rad. The spring in the joints produces a torque, T, proportional to the difference between the current joint position, θ, and the equilibrium position, θ_{ref}, i.e., $T = k(\theta - \theta_{ref})$, where k is the spring constant. The damper in joints produces friction proportional to the joint angular velocity, i.e., $T = -c\dot{\theta}$, where c is the damper constant. We have revised the manuscript as follows.

Generating walking through reflex-based control: Musculoskeletal model, Lines 79-85

The spring and damper constants for all joints apart from the toe joints are set to 0 Nm/rad and 1 Nms/rad, respectively. The toe joints are set with a spring constant of 30 Nm/rad, which is consistent with [28], and a damping constant of 0 Nms/rad. The spring and damper within the joint generate a torque that is proportional to the distance from the equilibrium position and friction that is proportional to the joint's angular velocity, respectively.

Minor Comment 3

Could you elaborate on the reasons for the toe having distinct parameters and the rationale behind choosing the value 30?

Response to Minor Comment 3:

Thank you for the insightful comment. Our model was identical to those used in the existing study (Wang et al., 2012), and we used the same values for the parameters, in which the spring constant in the toe joint was 30 Nm/rad.

Generating walking through reflex-based control, Musculoskeletal model, Lines 79-82

The spring and damper constants for all joints apart from the toe joints are set to 0 Nm/rad and 1 Nms/rad, respectively. The toe joints are set with a spring constant of 30 Nm/rad, which is consistent with [28], and a damping constant of 0 Nms/rad.

Minor Comment 4

Could you elucidate how the model depicts the interaction between the feet and the ground? Is there potential for sliding? If so, what is the assumed coefficient of friction?

Response to Minor Comment 4:

Thank you for the comment. The stance leg is not fixed to the ground,; thus, there is potential for sliding. We set the sliding friction coefficient between the feet and the ground at a relatively high value, 2.5. Then, sliding is unlikely to happen. We added the following sentences to the manuscript.

Generating walking through reflex-based control, Musculoskeletal model, Lines 85-86

We set the sliding friction coefficient between the feet and the ground at a relatively high value, 2.5. Then, sliding is unlikely to happen.

Minor Comment 5

Could you provide a more thorough explanation of equations 11-19 and 24-32? Where do they come from? What's the meaning of each term?

Response to Minor Comment 5:

Thank you for the comment. Eqs. 11–19 and Eqs. 24–32 of the previous manuscript comprises three primary terms; force feedback term, length feedback term, and muscle-driven PD control term. Eqs. 11–19 represent the reflex control in each muscle during the stance phase. In the stance phase, force feedback, $G_i\tilde{F}_i(t-\Delta t_i)$, at GLU, VAS, and SOL generate compliant leg behavior. Length feedback at TA, $\max\{0, G_{TA\_st}(l_{TA\_st}(t-\Delta t_l) - l^{tar}_{TA\_st})$, prevents overextension of the ankle joint, which is suppressed by the force feedback from the SOL, $G_{SOL\_TA}\tilde{F}_{SOL}(t-\Delta t_l)$. Force feedback at GAS, $G_{GAS}\tilde{F}_{GAS}(t-\Delta t_l)$, contributes to push-off and prevents overextension of the knee joint. Muscle-driven PD controls at HFL, GLU, and HAM, $\max\{0, K_i(\theta^{tar}_j - \theta_j(t-\Delta t_i)) \pm D_i\dot{\theta}_j(t-\Delta t_i)\}$, balance the torso. The term, f_{VAS}, prevents knee hyperextensions during the mid-stance phase. Stimulation of the VAS is suppressed when the knee joint angle, θ_k, exceeds the threshold angle, θ^{off}_k.

Eqs. 24–32 represent the reflex control in each muscle during the swing phase. In the swing phase, length feedback at HFL, $\max\{0, G_{HFL}(l_{HFL}(t-\Delta t_s) - l^{tar}_{HFL})\}$, facilitates leg swing, which is suppressed by length feedback from HAM, $\max\{0, G_{HAM\_HFL}(l_{HAM}(t-\Delta t_s) - l^{tar}_{HAM})\}$. Force feedback at GLU and HAM, $G_i\tilde{F}_i(t-\Delta t_i)$, apply braking force to the swing leg. Length feedback at TA, $\max\{0, G_{TA\_sw}(l_{TA}(t-\Delta t_l) - l^{tar}_{TA\_sw})\}$, raises the toes to create clearance between the feet and the ground. The term f_{HFL} stimulates the HFL according to the current torso angle, θ_t, and torso target angle, θ^{tar}_t, as the required protraction speed relies on the torso lean.

The reflex-based controller employed in this study is identical in principle to that of Wang et al (Wang et al., 2012). We did not make ad-hoc adjustments for the basic reflex-based control model such that the simulation would work well. Therefore, these equations all come from this previous work.

In the revised manuscript, since the reflex-based controller is identical to the previous work, we have described the overview of the controller in the main manuscript and explained the detailed equations in the Supplementary material to clarify and highlight the novel contribution of this work, as suggested by the other reviewer (Reviewer #2, Minor Comment 2).

Generating walking through reflex-based control, Reflex-based control, Lines 114-151

The reflex-based controller employed in this study is identical in principle to that of Wang et al. [28]. We did not make any ad-hoc adjustments to the basic reflex-based control model to ensure optimal simulation performance. The controller computes the muscle stimulation, denoted as u_i, for each muscle, i, by incorporating sensory feedback with a time delay of Δt as input. The control law switches depending on whether the leg is in the stance or swing phase. Moreover, additional stimulation is introduced during the late stance and late swing phases. The control system comprises three primary functions: force feedback, length feedback, and muscle-driven proportional derivative (PD) control.

- Force feedback:
  The force feedback law returns the stimulation signal, u^F_i, in response to the actuator force, F_i. In humans, the signals of the F_i arise from Golgi tendon organs and are carried by type Ib afferents to the spinal cord. u^F_i is calculated as follows:

$$u^F_i = G_i\tilde{F}_i(t-\Delta t_i), \tag{65}$$

$$\tilde{F}_i(t - \Delta t_i) = \frac{F_i(t - \Delta t_i)}{F_i^0}, \tag{66}$$

where the gain, G_i, is the positive control parameter, and $\tilde{F}_i(t - \Delta t_i)$ is the scaled actuator force, (i.e., $\tilde{F}_i = F_i/F_i^0$), which includes the sensory time delay, Δt_i.

- Length feedback
  Through length feedback, we calculate the stimulation signal, denoted as u_i^l, corresponding to the length of the muscle actuator, l_i. This function models the stretch reflex of the muscle spindle. u_i^l is calculated as follows:

$$u_i^l = \max\{0, G_i(l_i(t - \Delta t_i) - l_i^{tar})\}, \tag{67}$$

  where the target length, l_i^{tar}, and gain, G_i, are positive control parameters.

- Muscle-driven PD control
  The muscle-driven PD control generates the stimulation necessary to move joint θ_j (Fig 1B) to the target angle, θ_j^{tar}. This function can be interpreted as a polysynaptic reflex that is mediated by the joint position afferent input from group III fibers and descending signals of the target joint angles from the supraspinal. The muscle-driven PD control laws are employed to stabilize the generated gait by the hip muscles during the stance phase to balance the torso and during stance preparation to prepare for ground contact (described in S1 Appendix). For muscle actuator, i, which applies torque to joint θ_j in the positive direction, $u_i^{\theta_j}$ is defined as follows:

$$u_i^{\theta_j} = \max\{0, K_i(\theta_j^{tar} - \theta_j(t - \Delta t_i)) - D_i\dot{\theta}_j(t - \Delta t_i)\}. \tag{68}$$

  Conversely, for muscle actuator i, which applies torque in the negative direction, u_i^{θ} is defined as follows:

$$u_i^{\theta} = \max\{0, K_i(\theta_j(t - \Delta t_i) - \theta_j^{tar}) + D_i\dot{\theta}_j(t - \Delta t_i)\}, \tag{69}$$

  where the proportional gain, K_i, and derivative gain, D_i, are positive control parameters.

Fig 2 illustrates the reflexes in the stance phase and swing phase. Please see S1 Appendix or the original paper [28] for more details.

Supplementary material: Reflex-based control, Lines 8-38

Stance Phase

During the stance phase, the stimulation, u_i, to each muscle actuator is calculated as follows:

$$u_{SOL} = p_{SOL} + G_{SOL}\tilde{F}_{SOL}(t - \Delta t_l), \tag{70}$$

$$u_{TA} = p_{TA} + \max\{0, G_{TA\_st}(l_{TA}(t - \Delta t_l) - l_{TA\_st}^{tar}))\} - G_{SOL\_TA}\tilde{F}_{SOL}(t - \Delta t_l), \tag{71}$$

$$u_{GAS} = p_{GAS} + G_{GAS}\tilde{F}_{GAS}(t - \Delta t_l), \tag{72}$$

$$u_{VAS} = p_{VAS} + G_{VAS}\tilde{F}_{VAS}(t - \Delta t_m) + f_{VAS}, \tag{73}$$

$$f_{VAS} = \begin{cases} 0 & (\theta_k < \theta_k^{off}) \\ k_{\theta_k}\{\theta_k(t - \Delta t_{VAS}) - \theta_k^{off}\} & (\theta_k \geq \theta_k^{off}) \end{cases}, \tag{74}$$

$$u_{HAM} = p_{HAM} + \max\{0, K_{HAM}(\theta_t(t - \Delta t_s) - \theta_t^{tar}) + D_{HAM}\dot{\theta}_t\}, \tag{75}$$

$$u_{RF} = p_{RF}, \tag{76}$$

$$u_{GLU} = p_{GLU} + \max\{0, K_{GLU}(\theta_t(t - \Delta t_s) - \theta_t^{tar}) + D_{GLU}\dot{\theta}_t\}, \tag{77}$$

$$u_{HFL} = p_{HFL} + \max\{0, K_{HFL}(\theta_t^{tar} - \theta_t(t - \Delta t_s)) + D_{HFL}\dot{\theta}_t\}, \tag{78}$$

where p_i are positive constant control parameters that are approximate to zero. The time delays, Δt_l, Δt_m, and Δt_s, are set to 20, 10, and 5 ms, respectively. The shorter transmission pathway to the spinal cord results in smaller time delays; therefore, the muscles near the hip joint have minimal time delays. Positive force feedback at GLU, VAS, and SOL contributes to compliant leg behavior. When the gains, denoted as G, are set higher, the stance leg emulates an inverted pendulum model. Conversely, when the gains are set at smaller values, it behaves as a spring-inverted pendulum model [3]. Positive length feedback at TA prevents overextension of the ankle joint. This action is suppressed by negative force feedback from the SOL, which serves to extend the ankle joint during

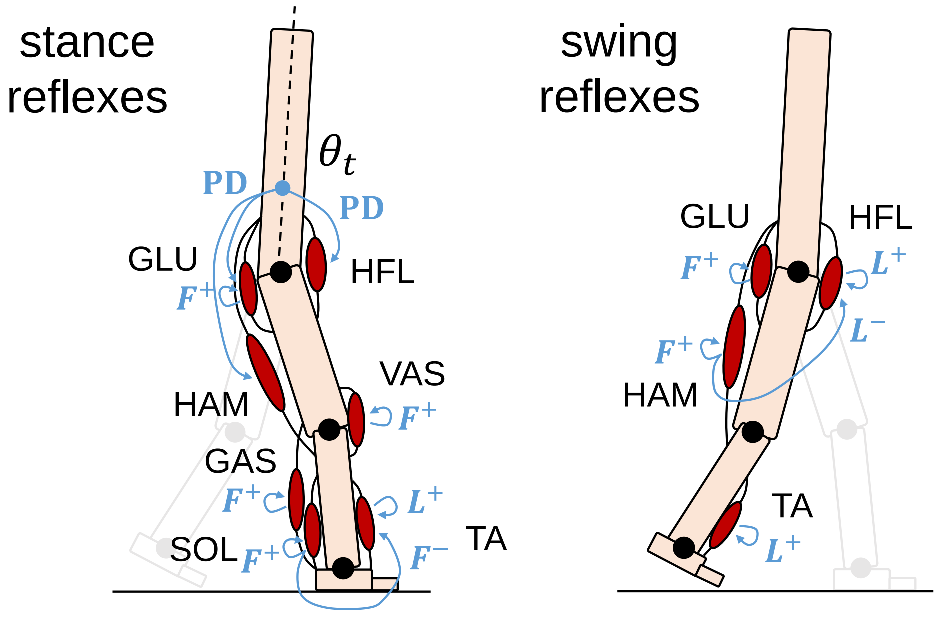

Fig 2. Key reflexes in the stance and swing phase. F represents force feedback, L represents length feedback, and PD represents muscle-driven PD control. $+$ and $-$ denote positive and negative feedback, respectively. In the stance phase, F^+ at GLU, VAS, and SOL generate compliant leg behavior. L^+ at TA prevents overextension of the ankle joint, which is suppressed by the F^- from the SOL. F^+ at GAS contributes to push-off and prevents overextension of the knee joint. Muscle-driven PD controls at HFL, GLU, and HAM balance the torso. In the swing phase, L^+ facilitates leg swing, which is suppressed by L^- from HAM. F^+ at GLU and HAM apply braking force to the swing leg. L^+ at TA raises the toes to create clearance between the feet and the ground.

a push-off. Positive force feedback at GAS contributes to push-off and prevents overextension of the knee joint. Muscles around the hip joint, namely HFL, GLU, and HAM, function together to maintain the reference lean angle θ_t^{tar} through muscle-driven PD controls, thus stabilizing the torso. The term, f_{VAS}, prevents knee hyperextensions during the mid-stance phase. Stimulation of the VAS is suppressed when the knee joint angle, θ_k, exceeds the threshold angle, θ_k^{off}. During the swing initiation phase, when the hip extension angle, θ_h, exceeds θ_{SI}, as depicted in Fig S1A, constant stimulation values s_i that are greater than zero are added to the VAS, RF, GLU, and HFL to encourage swing initiation [4].

$$u_{VAS} \leftarrow u_{VAS} - s_{VAS}, \tag{79}$$
$$u_{RF} \leftarrow u_{RF} + s_{RF}, \tag{80}$$
$$u_{GLU} \leftarrow u_{GLU} - s_{GLU}, \tag{81}$$
$$u_{HFL} \leftarrow u_{HFL} + s_{HFL}. \tag{82}$$

Swing Phase

During the swing phase, the stimulation, u_i, to each muscle actuator is calculated as follows:

$$u_{SOL} = q_{SOL}, \tag{83}$$
$$u_{TA} = q_{TA} + \max\{0, G_{TA\_sw}(l_{TA}(t - \Delta t_l) - l^{tar}_{TA\_sw})\}, \tag{84}$$
$$u_{GAS} = q_{GAS}, \tag{85}$$
$$u_{VAS} = q_{VAS}, \tag{86}$$
$$u_{HAM} = q_{HAM} + G_{HAM}\tilde{F}_{HAM}(t - \Delta t_s), \tag{87}$$
$$u_{RF} = q_{RF}, \tag{88}$$
$$u_{GLU} = q_{GLU} + G_{GLU}\tilde{F}_{GLU}(t - \Delta t_s), \tag{89}$$
$$u_{HFL} = q_{HFL} + \max\{0, G_{HFL}(l_{HFL}(t - \Delta t_s) - l^{tar}_{HFL})\} - \max\{0, G_{HAM\_HFL}(l_{HAM}(t - \Delta t_s) - l^{tar}_{HAM})\} + f_{HFL}, \tag{90}$$
$$f_{HFL} = k_{lean}\{\theta^{tar}_t - \theta_t(t - \Delta t_{HFL})\}, \tag{91}$$

where q_i are positive constant control parameters similar to p_i and also approximate zero. Positive length feedback at HFL facilitates leg swing, which is suppressed by negative length feedback from HAM in the mid-swing phase. The term f_{HFL} stimulates the HFL according to the current torso angle, θ_t, and the torso target angle, θ^{tar}_t. The required protraction speed relies on the torso lean. Positive force feedback at GLU and HAM applies braking force to the swinging leg, transferring some of the angular momentum into leg lowering and retraction. Positive length feedback at TA raises the toes, ensuring sufficient clearance between the feet and the ground. During the stance preparation phase, when the hip flexion angle, θ_h, exceeds θ_{SP}, as depicted in Fig S1A, the swinging leg transitions into stance preparation. Muscle-driven PD controls are integrated into the VAS, GLU, and HFL to produce the desired pose for touchdown in this phase.

$$u_{VAS} \leftarrow u_{VAS} + \max\{0, K_{SP\_VAS}(\theta_k(t - \Delta t_m) - \theta^{tar}_k) + D_{SP\_HFL}\dot{\theta}_k(t - \Delta t_m)\}, \tag{92}$$
$$u_{GLU} \leftarrow u_{GLU} + \max\{0, K_{SP\_GLU}(\theta^{tar}_h - \theta_h(t - \Delta t_m)) + D_{SP\_GLU}\dot{\theta}_h(t - \Delta t_m)\}, \tag{93}$$
$$u_{HFL} \leftarrow u_{HFL} + \max\{0, K_{SP\_HFL}(\theta_h(t - \Delta t_m) - \theta^{tar}_h) + D_{SP\_HFL}\dot{\theta}_h(t - \Delta t_m)\}. \tag{94}$$

To reproduce a stable gait, the target hip angle, θ^{tar}_h, is adjusted according to the balance feedback law (SIMBICON) [5]:

$$\theta^{tar}_h = \theta^{tar}_h - c_d d + c_v v_x, \tag{95}$$

where d represents the horizontal distance from the stance ankle to the hip segment, as depicted in Fig S1B. v_x represents the horizontal velocity of the model, and c_d and c_v are positive constant parameters. This balance feedback law can be interpreted as the supraspinal control that uses sensory information from the vestibular organs.

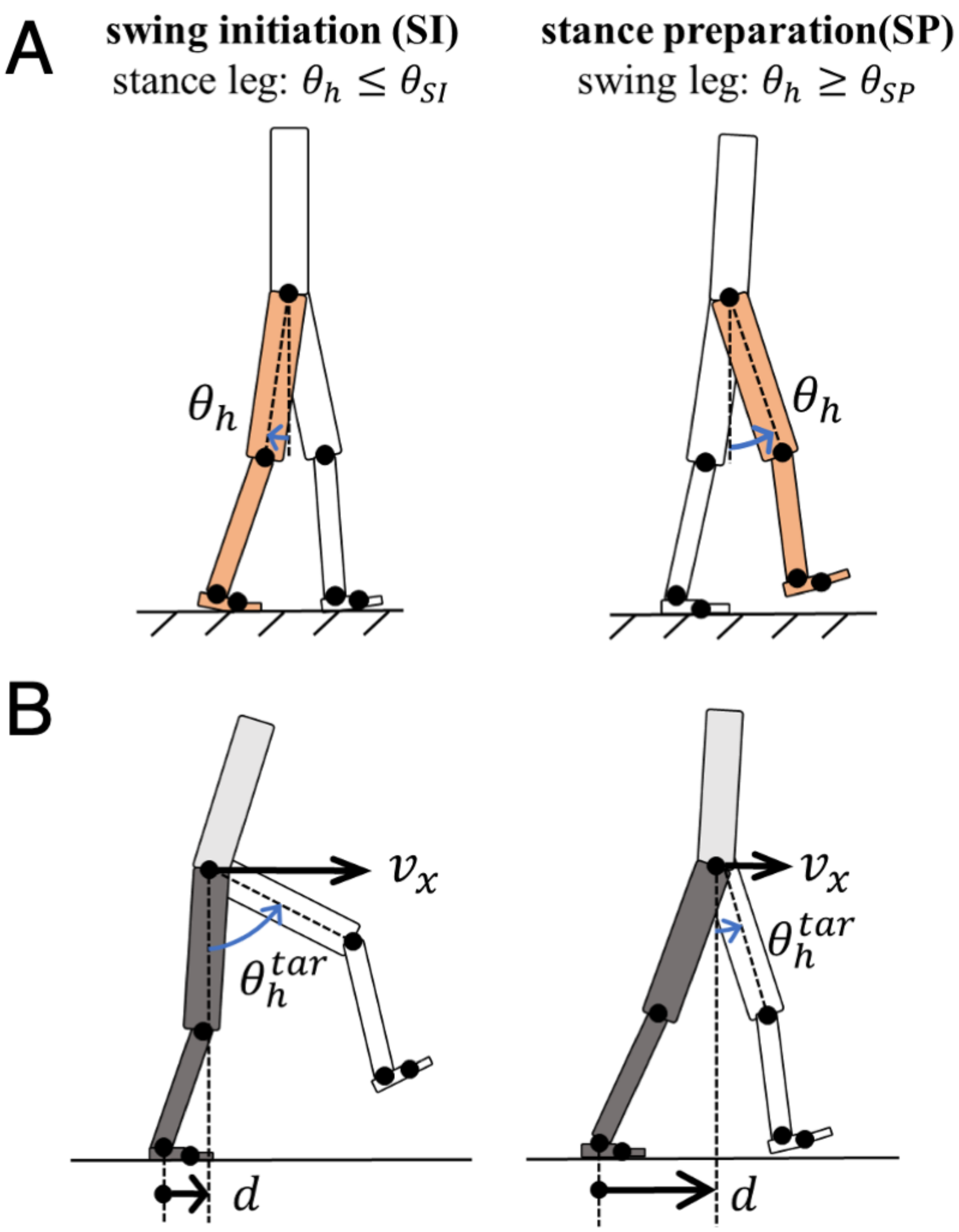

Fig S1: Description of the reflex-based control. A. Transition conditions of swing initiation (SI) and stance preparation (SP) phases. The SI phase commences when the hip extension angle, θ_h, of the stance leg exceeds a predetermined threshold, θ_{SI}. The SP phase commences when the hip flexion angle, θ_h, of the swing leg exceeds the threshold, θ_{SP}. **B. Diagram of the balance feedback law.** The feedback control system dynamically adjusts the target hip angle, θ_h^{tar}, depending on the horizontal distance from the stance ankle to the hip segment, d, and the horizontal model velocity, v_x. For instance, when d is small or v_x is large, θ_h^{tar} is increased, causing the foot to be placed more forward to prevent falling, as depicted on the left. Conversely, when d is large or v_x is small, θ_h^{tar} is decreased.

Minor Comment 6

A clear definition or criteria for "steady walking" is required.

Response to Minor Comment 6:

Thank you for the comment. We defined steady walking as being able to walk more than 30 m without falling. This has been included in the following sentences in the revised manuscript.

Results, Generated gaits, Line 315

Here, we defined steady walking as being able to walk more than 30 m without falling.

Minor Comment 7

Fig. 7: Higher resolution or Scalable Vector Graphics are needed, as Fig. 7c becomes unclear when zoomed in.

Response to Minor Comment 7:

Thank you for the comment. We changed the relevant figures to vector graphics.

Minor Comment 8

Regarding Fig. 7C: The model falls when A = 100,000 for v_x^{tar} =1.2 and 1.5m/s. Why does the curve still include these data points? This raises questions about overfitting and its potential for extension.

Response to Minor Comment 8:

Thank you for the comment. In the previous manuscript, the generated walking velocity for the target velocity $v_x^{tar} = 1.1\,\mathrm{m/s}$ was 1.19 m/s (Fig 3 in the previous supplementary material). Therefore, we can see that the point seems to be plotted close to v_x^{tar}=1.2 m/s. Moreover, we re-conducted the simulation with the revised model described in the introduction of this response letter. Therefore, the result and figures have been updated from the previous manuscript.

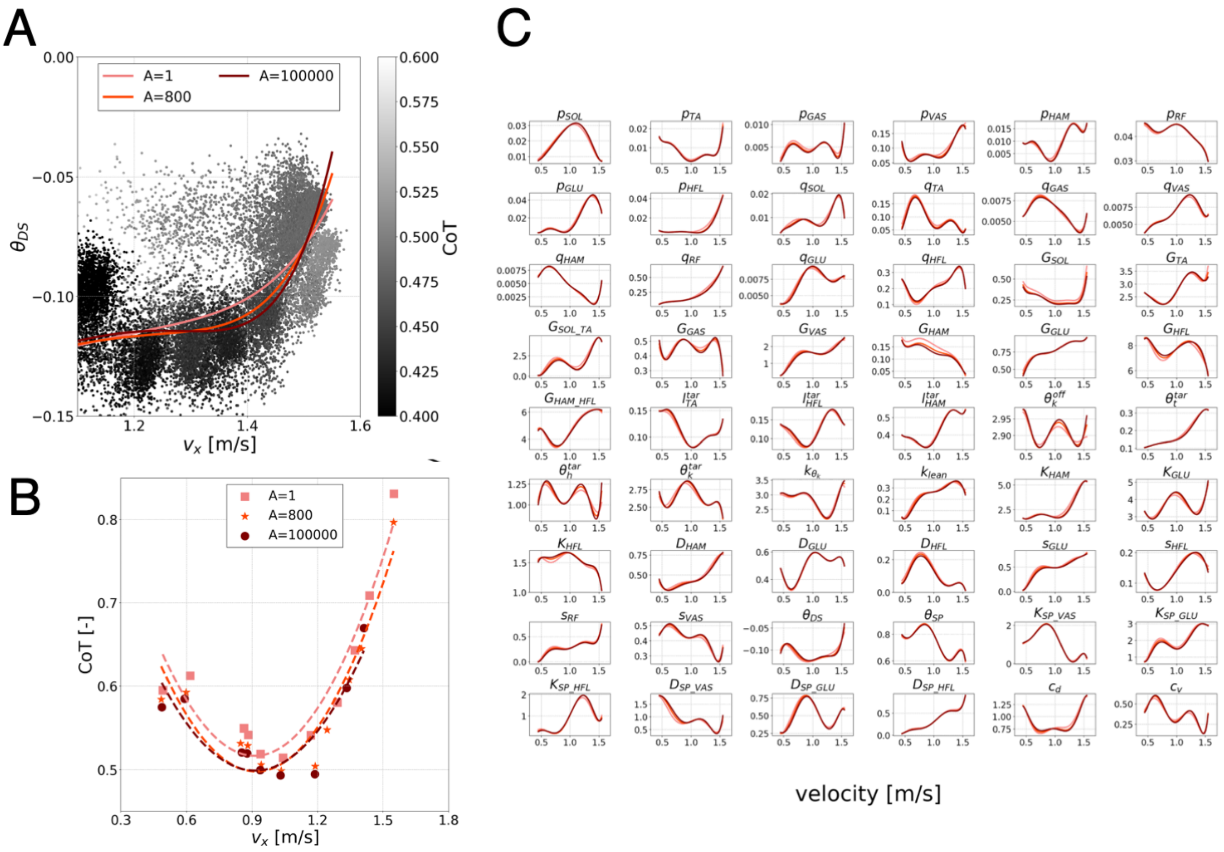

Fig 7 (previous manuscript). Contribution of the PWLS to gait generation. A. Regression curves of θ_{DS} with different A values. A in Eq.(67) determines the bias strength toward favorable data points. When $A = 1$, the regression curves are the same as those derived by the normal least squares method. **B. Estimated CoT curves for generated gaits with different A values.** These curves were calculated increasing target velocity v_x^{tar} from 0.5 to 1.5 m/s at 0.1 m/s intervals. When $A = 100,000$, the model fell down for $v_x^{tar} = 1.2$ and $1.5\,\text{m/s}$. **C. The optimized polynomial functions of each control parameter, P_i, with different A values.** The pink lines correspond to $A = 1$, orange lines to $A = 800$, and brown lines to $A = 100,000$.

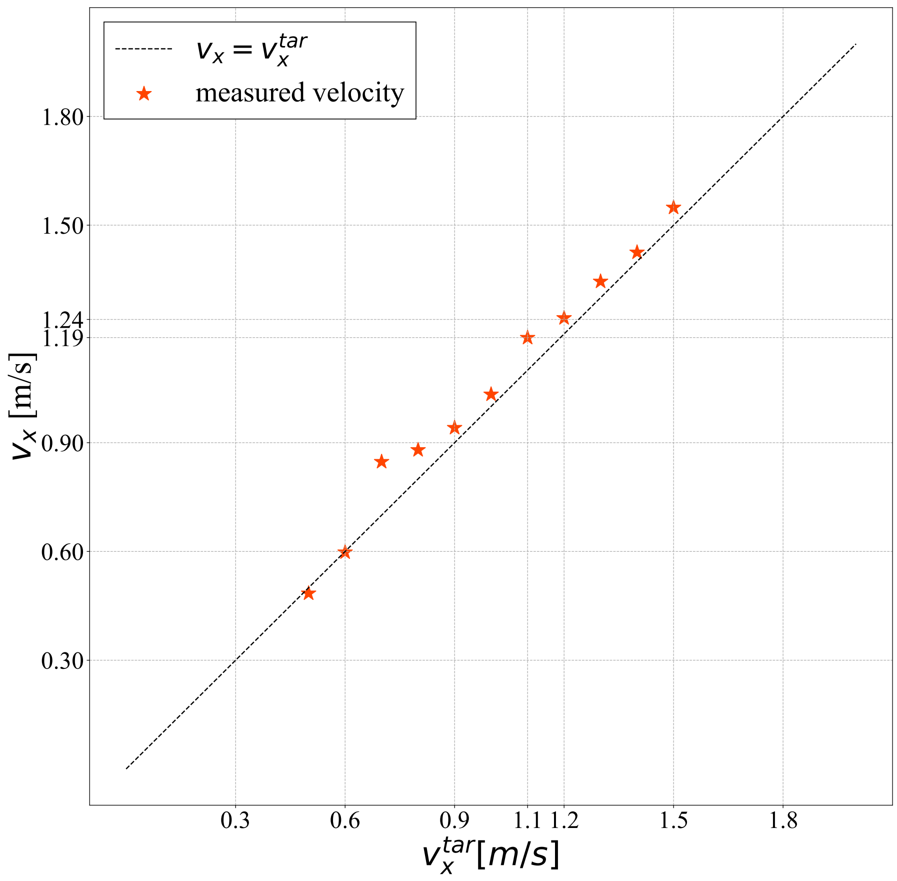

Fig 3. (previous supplementary material). The actual generated walking velocites v_x for input target velocities v_x^{tar}
