# Supplementary material for: Identifying essential factors for energy-efficient walking control across a wide range of velocities in reflex-based musculoskeletal systems
Source: PLoS Comput Biol. 2024 Jan 19;20(1):e1011771. doi: 10.1371/journal.pcbi.1011771 (PMC10798509; doi:10.1371/journal.pcbi.1011771)
Supplement: Attachment — Submitted filename: renamed_b3371.pdf [file pcbi.1011771.s004.pdf]

November 19th, 2023

Cover Letter Describing Our Responses to Editorial Decision (PCOMPBIOL-D-23-00582) from PLOS Computational Biology

Dear Associate Professor James O'Dwyer and Reviewers:

We extend our heartfelt gratitude for considering our manuscript (PCOMPBIOL-D-23-00582) for publication in your esteemed journal *PLOS Computational Biology.* We are also appreciative of the opportunity to engage in a constructive discussion with the reviewers.

The insightful comments provided by the reviewers have been instrumental in enhancing the quality of this manuscript. Please find below our point-by-point responses to the issues raised by the reviewer. We hope that the newly revised version of the manuscript meets the standards for publication in your journal.

We are grateful once again for your time and consideration. We look forward to hearing from you.

Sincerely,
Shunsuke Koseki

Dept. of Robotics, Graduate School of Engineering,
Tohoku University
6-6-01 Aoba, Aramaki, Aoba-ku, Sendai 980-8579, Japan
E-mail address: shunsuke.koseki.q4[at]dc.tohoku.ac.jp

Title: Identifying essential factors for energy-efficient walking control across a wide range of velocities in reflex-based musculoskeletal systems

Authors: Shunsuke Koseki[1], Mitsuhiro Hayashibe[1], and Dai Owaki[1]

Affiliations:

[1] Department of Robotics, Graduate School of Engineering, Tohoku University, Sendai 980-8579, Japan.

Please find below our point-by-point responses to the issues raised by the reviewers. We hope that the revised manuscript is now suitable for publication in your journal.

Comments from Reviewer #1

Red font: Reviewer's comments
Black font: Authors' comments
Blue font: Revised or newly added sentences in the revised paper

Comment 1

The authors refer to the model of Wang as the basis for their analysis. Wang stated its purpose to be "Through comparisons to kinematic and torque data of human walking, we show that our results adopt a human-like torque generation strategy while producing kinematic data significantly closer to humans than previous work." (Wang 2012), which he arguably met (comp. fig. 6 in Wang 2012).
The authors of this paper seek to implement velocity control in the neuro-muscular model which has been done before, e.g. by Dzeladini in 2014 (10.3389/fnhum.2014.00371, cited by the authors), which in my opinion does not demonstrate problems with speed regulation, as claimed by the authors. The reviewer acknowledges the effort of the authors to address the reviewer's concern with their massive compilation of material. However, the cost of the authors' efforts to control velocity is the loss of human-like walking dynamics, as detailed below. This indicates issues with the formulation of the cost function and makes the results no less questionable than before.
The authors summary "[...] Overshoots and undershoots were observed in the measured GRF...[]" largely understates the dissimilarities of the gait. This leaves a model with a high number of parameters optimized for an unphysiological mode of locomotion which renders the insights questionable. Some of the references do not support the authors claims (see below). Some of the authors' added content raises more questions than it answers, e.g., how is the physical validity of the MuJoCo caluclations etablished? To my knowledge the collision model in MuJoCo is an approximation for faster computation but not an accurate physical contact model. The general question of my comment 3 remains - why did the author's choose the complex model of Wang instead of the simpler, original model of Geyer?

Response to comment 1:

We appreciate your careful review. The reviewer's comments were exceptionally insightful and provided us with a more essential perspective of our study. We here respond to the reviewer's questions below and reemphasize our claims.

Reviewer's questions

1. Formulation of the cost function,
2. Physical validity of the MuJoCo simulator, and
3. The reason why we used Wang's model instead of Geyer's model

1: Formulation of the cost function

As commented by the reviewer and described in our Discussion section, adding other terms to the cost function could potentially improve the generated ankle joint kinematics, resulting in a gait more closely resembling that of humans. In Wang's study, some task terms were set in the cost function to generate a human-like gait, such as the minimization of maximum head velocity in parallel to the ground and validation of stability through foot contact. These task terms were more weighted in Wang's study compared to those associated with energy efficiency. Conversely, we employed the cost function with minimal task terms to generate a human-like gait,

giving more weight to the term relevant to energy efficiency. Consequently, it facilitated the identification of factors significantly influencing the energy efficiency of the generated gait over a wide range of walking velocities. We have added this explanation to the **Discussion**.

2: Physical validity of the MuJoCo simulator

MuJoCo (Multi-Joint Dynamics with Contact) is a physics engine developed for robotics and biomechanics research with fast computational speed while ensuring the validity of the physical calculations. To the best of our knowledge, MuJoCo has a more sophisticated collision model than other physics engines such as OpenSim, which is often used in biomechanics research and does not include shortcuts to stabilize computation in the simulations. More specifically, OpenSim models contact with the ground by using a spring-damper model. This approach requires using smaller timesteps to prevent the model foot from penetrating the ground, which increases computational cost. However, MuJoCo introduces several formulations of the physics of contact, which allows for more efficient calculations and versatile contact behaviors. Therefore, MuJoCo is widely used in the field of robotics, particularly in the context of locomotion, where interactions between the environment and the body are pivotal. OpenSim offers anatomically precise human musculoskeletal models. Nonetheless, as recent studies (Ikkala et al., 2022, ICNR, Wang et al., 2022, ICRA) have aimed to transfer OpenSim models to MuJoCo to improve computational speed and use versatile contact behaviors, MuJoCo is an acceptable physics engine with physical validity involving contact. We have added these explanations to the **Generating walking through reflex-based control; Musculoskeletal model**

3: The reason why we used Wang's model instead of Geyer's model

We used Wang's model instead of Geyer's original model because it can maintain physiological walking adaptation mechanisms. As described in the previous cover letter, Wang's controller is based on Geyer's model, with additional control laws integrated to adjust the target hip joint angle (SIMBICON) and to fix the hip and knee joints prepared for the heel strike. While these added control laws result in an increased number of control parameters, they more robustly yield gaits. Consequently, it can manage non-steady states during transitions in walking velocities. Moreover, the extended model can maintain the biomechanical explanatory ability essential to discussing gait adaptation mechanisms, which may be lost through simplification. We have added these explanations to the **Generating walking through reflex-based control; Reflex-based control**.

Based on the above answers, we believe that the designed cost function and employed reflex-based control are both suitable for this study, and that MuJoCo is an acceptable physics engine with physical validity involving contact. Moreover, we do not consider our generated gait as implausible because of the collision model, as carefully described in response 2 below. Rather, the gait that can only be reproduced by the carefully designed collision model would be extremely dependent on the interaction dynamics with the ground. Humans generate walking robustly in diverse environments. Therefore, we believe that an optimizing approach that is too similar to human gait in a limited environment risks losing the essence of the gait generation mechanism.

Discussion, Lines 492-497

> ... Third, we designed the cost function employed in this study to have minimal task terms to generate a human-like gait with more weight on the energy efficiency-related term. This approach optimized the control parameters to improve energy efficiency, consequently allowing the identification of factors that are significant to the energy efficiency of the generated gait over a wide range of walking velocities. ...

Generating walking through reflex-based control: Musculoskeletal model, Lines 67-79

> A two-dimensional musculoskeletal model with a height of 180 cm and weight of 80 kg was employed in this study, as illustrated in Fig 1A. Detailed parameters are listed in Table 1. The model was constructed within a MuJoCo simulation environment [46]. We employed Mujoco because of its ability to conduct simulations at a relatively low computational cost while ensuring the validity of the physical calculations. Many physics engines such as OpenSim, which is often used in biomechanics research, models contact with the ground by using a spring-damper model. This approach requires using smaller timesteps to prevent the model foot from penetrating the ground, which increases computational cost. MuJoCo introduces several formulations of the physics of contact. This allows for more efficient calculations and versatile contact behaviors. As recent studies [47,48] have aimed to transfer OpenSim models to MuJoCo to improve computational speed and use versatile contact behaviors, MuJoCo is an acceptable physics engine with physical validity involving contact. ...

Generating walking through reflex-based control: Reflex-based control, Lines 117-129

The reflex-based controller employed in this study is identical in principle to that of Wang et al. [31]. This controller is based on the Geyer model and incorporates additional control laws to adjust the target hip joint angle and to fix the hip and knee joints prepared for the heel strike. Consequently, it more robustly produces gaits and can manage non-steady states during transitions in walking velocities. Moreover, the extended model can maintain the biomechanical explanatory ability essential to discussing gait adaptation mechanisms, which may be lost through simplification. We did not make any ad-hoc adjustments to the basic reflex-based control model to ensure optimal simulation performance. The controller computes the muscle stimulation, denoted as u_i, for each muscle, i, by incorporating sensory feedback with a time delay of Δt as input. The control law switches depending on whether the leg is in the stance or swing phase. Moreover, additional stimulation is introduced during the late stance and late swing phases. ...

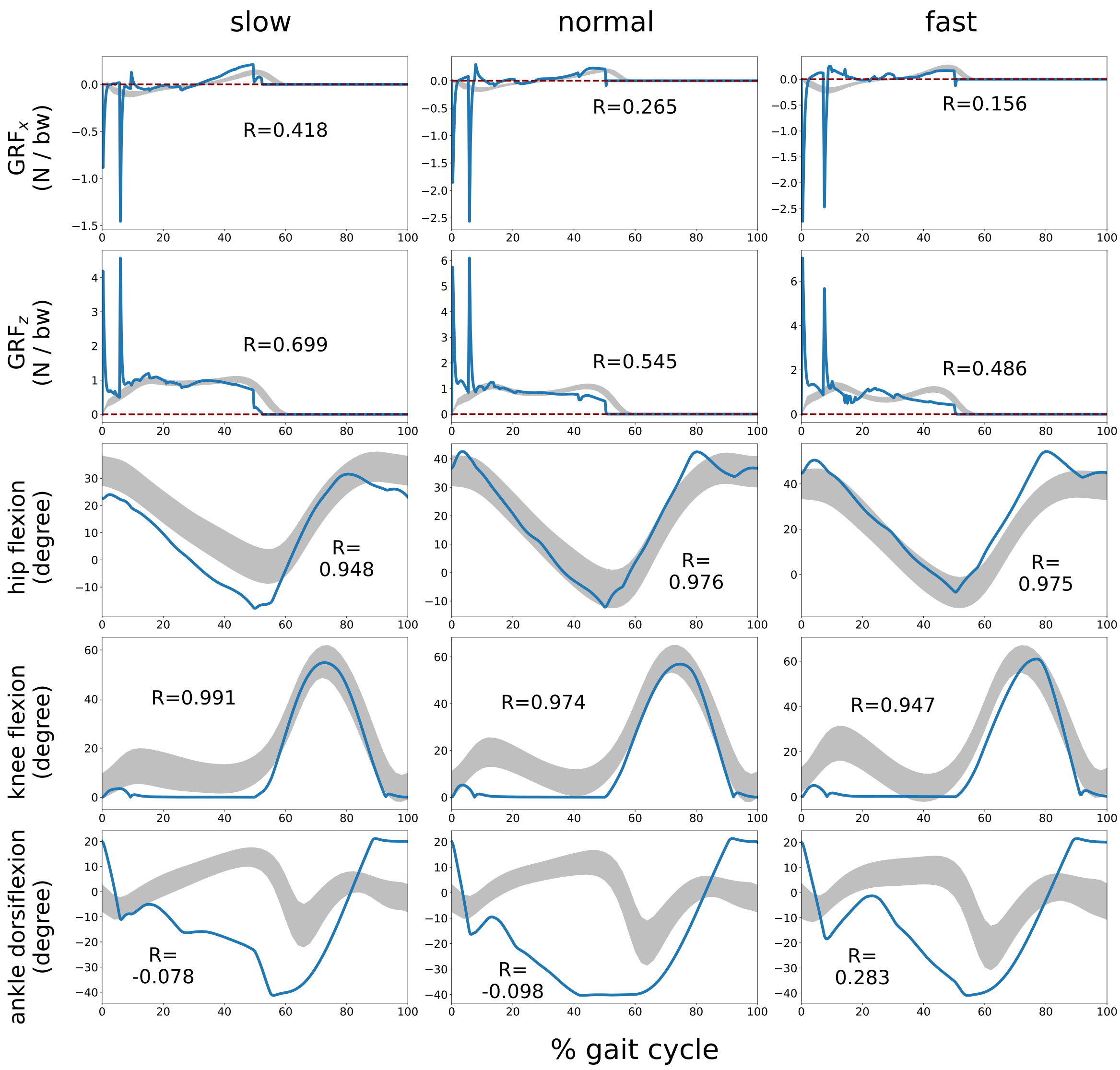

Fig 8. GRFs and kinematics of the generated gait. v_x^{tar} was set to 0.9 (slow), 1.25 (normal), and 1.6 m/s (fast). Blue lines depict the generated gait, while gray lines represent those of humans (mean±1 s.d.) [57]. GRF values are normalized by body weight. R denotes the cross-correlation value.

Comment 2

Specifically: Fig. 8 Depicted GRFs and ankle angle trajectories substantially differ from human walking. Negative R-values for ankle range of motion are discussed with "At ankle joints, the cross-correlation values of ankle dorsiflexion were very low. The previous study also showed low cross-correlation values at ankle joints when optimized to minimize energetic cost [29]." (line 457) - however [29] Song and Geyer 2019 do not show ankle range of motion cross-correlations in their study; in addition, "low" is very different from negative R-values. Vertical GRFs show no double hump profile and several spikes in the initial phase of gait which should be addressed. Horizontal GRFs also show extreme peaks in the beginning which is neither feasible nor physiological.

Response to Comment 2:

First, for the following sentence, "At ankle joints, the cross-correlation values of ankle dorsiflexion were low; the previous study also showed low cross-correlation values at ankle joints when optimized to minimize energetic cost [29]," we cited the wrong paper. The correct reference was "Song S, Geyer H. A neural circuitry that emphasizes spinal feedback generates diverse behaviors in human locomotion. J Physiol. 2015;593(16): 3493–3511." [34] in the manuscript. Fig 4 in [34] shows a comparison between the generated gait and that of humans.

Second, as the reviewer pointed out, "low (=no correlation)" and "negative" hold distinct implications in the context of R values.

Subsequently, we observed two extreme peaks in GRF_x and GRF_z. The former peak occurred at the heel strike, and the latter peak occurred at the toe-off of the contralateral leg. These extreme peaks in the GRF were the results of the high-impedance ground. Fig. R1 shows a comparison of the GRF during walking on high-impedance (as presented in our manuscript) and low-impedance ground. As shown in the figure, the extreme peaks in the GRF were reduced at the low-impedance ground. However, as shown in Fig. R2, on the low-impedance ground, the model foot penetrated the ground. Accordingly, we used a high-impedance ground to prevent foot penetration into the ground. Although the extreme GRF peaks are not biomechanically meaningful, we strongly believe that it is difficult to accurately model contact in a simulation environment (Todorov, 2014, ICRA, Castro et al., 2022, IEEE Transactions on Robotics). Moreover, we did not observe abrupt or significant changes in joint kinematics attributable to these extreme GRF peaks. Thus, we conclude that the effects of the extreme peaks of GRF on walking in the musculoskeletal model do not negatively affect the biomechanical meaning of the findings obtained in this study.

GRF_z of the generated gait did not exhibit a clear double hump profile. As previously mentioned, it is difficult to accurately represent contact in the simulation environment. Even in the previous study that employed reflex-based control to generate walking, a clear double hump profile in GRFz was not observed (Song et al., 2015, The Journal of physiology). From this perspective, the absence of a clear double hump profile in GRF_z alone did not suggest the conclusion that the results were not derived from proper gait mechanics. Improving the reproduction of the double hump profile in GRFz could be achieved, e.g., by reducing the ground impedance. The R value of GRFz on a low-impedance ground was larger than that of the high-impedance ground, as shown in Fig. R2. To prevent the model foot from penetrating the ground, we did not reduce the ground impedance.

In summary, the extreme peaks observed in GRF and the lack of a clear double hump profile in GRF_z were primarily a result of the high-impedance ground. Given that these extreme peaks in GRF do not seem to have a substantial impact on kinematics and agreeing with the challenges in accurately modeling contact in the simulation environment, we believe that the influence of these extreme peaks in GRF on the movement of the musculoskeletal model is minor and feasible for the biomechanical explanations.

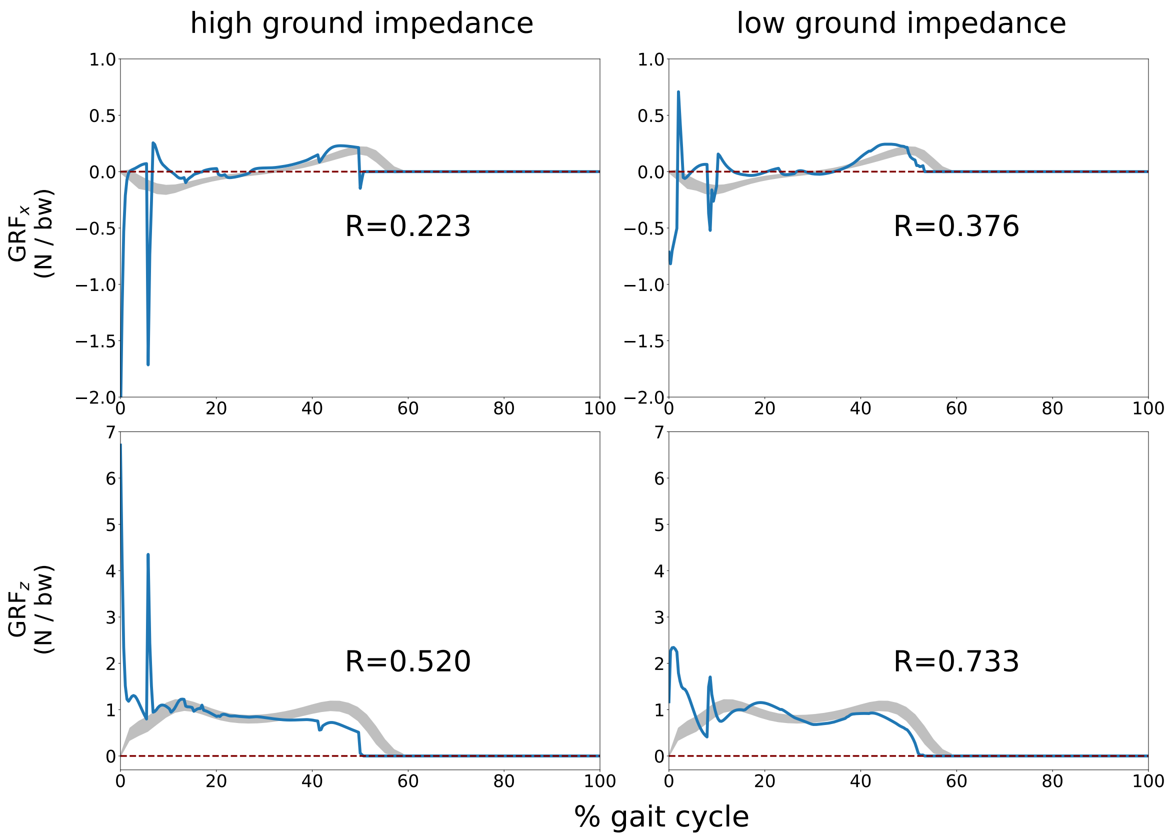

Fig R1. Comparison of GRF when generating a gait on a high-impedance ground (as presented in our manuscript) and a low-impedance ground. v_x^{tar} was set to 1.25. Blue lines depict the generated gait, while gray lines represent those of humans (mean±1 s.d.). We normalized GRF values with body weight. R denotes the cross-correlation value.

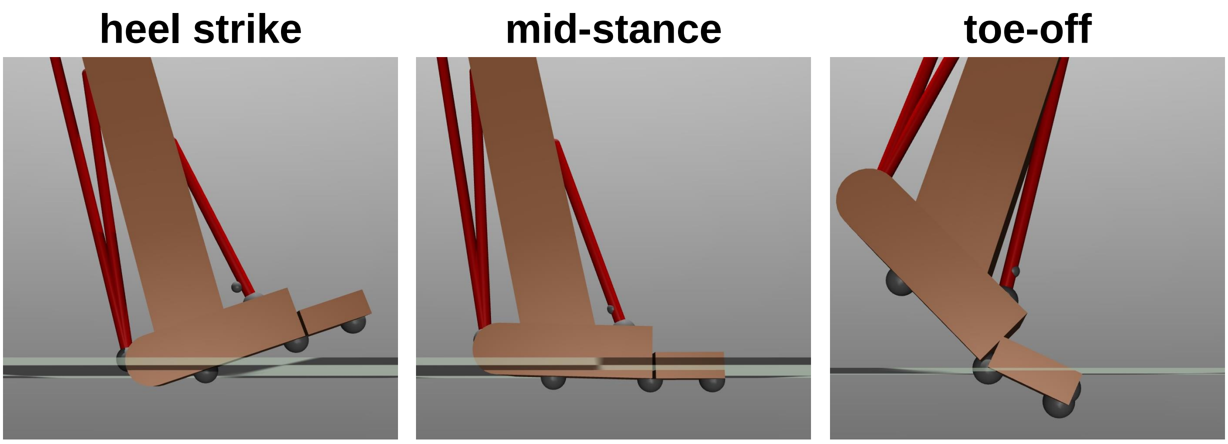

Fig R2. Foot penetrating the ground on the low-impedance ground.

Discussion, Lines 472-491

The results of this paper have some limitations concerning similarity to human walking. First, overshoots and undershoots were observed in the measured GRFs (Fig 8) and time evolution of the walking velocities (Fig 9). In the GRF profiles, we found two undershoots in GRF_x and two overshoots in GRF_z. The first occurred at heel strike, and the second occurred at toe-off of the contralateral leg. In the time evolution of the walking velocities, we observed overshoots at toe-off and undershoots at heel strike. These overshoots and undershoots can be suppressed by using a lower-impedance ground. However, employing a lower-impedance ground resulted in foot penetration into the ground (refer to S1 Appendix). While the extreme GRF peaks are not biomechanically meaningful, we strongly believe that it is difficult to accurately model contact in a simulation environment [67,68]. Moreover, we did not observe abrupt or significant changes in joint kinematics attributable to these extreme GRF peaks. Thus, given the difficulty of modeling contact in the simulation environment, the extreme peaks in GRF do not significantly affect kinematics. We conclude that the effects of the extreme peaks of GRF on walking in the musculoskeletal model do not negatively affect the biomechanical meaning of the findings obtained in this study. Second, the cross-correlation values of ankle dorsiflexion were close to 0 compared to hip and knee joints, as shown in Fig 8. At knee joints, the model straightened the knee earlier in the stance phase compared to humans. This strategy is known to generate more efficient solutions in gait optimization ~~[29]~~[34]. The previous study also showed low cross-correlation values at ankle joints when optimized to minimize energetic cost ~~[29]~~[34]. ...

Comment 3

Fig. 11 suggests that the A-value is a central property of the optimization, however the explanation of its meaning in line 276 ("Therefore, A determines the strength of the bias toward higher-performing data points.") is not helpful as is the lackluster explanation of PWLS in lines 280ff. Side note: for M=125, 2M+1 is 251 not 250. However I do not understand what the sentence in l.274 is supposed to mean.

Response to Comment 3:

To make the proposed PWLS algorithm easier to understand, we have revised the manuscript as follows: Additionally, as you indicated, the calculation result of $2M+1$ was incorrect. We have corrected it.

Modulation of control parameters:
Design of weight parameters β, Lines 274-291

$\beta_i \in \boldsymbol{\beta}$ is the performance evaluated in the PWLS to weigh corresponding data i. In this study, we evaluate the performance of each data point in terms of energy efficiency using the CoT. We design β such that it has a higher value when the CoT is low. Because the energy consumed in human walking depends on speed [55], it is unreasonable to compare CoT values between data with widely different speeds. Thus, we calculate β relative to the average CoT measured around the generated walking speed. Because the dataset, $\{(v_{x\,1}, \boldsymbol{Y}_1, \mathrm{CoT}_1), \ldots, (v_{x\,i}, \boldsymbol{Y}_i, \mathrm{CoT}_i), \ldots, (v_{x\,n}, \boldsymbol{Y}_n, \mathrm{CoT}_n)\}$, is sorted by speed, the average CoT around the jth data point, $\overline{\mathrm{CoT}}_j$, is calculated as follows:

$$\overline{\mathrm{CoT}}_i = \frac{\mathrm{CoT}_{i-M} + \mathrm{CoT}_{i-M+1} + \ldots + \mathrm{CoT}_{i+M}}{2M+1}. \quad (1)$$

In this study, M was set to 125, giving $2M+1 = 251$, which is approximately 0.5% of the control parameter sets collected in the dataset. Using the average CoT value around the ith data point, $\overline{\mathrm{CoT}}_i$, we defined the weight for data i, β_i, as follows:

$$\beta_i = A^{(1-\frac{\mathrm{CoT}_i}{\overline{\mathrm{CoT}}_i})}, \quad (2)$$

where $A \geq 1$ denotes a constant. $\beta_i > 1$ indicates that the data are evaluated as favorable data and $\beta_i < 1$ as unfavorable data. For example, when $\mathrm{CoT}_i < \overline{\mathrm{CoT}}_i$ (i.e., generated gait was more energy-efficient), $(1 - \frac{\mathrm{CoT}_j}{\overline{\mathrm{CoT}}_j})$ is calculated above 0, and this yielded $\beta_i > 1$. A smaller CoTi value in comparison to the average CoT value, $\overline{\mathrm{CoT}}i$, results in the exponential increase of β_i based on the A value. Therefore, the parameter A determines the strength of the bias toward higher-performing data points, i.e., a larger A value indicates a greater bias toward favorable data.

Comment 4

Reflecting on the authors conclusion, they failed to demonstrate the applicability of the optimized solutions and achieve parameter modulation while loosing fundamental properties that characterize walking. This largely reduces the applicability of the gained insights. I would suggest to revisit the underlying model and try to reduce the number of parameters rather than extend them. First and foremost, the resulting gait should aim at resembling human walking on a kinetic and kinematic level in order to draw conclusions concerning adaptation mechanisms.

Response to Comment 4:

Although we agree that reducing the number of control parameters would be essential and helpful for understanding the mechanism underlying walking, as carefully explained in response to comment 1, we used Wang's extended model rather than Geyer's original model to robustly generate walking without losing biomechanical explanatory ability. Additionally, as the reviewer indicated, the generated walking deviates from human walking in some aspects. The R value of ankle joint kinematics being close to 0, the extreme peaks in GRF, and the unclear double hump profile in GRF_z were observed. However, the gait has a number of characteristics that demonstrate its biomechanical validity in comparison to the human gait, as shown below:

1. The R values of the hip and knee kinematics were close to 1 (R > 0.94 in all cases), indicating that they were highly correlated with humans.
2. The trajectory of the hip segment height from the ground exhibited a sinusoidal pattern (see Fig. R3 below).
3. Heel strike and toe-off events were clearly observed (see attached movie file).
4. There was a quadratic relationship between walking velocities and CoT (Fig 10).

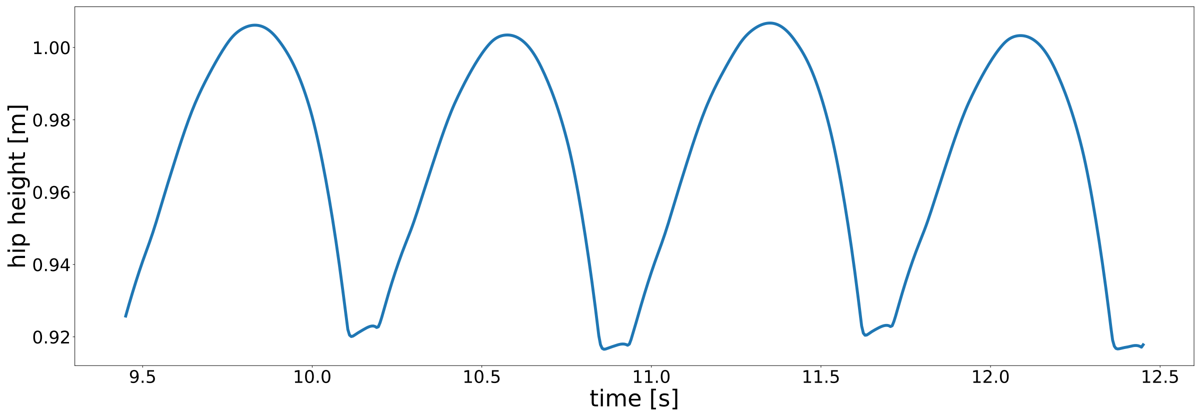

Fig R3. Trajectory of the hip segment height from the ground. v_x^{tar} was set to 1.25 m/s. The curve was sinusoidal, corresponding to humans.

These features are similar to those of human walking from a biomechanical perspective, proving that our simulations do not compromise the dynamically reasonable properties of human walking. We added this explanation to **Discussion** as follows. We hope that the revised manuscript has been sufficiently improved by our modifications.

Discussion, Lines 460-471

The generated walking has several characteristics that demonstrate its biomechanical validity in comparison to the human gait. (i) the R values of the hip and knee kinematics were close to 1 ($R > 0.94$ in all cases); (ii) The trajectory of the hip segment height from the ground exhibited a sinusoidal pattern (see Fig. S7 in S1 Appendix) [65]; (iii) we observed both heel strike and toe-off events [66] (see attached movie file); and (iv) a quadratic relationship between walking velocities and CoT [55] (Fig 10). These features are similar to those of human walking from a biomechanical perspective, proving that our simulations do not compromise the dynamically reasonable properties of human walking. Although we agree that reducing the number of control parameters would be

essential and helpful for understanding the mechanism underlying walking, we used Wang's extended model rather than Geyer's original model to robustly generate walking without losing biomechanical explanatory ability.
